# Inhibitor fluorination pattern modulates protein surface dynamics and chemically induced dimerization

**DOI:** 10.1039/d6sc05625c

**Published:** 2026-09-04

**Authors:** Eric Schwegler, Jean-Martin Harder, Marco D. Preuss, Charlotte Guhl, Shuaibing Zhang, Annika Wagner, Nicole Bader, Pierre Stallforth, Hermann Schindelin, Till Opatz, Markus Lakemeyer, Ute A. Hellmich

**Affiliations:** a Institute for Organic Chemistry and Macromolecular Chemistry, Friedrich Schiller University Jena 07743 Jena Germany ute.hellmich@uni-jena.de; b Department of Chemistry, Johannes-Gutenberg University Mainz 55128 Mainz Germany; c Department of Paleobiotechnology, Leibniz Institute for Natural Product Research and Infection Biology (HKI) 07745 Jena Germany; d Rudolf Virchow Center for Integrative and Translational Bioimaging, Julius-Maximilians-University 97080 Würzburg Germany; e Cluster of Excellence Balance of the Microverse, Friedrich Schiller University Jena 07743 Jena Germany; f Center for Biomolecular Magnetic Resonance (BMRZ), Goethe-University Frankfurt Max-von-Laue Str. 9 60438 Frankfurt am Main Germany

## Abstract

Chemically induced proximity is a powerful strategy to regulate cellular processes using small-molecule ligands that act as “molecular glues” to influence the lifetime, localization, and function of biological targets. However, the structure–activity relationships governing such inducible interactions remain elusive. Here, using a series of self-assembling homodimerizers that target an essential parasitic redox enzyme, we present a systematic, fluorination-guided strategy to tune induced protein homodimer affinity by two orders of magnitude. Combining NMR spectroscopy, MD simulations, chromatography, multi-angle light scattering, mass spectrometry, calorimetry, and functional assays, we demonstrate that the fluorination pattern of the dimerizer tunes homodimer affinity by modulating the conformational dynamics of both the bound ligand and the protein residues constituting the dimer interface. These findings establish a generalizable framework for understanding how ligand fluorination shapes protein surface dynamics and induced protein interactions. They further reveal how the fluorination pattern of molecular glues modulates protein assembly across biologically relevant affinity ranges, providing mechanistic principles for the rational design of proximity-inducing molecules.

## Abstract 2

Fluorination was used to explore structure–activity relationships of covalent molecular glues for a parasitic enzyme. By shaping the dynamic interplay between ligands and protein surface, fluorination tuned dimer affinity by two orders of magnitude.

## Graphical abstract

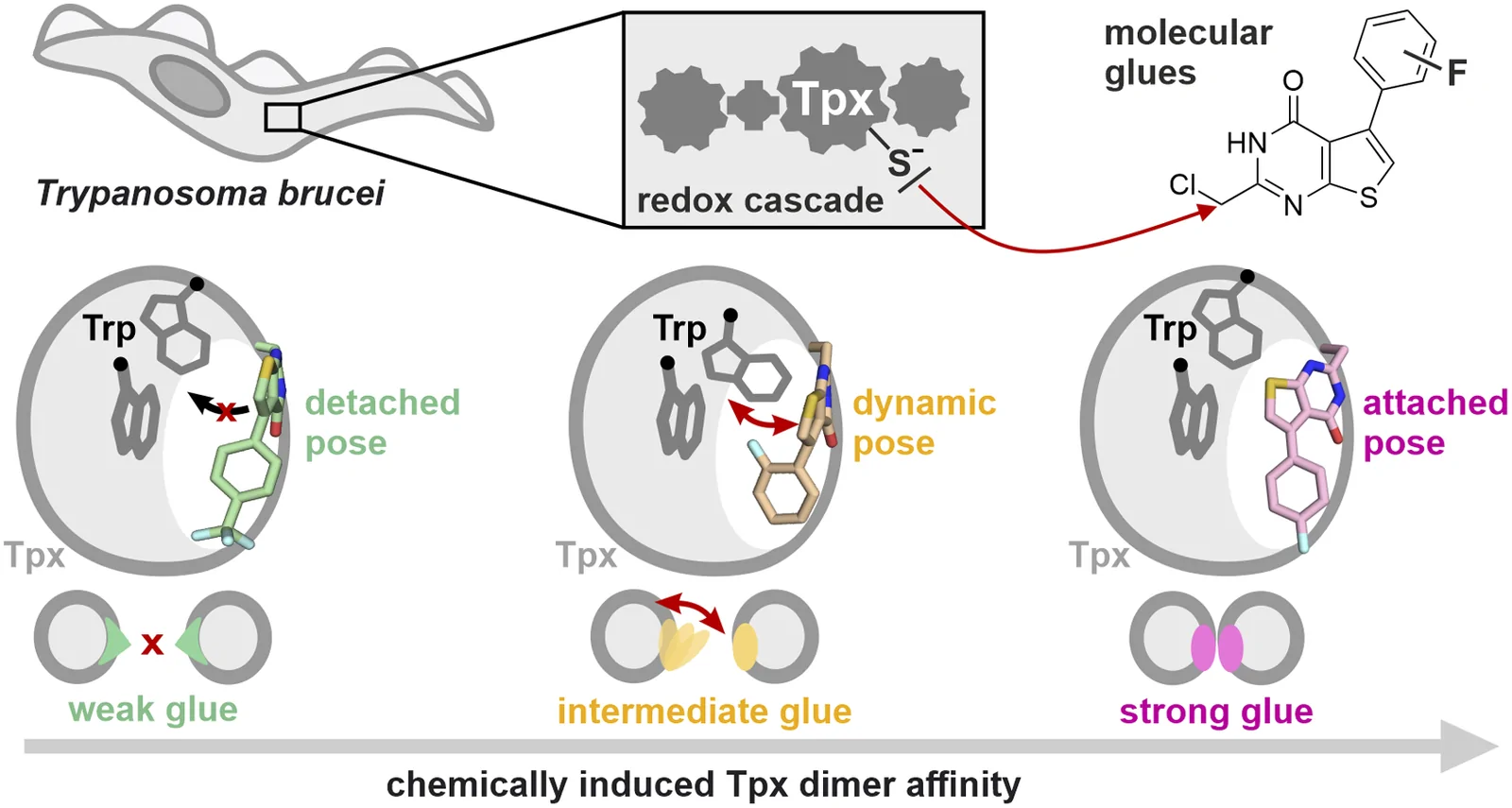

## Introduction

Chemically induced dimerization (CID) is a powerful tool to control the lifetime, localization and biological function of biomacromolecules *in vitro* and *in cellulo*.^1–3^ Proximity-inducing molecules like PROTACs (proteolysis targeting chimeras) and molecular glues have significantly expanded the range of druggable disease targets, including cancer and neurodegenerative, inflammatory, and infectious diseases.^2,4^ The dynamic interplay between a macromolecular target and its dimerizing ligand determines induced dimer affinity and is therefore central to any CID system. Nevertheless, most studies that have explored CID have focused on either ligand design or protein engineering, but did not pursue both approaches for the same system.^5^ Consequently, the molecular mechanisms by which subtle ligand modifications modulate dimer interface dynamics and ultimately CID efficiency remain poorly understood.

The oxidoreductase tryparedoxin (Tpx, Fig. S1) is an essential redox regulator in human pathogenic parasites called trypanosomatids.^6–8^ As part of an enzymatic redox cascade, Tpx transfers electrons *via* thiol–disulfide exchange to vital downstream enzymes like RNA reductases or peroxidases (Px, Fig. 1a, inset).^6–8^ Tpx is considered a drug target in multiple studies.^7–9^ The nucleophilic active site of Tpx from *Trypanosoma brucei*, the causative agent of African Sleeping Sickness, covalently binds electrophilic compounds which inhibit the protein's redox activity.^10^ One of these covalent inhibitors, 2-(chloromethyl)-5-(4-fluorophenyl)-thieno[2,3-*d*]pyrimidin-4(3*H*)-one (*para*-CFT, 1, Fig. 1b), acts as molecular glue for *T. brucei* Tpx and is one of the smallest protein dimerizers known to date (Fig. 1c).^11,12^ Thieno[2,3-*d*]pyrimidinones like *para*-CFT are synthetically readily accessible and tolerate diverse substitution patterns at multiple positions, making them suitable starting points for systematic interaction studies and rational ligand design.^13^

**Fig. 1:**
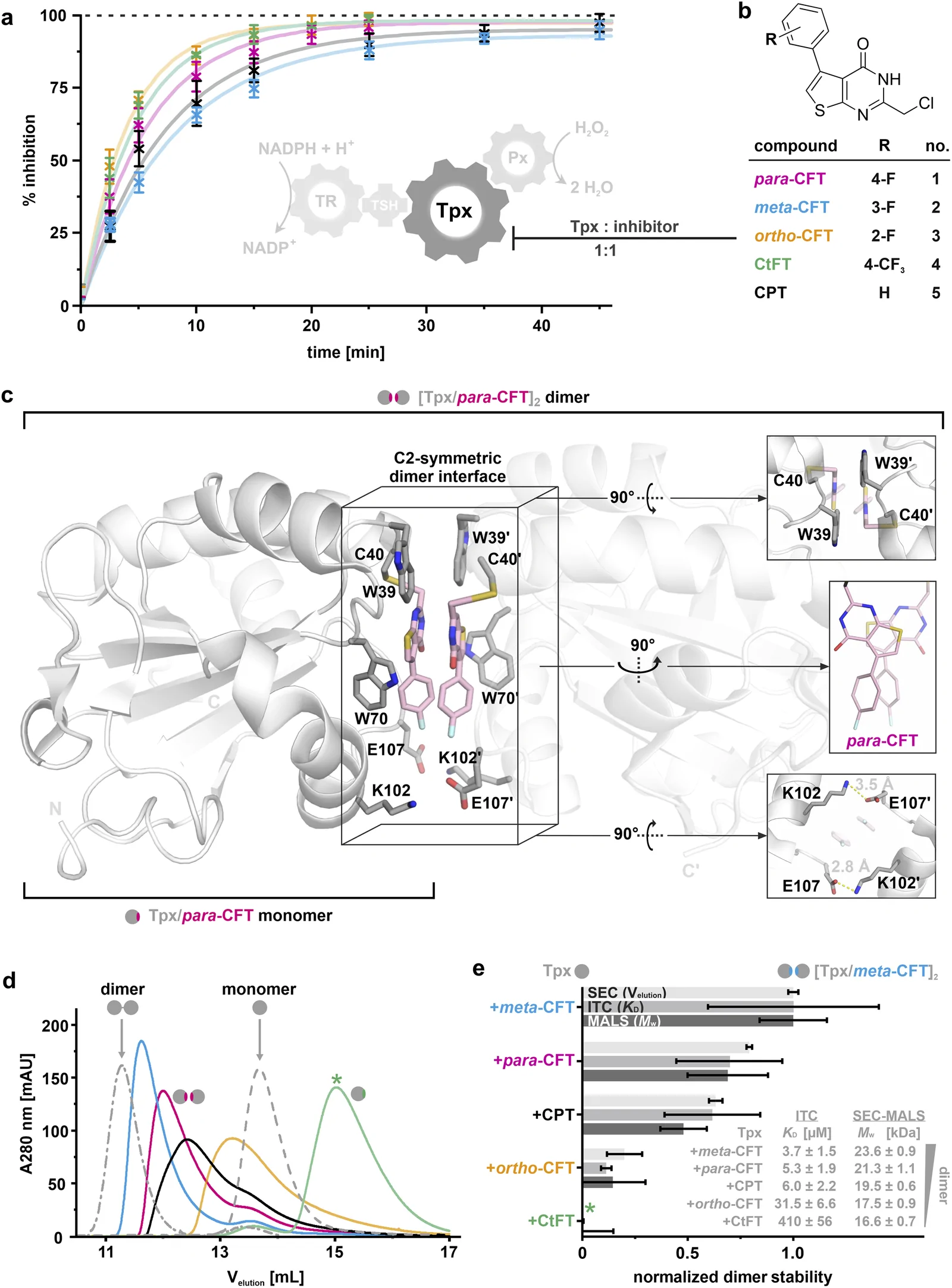
The inhibitor fluorination pattern modulates Tpx inactivation kinetics and chemically induced dimerization. (a) Time-resolved inhibition of Tpx by equimolar amounts of compounds 1–5. The *T. brucei* cytosolic peroxide clearance cascade was reconstituted *in vitro* (TR: trypanothione reductase: TSH: trypanothione; Tpx: tryparedoxin, Px: peroxidase), and NADPH consumption was followed photometrically (*n* = 3, mean ± standard deviations, fitted). (b) A thienopyrimidinone scaffold-based fluorine walk library inspired by *para*-CFT, a covalent inhibitor and dimerizer of tryparedoxin (Tpx) from *T. brucei*.^10–12^ Derivatives are denoted *meta*-CFT (2-(chloromethyl)-5-(3-fluorophenyl)thieno[2,3-*d*]pyrimidin-4(3*H*)-one), *ortho*-CFT (2-(chloromethyl)-5-(2-fluorophenyl)thieno[2,3-*d*]-pyrimidin-4(3*H*)-one), CtFT (2-(chloromethyl)-5-(4-(trifluoromethyl)phenyl)thieno[2,3-*d*]pyrimidin-4(3*H*)-one), and CPT (2-(chloromethyl)-5-phenylthieno[2,3-*d*]pyrimidin-4(3*H*)-one). (c) X-ray structure of the *C*_2_-symmetric [Tpx/*para*-CFT]_2_ homodimer (PDB: 6GXG,^12^ chains A, B), highlighting key interface contacts. (d) SEC analysis of covalent Tpx/inhibitor complexes (100 µM analyte, colored traces). Unmodified Tpx monomers and BM(PEG)_2_-crosslinked Tpx dimers served as references (grey traces). CtFT (green trace) showed delayed elution due to increased column interactions (*) (Fig. S5). (e) Tpx dimer stability determined by analytical SEC (*n* = 3), SEC-MALS (*n* = 1), and dilution ITC (*n* = 3) (see Fig. S4 and S6). For comparison, the dimer with the highest affinity, [Tpx/*meta*-CFT]_2_, was set to 1 for normalization. ITC-derived *K*_D_ values (mean ± standard deviation), and SEC-MALS molecular weights (*M*_w_) (value ± measurement error) are shown. No SEC data for Tpx/CtFT is shown due to the analyte's aberrant running behavior (indicated with an asterisk, Fig. S5).

Fluorination is widely used to tune the desired physicochemical properties of pharmaceuticals and agrochemicals, including molecular interactions with biological targets, metabolic stability and pharmacokinetics.^14–19^ However, despite promising examples,^19^ the potential of small-molecule fluorination to tune CID systems has not been systematically explored. Here, inspired by so-called “Fluorine Walk” strategies,^14–17,19^ we investigated how the fluorination pattern of covalent thienopyrimidinone inhibitors controls chemically induced homodimerization of *T. brucei* Tpx.^10,20^

To this end, we varied the position of the fluorine substituent, exchanged it with a trifluoromethyl group, and generated an unfluorinated derivative (Fig. 1b). After confirming the inhibitory ability of all molecular glue analogues and their ability to covalently interact with Tpx using *in vitro* and *in vivo* activity assays, as well as mass spectrometry, we determined their ability to induce protein homodimerization. Because of the large differences in observed dimer affinities, we used size exclusion chromatography (SEC), multi-angle light scattering (MALS), and dilution isothermal titration calorimetry (ITC) as complementary approaches to benchmark the affinities of all compounds with sufficient confidence for subsequent mechanistic analyses. Finally, we determined the molecular basis of the observed differences in dimer affinity combining protein- and inhibitor-detected NMR with MD simulations. A non-dimerizing Tpx mutant allowed us to dissect ligand interactions with the individual Tpx protomer. Together, this integrated approach allowed an in depth analysis of chemically induced dimerization both from the perspective of the protein and the molecular glue.

We show that molecular glue fluorination can tune induced Tpx dimer affinity by two orders of magnitude. Beyond static structural snapshots, our results reveal how site-specific fluorination modulates protein–ligand interactions and dimer interface dynamics to tune chemically induced dimer affinity. Together, these findings demonstrate that fluorination of molecular glues is a promising approach to control protein–protein interactions.

## Results and discussion

### Inhibitor fluorination modulates enzyme inactivation kinetics

To explore the role of inhibitor fluorination for Tpx inactivation kinetics and chemically induced Tpx dimerization, we generated a small set of fluorinated thienopyrimidinones (1–5, Fig. 1b, see SI for inhibitor synthesis and analyses). The addition of Tpx-equimolar amounts of compounds 1–5 sufficed to fully inactivate the reconstituted *Trypanosoma brucei* redox cascade in a photometric activity assay (Fig. 1a). In line with Tpx-selective binding, half-equimolar dosage of inhibitors resulted in 50% inactivation of the cascade and no off-target binding was observed despite the high abundance of nucleophilic cysteines in the enzymatic cascade (Fig. S2 and S3a–e). Intact-protein mass spectrometry (MS) of Tpx WT and the active site mutants C40S and C43S confirmed that all inhibitors covalently bind to the nucleophilic active site residue C40 ^6^*via* the elimination of chloride, indicating an S_N_2-reaction mechanism (Table S1).

At Tpx-equimolar concentrations, *ortho*-CFT and CtFT (3, 4) caused the fastest, and *meta*-CFT (2) the slowest Tpx inactivation (Fig. 1a). To quantify irreversible inhibition kinetics, we determined the concentration-dependent inhibition constants (*k*_obs_) for the inhibitors, and derived the corresponding, concentration-independent second-order rate constants (*k*_2nd_).^21^ These differed by a factor of 2, with 112 ± 1 min^−1^ mM^−1^ for CtFT (4) to 56 ± 1 min^−1^ mM^−1^ for *meta*-CFT (2) (Fig. S3a–e). Due to the relatively fast inactivation kinetics, our experimental setup precluded us from obtaining *k*_obs_ at higher inhibitor concentrations, which would be required to observe saturation and to determine Tpx binding parameters *k*_inact_ and *K*_I_.^21^

In cell-based assays, all compounds showed comparable activity against bloodstream *T. brucei* parasites, with EC_50_ (24 h) values between 0.6 ± 0.3 µM for CPT (5) to 2.2 ± 0.6 µM for CtFT (4) (Fig. S2b and Table S2). The EC_50_ values in human embryonic kidney (HEK293) cells were moderately higher, resulting in selectivity indices (defined as the ratio of EC_50_ values for human and parasitic cells) marginally above 1 (Fig. S2b). Although this limited selectivity *in vivo* currently precludes further development as trypanocidal agents, all compounds specifically target the Tpx active site residue C40 *in vitro*, thus providing a well-defined system to dissect how fluorination modulates protein–ligand interactions and induced dimerization.

### Molecular glue fluorination pattern tunes protein dimer affinity

The incubation of *T. brucei* Tpx with thienopyrimidinone inhibitors 1–3 and 5 shifted the protein's elution volume in size exclusion chromatography (SEC) towards that of a cross-linked dimer reference, consistent with inhibitor-induced dimerization (Fig. 1d and S4). In line with a dynamic monomer–dimer equilibrium, elution volumes were concentration-dependent, and the chromatograms displayed peak tailing. Tpx incubation with compound CtFT (4), containing a CF_3_-group, led to the elution at a higher volume than unmodified, monomeric Tpx, a behavior attributable to secondary interactions with the SEC column matrix as this behavior was reversed with a more hydrophobic mobile phase (Fig. 1d, green trace and S5).

Since the atypical SEC running behavior of CtFT-bound Tpx precluded an unambiguous assessment of dimerization *via* SEC alone, we combined it with multi-angle light scattering (SEC-MALS). At 100 µM analyte concentration, inhibitors 1–3 and 5 induced Tpx dimerization, while Tpx with bound CtFT (4) remained monomeric (Fig. 1e and S4c). Dissociation constants (*K*_D_) of the chemically induced Tpx dimers were obtained using dilution ITC and corroborated a strong dependence between the inhibitor fluorination pattern and induced dimer affinity, spanning two orders of magnitude from *meta*-CFT (2, *K*_D_ = 3.7 ± 1.5 µM) to CtFT (4, *K*_D_ = 410 ± 56 µM) (Fig. 1e and S6).

Thus, combining analytical SEC, SEC-MALS, and ITC measurements, we found that the ability of inhibitors 1–5 to act as Tpx dimerizers increases as follows: CtFT (4) ≪ *ortho*-CFT (3) < CPT (5) < *para*-CFT (1) < *meta*-CFT (2). Notably, chemically induced dimer affinity and inhibition kinetics appeared to be inversely correlated (Fig. S3f), suggesting that *S*-alkylation of the Tpx C40 side chain with strong dimerizers (1, 2, 5) could require a higher activation energy than *S*-alkylation with weak dimerizers (3, 4), slowing down the binding process. Hence, we hypothesize that the S_N_2-transition state for strong dimerizers (1–2, 5) requires more pronounced structural rearrangements in the Tpx binding interface and is less stable than for weak dimerizers (3, 4).

### Intermolecular contacts are preserved and reflect dimer affinity

To investigate the structural consequences of inhibitor fluorination for the induced Tpx dimer interface, we initially aimed to co-crystallize Tpx with the new inhibitors 2–5. However, except for our previously determined Tpx/*para*-CFT dimer structure (PDB: 6GXG,^12^Fig. 1c), this was not successful. Instead, we used ^1^H, ^15^N-NMR spectroscopy to map intramolecular protein–inhibitor as well as intermolecular dimer contacts in solution.^22^ Taking advantage of previous Tpx backbone amide resonance assignments of the unmodified protein,^23^ we assigned the Tpx backbone amides in complex with all inhibitors (Fig. S7). Next, the inhibitor-induced chemical shift perturbations (CSPs) were mapped onto the Tpx/*para*-CFT dimer crystal structure (Fig. 2a and S8). In all cases, the same residues were affected, suggesting that compounds 1–5 all induce the formation of similar protein dimer interfaces. Most amide resonances of residues within the dimer interface displayed extensive line broadening as a direct consequence of ligand binding and the dynamic exchange between the monomeric and dimeric states. Other residues displayed linear chemical shift trajectories (shown for residues A11, L112, and R128 in Fig. 2b), which mirrored the relative dimer stability determined by SEC, SEC-MALS and ITC, indicative of a gradual increase in dimer affinity in dependence of the inhibitor fluorination pattern. Interestingly, amide resonances of Tpx residues which were not broadened, but, based on the crystallized Tpx/*para*-CFT complex^12^ (Fig. 1c) presumed to participate in direct inhibitor interactions, did not show the aforementioned linear chemical shift behavior (Fig. 2b, right boxes). Examples include the W70 side chain which interacts with the inhibitor's thiophene moiety *via* T-shaped π-interactions, and the E107 backbone which contacts the inhibitor's phenyl substituent (Fig. 1c).^12^ It is therefore tempting to interpret the chemical shift differences across inhibitors as direct evidence for fluorination pattern-induced differences in intramolecular inhibitor binding modes. Nonetheless, these signals may also be affected by dimerization. To further probe the interface of the chemically induced dimers, we thus complemented our NMR studies with MD simulations.

**Fig. 2:**
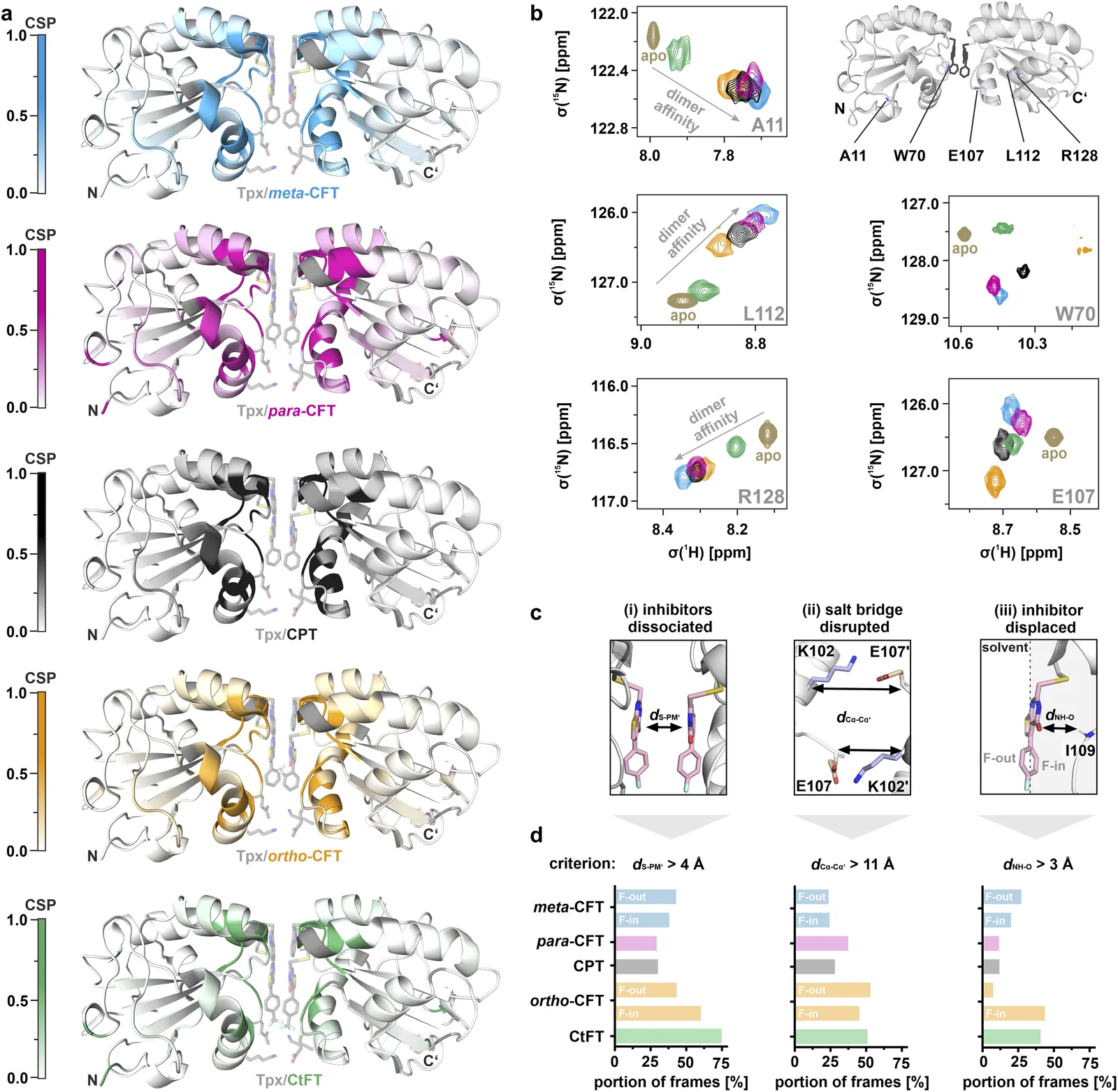
Chemically induced Tpx dimers share a conserved dimer interface with defined inter- and intramolecular contacts. (a) Chemical shift perturbations (CSP) for ^15^N-labeled Tpx upon binding of inhibitors 1–5, mapped onto the Tpx/*para*-CFT dimer X-ray structure (PDB: 6GXG,^12^ see also Fig. S7 and S8). (b) Selected resonances from ^1^H, ^15^N-HSQC spectra of Tpx in the reduced apo (sand) and inhibitor-bound states (colored). Most amide resonances in the dimer interface displayed line broadening or a linear trajectory matching induced dimer affinities, exemplarily shown for A11, L112 and R128. NH resonances of residues in immediate contact with the inhibitors, *e.g.* from the W70 side chain and the E107 backbone, did not show this trend as they are affected both by inhibitor binding and dimerization (see main text for details). (c) The dimer interface was probed by molecular dynamics (MD) simulations (*n* = 5, 100 ns per simulation, 2 fs per frame, movies 1–7). Tracking three key intra- and intermolecular contacts sufficed to correlate MD frame populations with measured dimer affinities (see Fig. 1e). Inhibitor dissociation (i), interchain salt bridge disruption (ii) and inhibitor displacement (iii) were monitored *via* parameters *d*_S–PM_, *d*_Cα–Cα′_, and *d*_NH–O_ (for details, see main text and SI). For simplicity, the different contacts are illustrated with *para*-CFT (1). (d) Portion of frames in MD simulations where *d*_S–PM_, *d*_Cα–Cα’_, and *d*_NH–O_ exceeded critical values and were thus incompatible with stable dimer formation.

To perform MD simulations with energetically favorable inhibitor conformations, we first used density-functional theory (DFT) calculations of the isolated inhibitors to assess the energy barriers associated with rotating the dihedral angle between the phenyl and thiophene moieties (Fig. S9a). All inhibitors (1–5) displayed two rotational barriers at torsion angles of 0° and ∼180°. For *ortho*-CFT (3), a steric clash between the inhibitor's fluorine and oxygen atoms resulted in a particularly high energetic barrier (∼80 kJ mol^−1^). For all molecules, we observed local energetic minima at dihedral angles of ∼48°. Since this matched with the 47° dihedral angle observed between the phenyl and thiophene rings in the crystal structure of Tpx-bound *para*-CFT (1) (Fig. 2a),^12^ and was in line with our crystal structures of the isolated inhibitors also showing non-planar conformations (Fig. S9b–d and Table S3),^28^ we considered a dihedral angle of 47° a suitable starting point for MD simulations of wildtype (WT) Tpx in complex with the inhibitor molecules. Of note, for Tpx-bound *meta*- and *ortho*-CFT (2, 3), which have an asymmetric phenyl substitution pattern as the fluorine substituent can either face towards the Tpx binding pocket (“F-in”) or the solvent (“F-out”, Fig. 2c, right panel and S10, movies 1–7), we defined two inhibitor orientations. Axial chirality for these Tpx/inhibitor complexes was retained throughout all simulations. A comparison between dimeric and monomeric Tpx WT/inhibitor complexes *in silico* showed that the conformational “trapping” of the inhibitor molecule in a “F-in” or “F-out” orientation is a result of Tpx protomer binding, not subsequent dimerization (Fig. S10 and movies 8–14).

A small set of inter- and intramolecular dimer interactions were observed in all MD simulations and were concurrently used to assess induced Tpx dimer stability *in silico*: (i) molecular glue stacking was evaluated by monitoring the distance between the thiophene sulfur and pyrimidinone centroid of the two sandwiched molecules (*d*_S–PM′_), (ii) intermolecular salt bridge formation between lysine residues 102 and glutamate residues 107 from opposing protomers was tracked by measuring the Cα distances (*d*_Cα–Cα′_), and (iii) the displacement of the inhibitor from the binding site was followed by recording the distance between the thienopyrimidinone oxygen and the I109 backbone NH (*d*_NH–O_) (Fig. 2c, d and movies 1–7). Exceeding critical thresholds for *d*_S–PM_, *d*_Cα–Cα′_ and *d*_NH–O_ was deemed to be incompatible with a stable dimer arrangement and interpreted as partial dimer dissociation (Fig. S11).

In agreement with our *in vitro* data, the weak dimerizers *ortho*-CFT and CtFT (3, 4) exhibited the largest portion of frames with “partially dissociated” interfaces *in silico* (Fig. 2d, orange and green bars). Notably, displacement of *ortho*-CFT (3) occurred more often for the “F-in” conformation than for the “F-out” conformation, suggesting that *ortho*-fluorination may directly perturb interactions with the Tpx binding pocket. Indeed, the unique dynamic behavior of Tpx-bound *ortho*-CFT (3) was corroborated by NMR spectroscopy both for the molecular glue using ^19^F NMR (see below), and the neighboring W70 indole by ^1^H, ^15^N-NMR (Fig. 2b, orange).

In summary, all molecular glues (1–5) induced highly similar Tpx dimer interfaces, with CtFT (4) consistently being the weakest dimerizer. Depending on the respective inhibitor fluorination pattern, our combined NMR and MD analyses revealed local differences in intra- and intermolecular dimer interactions involving the molecular glue itself as well as protein residues surrounding the inhibitor binding site. Since these differences presumably hold the key to understanding the observed differences in induced dimer affinity, we next analyzed molecular glue interactions with the Tpx binding pocket.

### Inhibitor fluorination pattern controls binding pocket engagement

Our MD simulations indicated that intramolecular protein–inhibitor contacts significantly contribute to Tpx dimer stability. However, because inhibitor-bound Tpx WT exists in a monomer–dimer equilibrium, NMR analysis of inhibitor binding to the Tpx monomer is complicated by concurrent dimerization. To nonetheless investigate the effects of molecular glue fluorination on protein binding site engagement, we took advantage of the Tpx W39A mutant, which strongly suppresses dimerization but still covalently binds all inhibitors *via* Tpx residue C40 (Fig. S12 and Table S1).^11,12,24^

Upon assigning the backbone amide resonances of unmodified and inhibitor-bound Tpx W39A (Fig. S13 and S14), we determined the CSP induced by each inhibitor for monomeric Tpx W39A (Fig. 3a). Overall, highly similar interaction profiles emerged, comprising the N-terminal loops of α-helix 1 (active site) and α-helix 2 (containing W70), as well as the C-terminal end of α-helix 3 and the adjacent loop containing residues E107, S108, and I109 (Fig. 3a). This binding pocket matches the intramolecular contacts observed in the inhibitor-bound Tpx dimer crystal structure (6GXG^12^), and was also reproduced in the MD simulations of monomeric Tpx W39A with inhibitors 1–5 (Fig. S15a). Notably, CtFT (4) caused the smallest overall chemical shift changes to monomeric Tpx W39A (Fig. S16), suggesting that the bulky trifluoromethyl group of this molecule reduces contacts between inhibitor and protein.

**Fig. 3:**
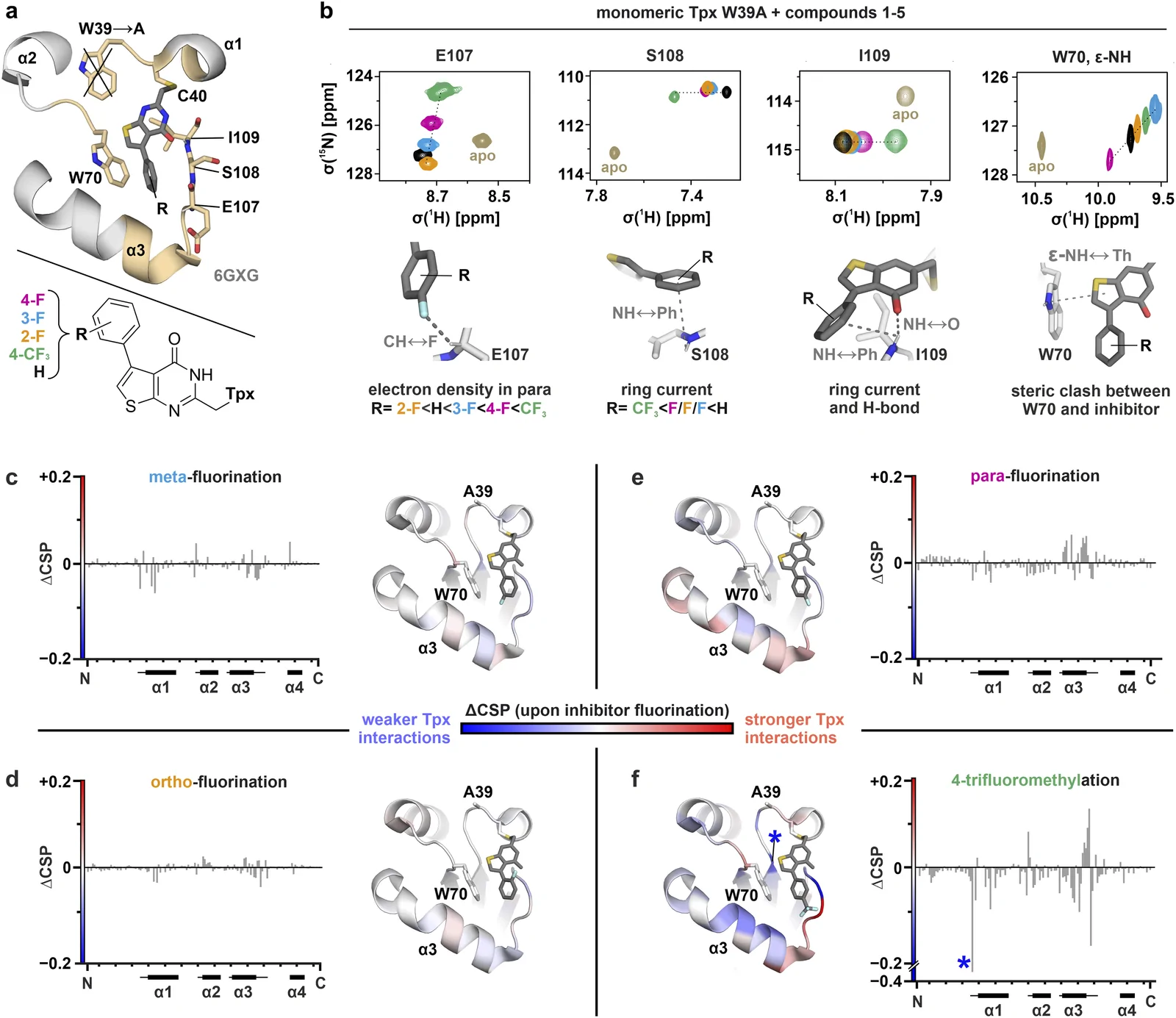
Tpx W39A-bound inhibitors occupy a consensus binding pocket but display fluorination pattern-specific protein interactions. (a) Structural model of monomeric Tpx W39A/inhibitor complexes with the consensus inhibitor binding pocket (sand), derived from the chemical shift perturbation pattern in ^1^H,^15^N-NMR experiments and MD simulations (Fig. S15). Heteroatom color scheme: N: blue; O: red; S: yellow; F: light blue. (b) Amide resonances of selected amino acids reflect interactions with the inhibitors' fluorine, phenyl, oxygen, and thiophene moieties. Hydrogen bonds and aromatic interactions are indicated with dashed lines. (c–f) Pairwise chemical shift perturbation differences (ΔCSP) between non-fluorinated CPT (5) and fluorinated analogs (1–4). Values of 0 indicate no CSP difference between the respective fluorinated inhibitors and the non-fluorinated inhibitor (shown in white on the structure). Values greater or lower than 0 indicate an enhanced or reduced effect on the respective residue due to inhibitor fluorination (shown in red and blue, respectively).

In the ^1^H, ^15^N-NMR spectra of Tpx W39A/inhibitor complexes, key binding site residues W70, E107, S108 and I109 displayed fluorination-dependent chemical shift trajectories which directly reflected the substitution pattern and phenyl ring electron density of bound inhibitors (Fig. 3b and S15). For instance, the E107 backbone amide shifts correlated with electron density at the *para*-position and were thus highest for CtFT (4) and lowest for *ortho*-CFT (3). Likewise, S108 resonances reflected shielding due to phenyl ring electron density, *i.e.* chemical shifts were most pronounced for CPT (5) and least for CtFT (4) but similar for the three monofluorinated derivatives 1–3. In contrast, I109 exhibited the opposite trend, consistent with differential ring-current effects influencing the neighboring S108 and I109 residues (Fig. S15c). Notably, the W70 side-chain NH shifts did not track the inhibitor's phenyl electron density, likely due to inhibitor-induced W70 side-chain reorientation, something we also observed *via* X-ray crystallography (Fig. S15d). Together, these data suggest that all inhibitors adopt a similar pose when bound to monomeric Tpx W39A, and that the inhibitor fluorination pattern fine-tunes interactions with key binding site residues.

To directly pinpoint the consequences of inhibitor fluorination for Tpx binding site engagement, we took advantage of CPT (5), which shares the same scaffold with 1–4 but lacks fluorine substituents. The differences in chemical shift perturbation (ΔCSP) between Tpx resonances of the nonfluorinated inhibitor and the fluorinated derivatives, thus primarily reflect fluorination-dependent changes in inhibitor interactions with the protein surface (Fig. 3c–f and S16).

Fluorination in *ortho*- and *meta*-position had minor effects on the ligand's binding site interaction (Fig. 3c and d), whereas *para*-substitutions strongly affected Tpx residues in α-helix 3 and the succeeding loop containing residues 107–109 (Fig. 3e and f). The trifluoromethyl group in CtFT (4) weakened interactions (negative ΔCSP, blue) with α-helix 3 and residues in the binding pocket, particularly notable for residue S36 (marked with an asterisk in Fig. 3f), suggesting weaker binding site engagement of the inhibitor's thienopyrimidinone moiety. At the same time, stronger interactions with residues in the C-terminal loop of α-helix 3 (positive ΔCSP, red) were observed for this inhibitor (4) agreeing with the presumed location of the electron-dense trifluoromethyl group.

Together, NMR and MD analyses of Tpx W39A/inhibitor complexes thus revealed a conserved binding orientation for all inhibitors except for CtFT (4), which showed a weakly engaged binding mode.

### Fluorination tunes the dynamics of Tpx-bound molecular glues

To investigate the interaction between Tpx and bound inhibitors in solution, we used ^19^F-NMR spectroscopy, taking advantage of the fluorine substituents in compounds 1–4 as sensitive NMR reporters.^25^ Upon binding to Tpx WT or W39A, all inhibitors exhibited ^19^F-chemical shift perturbations and line broadening relative to the free ligands (Fig. 4a and Table S4). As expected, the ^19^F-NMR signals of the dimerizing Tpx WT/inhibitor complexes showed consistently larger linewidths than the ^19^F-NMR signals of monomeric Tpx W39A/inhibitor complexes due to different molecular tumbling speeds. Notably, the ^19^F-NMR signal of *ortho*-CFT (3) was exceptionally broad when bound to Tpx WT but not the W39A mutant, suggesting a particularly strong influence of Tpx residue W39 on *ortho*-CFT (3) dynamics that may arise from the competition with the W39 side chain for the protein binding pocket (see below). Notably, we also observed a high degree of displacement relative to the starting structure for *ortho*-CFT in MD simulations of the monomeric Tpx WT/inhibitor complexes (Fig. 4b, movies 8, 11 and 12). However, the observed broad linewidths for *ortho*-CFT could also reflect exchange between monomeric and dimeric Tpx WT. We therefore compared the linewidths of *ortho*- and *para*-CFT in complex with Tpx WT at similar monomer:dimer ratios by adjusting the protein concentrations according to their respective dimer *K*_D_ values (Fig. 4c). Here, the linewidth for Tpx WT-bound *ortho*-CFT (3) remained larger than for Tpx WT-bound *para*-CFT (1), hinting towards distinct molecular glue dynamics of *ortho*-CFT (3). Since the W70 side chain directly contacts *ortho*-CFT, the inhibitor's exceptionally dynamic behavior is also reflected in the comparatively broad ^1^H, ^15^N-NMR signal of the W70 side chain (see Fig. 2b).

**Fig. 4:**
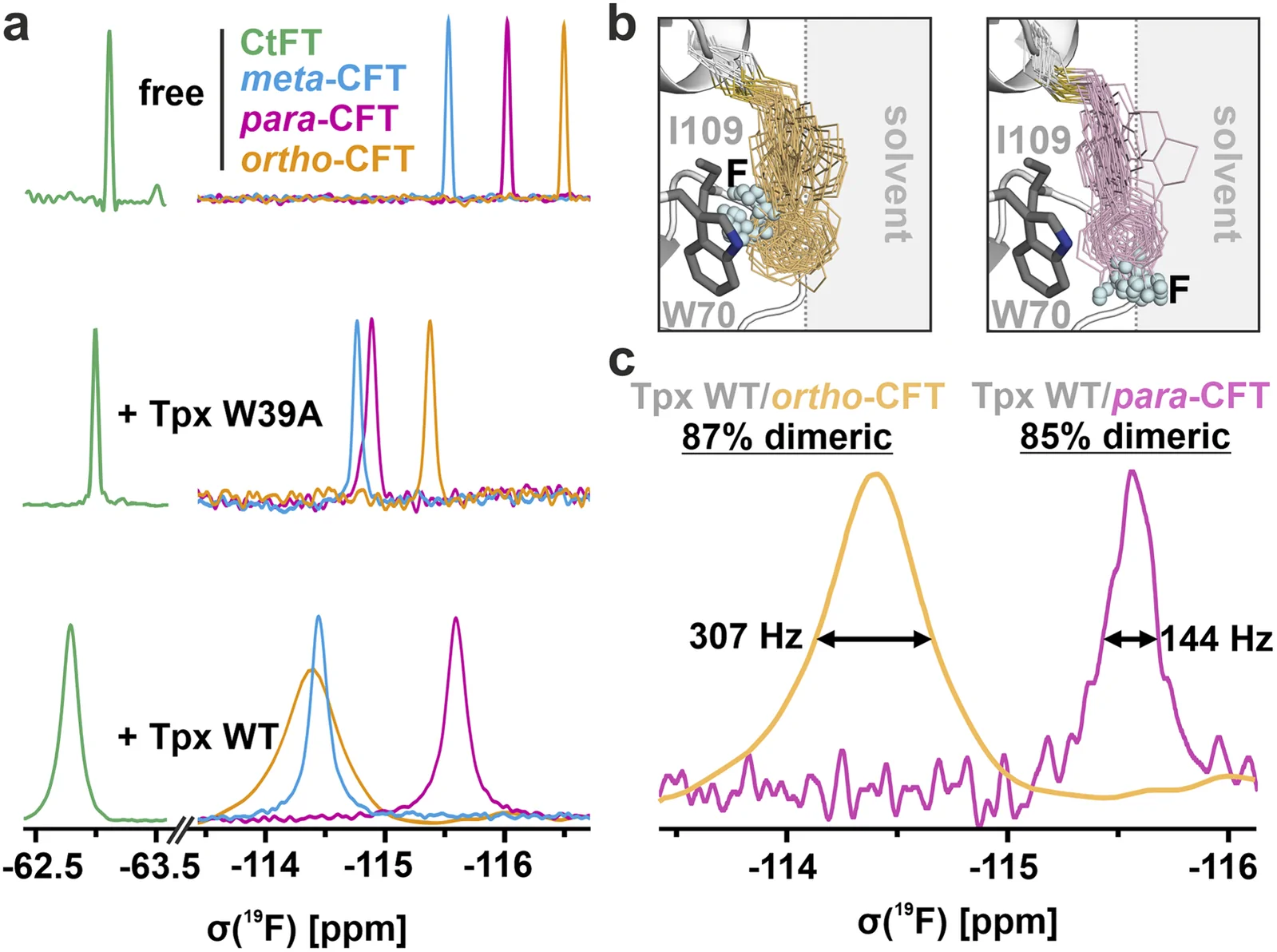
Protein-bound molecular glue dynamics probed by ^19^F-NMR. (a) 1D ^19^F-NMR spectra of fluorinated Tpx inhibitors 1–4 in the free (100 µM analyte), Tpx W39A-bound (100 µM analyte) and Tpx WT-bound states (750 µM analyte to strongly favor dimerization). (b) Tpx WT-bound *para*- and *ortho*-CFT (1, 3) stances from MD simulations (inhibitor orientations in 33 MD frames, evenly sampled over one 100 ns simulation, *ortho*-CFT (3) shown in the “F-in”-conformation). The W70 and I109 side chains in the ligand binding site are shown in grey. The protein–solution boundary surfaces are indicated with dashed lines, and fluorine substituents are shown as light blue spheres. (c) ^19^F-NMR spectra of Tpx WT/*ortho*-CFT and Tpx WT/*para*-CFT at similar monomer:dimer ratios (750 and 100 µM analyte, respectively).

To delineate the effects of dimer formation on the ^19^F-NMR-spectra, we performed dilution series with the Tpx/inhibitor complexes from 750 µM to 50 µM (Fig. S17). Here, as expected, linewidths remained consistently narrower for the dimer-suppressing W39A mutant than the WT protein and in all cases, the extent of the observed ^19^F-NMR signal changes directly reflected the respective dimer affinities.^24^

In summary, the ^19^F-NMR data corroborated the previously determined dimer affinities and indicated W39-dependent *ortho*-CFT dynamics as primary cause for the ligand's limited dimerizer properties compared to the unfluorinated compound CPT (5).

### A tryptophan side chain clashes with covalently bound inhibitors

While the ^1^H, ^15^N-NMR spectroscopic analysis of monomeric Tpx W39A/inhibitor complexes indicated that a “loose” binding pose may underlie the poor dimerizer properties of CtFT (4), structural and dynamic differences between the moderate Tpx dimerizer *ortho*-CFT (3) and the strong dimerizers (1, 2, 5) were less apparent (Fig. 3). Hence, based on our ^19^F-NMR data (Fig. 4), we hypothesized that the W39 side chain might play a non-negligible role in molecular glue positioning and dynamics. Advantageously, the low dimer affinity of Tpx WT/CtFT complexes (*K*_D_ > 400 µM) provided a unique opportunity to directly compare inhibitor-induced CSPs in monomeric CtFT-bound Tpx WT and W39A (Fig. S18). Notably, CtFT (4) showed stronger interactions in the absence of the W39 indole side chain. In line with this, MD simulations of monomeric protein–inhibitor complexes with Tpx WT and Tpx W39A revealed more stable inhibitor binding in the absence of the W39 side chain (Fig. S19g).

To clarify the role of Tpx residue W39 in ligand binding site engagement, we analyzed the indole side-chain orientation in MD simulations of monomeric Tpx WT/inhibitor complexes (movies 8–14). Overall, the W39 side chain populated four states (Fig. 5a): in state I, the W39 indole proton formed a hydrogen bond with the backbone oxygen of residue W70 which occluded the inhibitor binding pocket. In state II, no hydrogen bond was formed, and in states III and IV, the indole sidechain was additionally flipped out of the binding pocket. This was reflected in the W39 *χ*-angles and the distance between the W39 indole proton and the W70 backbone oxygen (defined as *d*_W_).

**Fig. 5:**
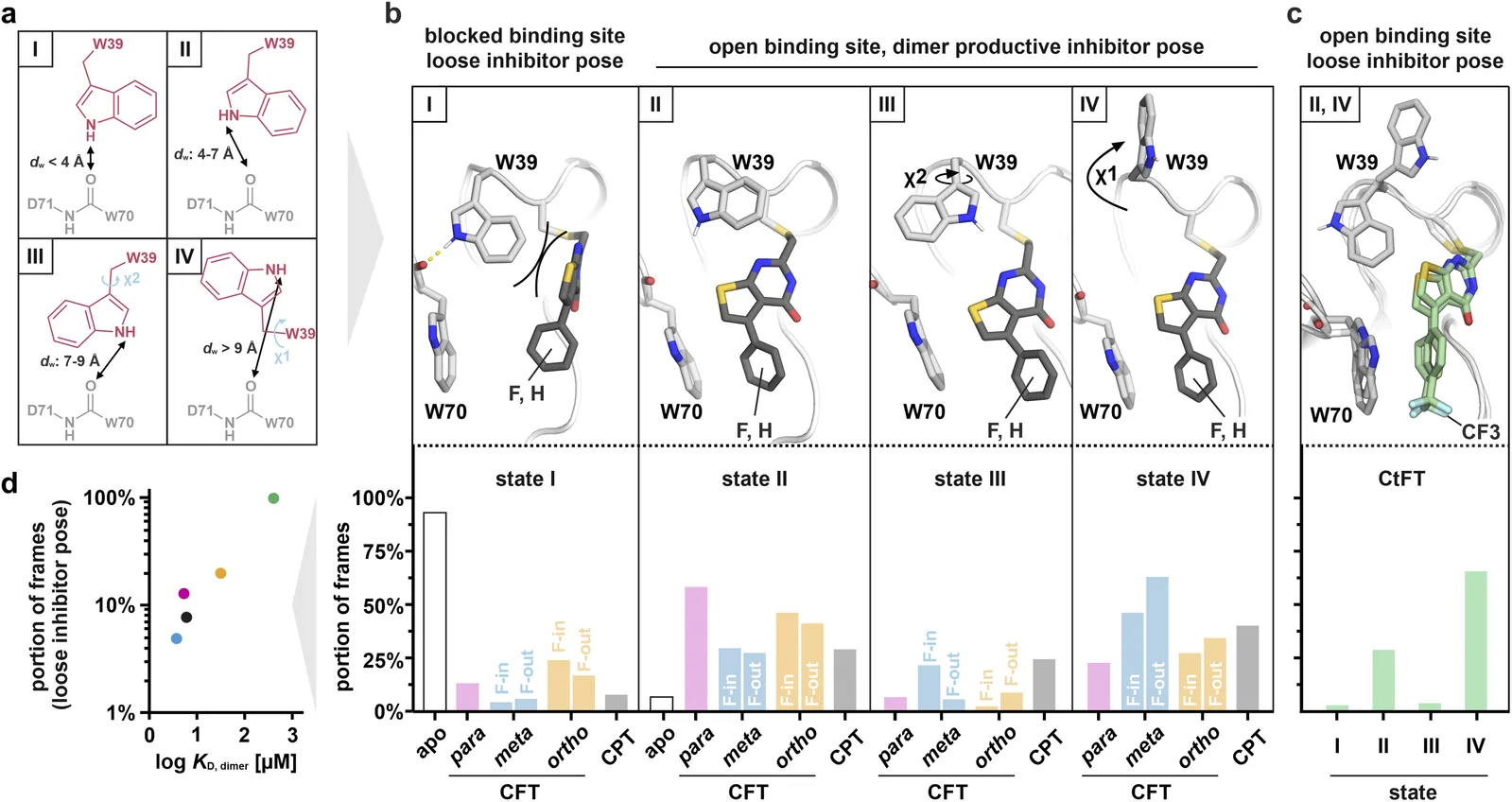
The Tpx W39 side chain is dynamic and modulates molecular glue positioning. (a) W39 displays diverse conformations, clustered into four states (I–IV) based on the distance between the W39 indole NH proton and the W70 carbonyl oxygen (*d*_w_, indicated by double arrows), and the W39 dihedral angles (*χ*_1_ and *χ*_2_). (b) Exemplary conformations of inhibitors and W39, W70 side chains in states I–IV from MD simulations of monomeric Tpx WT/inhibitor complexes (*n* = 5, 100 ns per simulation, 2 fs per frame, movies 8–14). The portion of frames across all repetitions where Tpx is seen to adapt states I–IV is shown as bar graphs. (c) For CtFT (4), only states II and IV were found to be significantly occupied and both result in a “loose” inhibitor binding stance. (d) Inhibitor-induced Tpx dimer affinities from *in vitro* experiments correlate with the portion of MD simulation frames where the respective molecular glue adopts a “loose”, dimer disrupting stance.

To quantify the occupancy of the W39 conformational states, we monitored the hydrogen bond between Tpx residues W39 and W70 *via* the H-bond donor–acceptor distance (*d*_w_) (Fig. 5b and S19, see SI for details). In the absence of inhibitors, 92% of MD frames showed the W39 indole in state I, effectively closing the binding pocket. Spontaneous “opening” of the binding pocket (state II) occurred in only 8% of MD frames, while states III and IV were never observed.

Inhibitor binding induced distinct Tpx W39 conformational preferences that depended on the ligand fluorination pattern (Fig. 5b). *Ortho*-CFT (3) most frequently populated state I, where the W39 side chain occluded the binding pocket and prevented stable ligand positioning, resulting in a “loose” inhibitor stance similar to that observed for CtFT (4, Fig. 5c). However, *ortho*-CFT (3) also sampled states II–IV, enabling inhibitor engagement with the binding pocket. The combination of “loose” (state I) and tightly bound inhibitor stances (states II–IV) coincides with intermediate dimer affinity, suggesting that inhibitor access to the binding pocket, rather than the exact orientation of the W39 side chain, governs dimer affinity (see also Fig. S11). Further supporting this notion, *para*-CFT (1) and CPT (5) induced comparable Tpx dimer affinities, although preferring different W39 orientations, which however, all permitted inhibitor access to the binding pocket. Tpx WT-bound *para*-CFT (1) strongly favored state II, whereas Tpx/CPT complexes showed no clear preference among states II–IV (Fig. 5b). Thus, similar dimer affinities can arise from distinct local W39 dynamics as long as the binding site remains accessible to the molecular glue.

Overall, our MD simulations revealed that the weak dimerizers (3, 4) more frequently adopted “loose” binding poses than the strong dimerizers (1, 2, 5) and that the portion of dimer-productive molecular glue poses correlated with the measured Tpx dimer affinities (Fig. 5d). Together with our NMR analyses, these findings suggest that the W39 side chain competes with bound inhibitors for hydrophobic interactions in the protein binding pocket, resulting in a “molecular quarrel” which controls induced dimer affinity.

## Conclusions

In summary, we have pursued a Fluorine Walk strategy to tune the chemically induced dimer affinity of *T. brucei* Tpx by two orders of magnitude. By combining NMR-spectroscopy and MD simulations, we uncovered the molecular mechanisms that govern chemically induced Tpx dimerization, *i.e.*, the molecular glue binding pose and conformational dynamics at the dimer interface. Systematic fluorination established clear design principles: *para*- and *meta*-substitution of Tpx inhibitors yielded strongly adhering dimerizers that promote stable interface formation, whereas *ortho*-fluorination markedly reduced dimer affinity due to a “molecular quarrel” between the gatekeeping residue W39 and the ligand that restricts productive binding site engagement. Similarly, trifluoromethylation prevented dimer-productive positioning of the molecular glue, thereby greatly reducing Tpx dimer affinity. Since native biomacromolecular complexes span high-, intermediate-, and low-micromolar affinities, tunable, self-assembling CID systems applicable to these demands are needed to complement ultra-high-affinity, quasi-irreversible protein–protein interactions like rapamycin-derived systems.^3,26^ Here, using an integrated methodological approach, we show that fluorination is a valuable tool for the future rational design and optimization of molecular glues across biologically relevant affinity ranges.

## Author contributions

E. S.: protein purification, NMR spectroscopy, analytical SEC, ITC, inhibition kinetics, study design, data analysis, figure design, manuscript writing; J. M. H.: MD simulations; M. D. P.: molecular glue synthesis and characterization; C. G., A. W.: cell assays; S. Z.: DFT calculations; N. B.: SEC-MALS; M. L.: intact-protein mass spectrometry; P. S., H. S. and T. O.: supervision, paper editing. U. A. H.: supervision, funding acquisition, study design, figure design, manuscript writing. All co-authors reviewed the manuscript.

## Conflicts of interest

There are no conflicts to declare.

## Supplementary Material

SC-OLF-D6SC05625C-s001

SC-OLF-D6SC05625C-s002

## Data Availability

All experimental procedures and associated data are provided in the supplementary information (SI). Supplementary information: more references^27^ are cited. See DOI: https://doi.org/10.1039/d6sc05625c. CCDC 2421180–2421182 and 2423222 (2–4) contain the supplementary crystallographic data for this paper.^28*a*–*d*^ The backbone NMR assignment of Tpx W39A was deposited in the BMRB Database under ID: 53259 and can be obtained from DOI: 10.13018/BMR53259. Exemplary molecular dynamics simulation trajectories for this article were rendered as movies and are available on Zenodo at https://doi.org/10.5281/zenodo.22809500.
