# Supplementary material for: Inhibitor fluorination pattern modulates protein surface dynamics and chemically induced dimerization
Source: Chem Sci. 2026 Sep 4;17(40):19160–70. doi: 10.1039/d6sc05625c (PMC13625682; doi:10.1039/d6sc05625c)
Supplement: Supplementary file 1 [file SC-017-D6SC05625C-s001.pdf]

Supplementary Information (SI) for Chemical Science.
This journal is © The Royal Society of Chemistry 2026

**Supporting Information for**

# Inhibitor fluorination pattern modulates protein surface dynamics and chemically induced dimerization

Eric Schwegler,[a] Jean-Martin Harder,[a] Marco D. Preuss,[b] Charlotte Guhl,[a] Shuaibing Zhang,[c] Annika Wagner,[a] Nicole Bader,[d] Pierre Stallforth,[a,c,e] Hermann Schindelin,[d] Till Opatz,[b] Markus Lakemeyer,[a,e] and Ute A. Hellmich*[a,e,f]

[a]Institute for Organic Chemistry and Macromolecular Chemistry, Friedrich Schiller University Jena, 07743 Jena, Germany. E-mail: ute.hellmich@uni-jena.de.

[b]Department of Chemistry, Johannes-Gutenberg University Mainz, 55128 Mainz, Germany.

[c]Department of Paleobiotechnology, Leibniz Institute for Natural Product Research and Infection Biology (HKI), 07745 Jena, German).

[d]Rudolf Virchow Center for Integrative and Translational Bioimaging, Julius-Maximilians-University, 97080 Würzburg, Germany.

[e]Cluster of Excellence Balance of the Microverse, Friedrich Schiller University Jena, 07743 Jena, Germany.

[f]Center for Biomolecular Magnetic Resonance (BMRZ), Goethe-University Frankfurt Max-von-Laue Str. 9, 60438 Frankfurt am Main, Germany.

## Table of Contents for Supporting Information


### A. List of Abbreviations

### B. Material and Methods

a. Materials
b. Molecular Cloning of the Parasitic Oxidoreductase Tryparedoxin (Tpx)
c. Expression and Purification of *T. Brucei* Redox Cascade Components
d. Synthesis and Analysis of Tpx Inhibitors
e. Preparation of Tpx/Inhibitor Complexes and Crosslinked Tpx Dimers
f. Intact-Protein Mass Spectrometry (MS)
g. Trypanosome and HEK293 Cell Survival Assays
h. *In Vitro* Tpx Activity Assay and Inactivation Kinetics
i. *In Vitro* Inhibitor Selectivity Assays
j. Analytical Size Exclusion Chromatography (SEC)
k. Circular Dichroism (CD) Spectroscopy
l. Size Exclusion Chromatography Coupled to Multi-Angle Light Scattering (SEC-MALS)
m. Isothermal Titration Calorimetry (ITC)
n. $^{19}$F-Nuclear Magnetic Resonance ($^{19}$F-NMR) Spectroscopy
o. $^{1}$H, $^{15}$N-NMR Chemical Shift Perturbation Measurements
p. Density Functional Theory (DFT) Calculations
q. Molecular Dynamics (MD) Simulations

### C. Extended Material and Methods

a. Synthesis and Analysis of Tpx inhibitors
b. Analysis of MD Simulations

### D. Supporting Information Tables

Table S1. Covalent modification of Tpx variants by inhibitors 1-5 and crosslinking agent BM(PEG)$_2$.
Table S2. Cytotoxicity of compounds 1-5 against parasitic and human cells.
Table S3. Crystal data and structure refinement for compounds 2-5.
Table S4. $^{19}$F-NMR signal chemical shifts and linewidths of free and Tpx-bound inhibitors 1-4.

### E. Supporting Information Figures

Fig. S1. Tpx constructs used in this study.
Fig. S2. Compounds 1-5 are highly cytotoxic, but selective Tpx inhibitors *in vitro*
Fig. S3. Kinetics of covalent Tpx inhibition are fluorination dependent and inversely correlated with induced dimer affinity.
Fig. S4. Analytical SEC and SEC-MALS of Tpx and Tpx/inhibitor complexes.
Fig. S5. Delayed SEC elution of the Tpx WT/CtFT complex is due to secondary column interactions.
Fig. S6. Dissociation constants ($K_D$) for [Tpx/inhibitor]$_2$ dimers.
Fig. S7. Tpx WT interactions with inhibitors 1-5 monitored by solution NMR spectroscopy.
Fig. S8. Inhibitor interactions and chemically induced dimerization of Tpx WT monitored by $^{1}$H, $^{15}$N-NMR spectroscopy.
Fig. S9. Density functional theory (DFT) calculations and crystal structures of isolated compounds 1-5.
Fig. S10. Phenyl/thiophen dihedral angle distributions of monomeric and dimeric Tpx WT/inhibitor complexes *in silico*.

**Fig. S11. Exemplary frames from MD simulations of dimeric Tpx/inhibitor complexes showing intact and distorted dimer interfaces.**

**Fig. S12. Tpx W39A remains monomeric upon binding of compounds 1-5.**

**Fig. S13. Backbone NMR assignment of Tpx W39A and W39 side chain flexibility.**

**Fig. S14. Chemical shift perturbation of monomeric Tpx W39A upon binding of compounds 1-5.**

**Fig. S15. Structural basis of inhibitor interactions with monomeric Tpx W39A.**

**Fig. S16. A comparison of inhibitor-induced backbone chemical shift perturbations (CSPs) of Tpx W39A reveals fluorination-mediated protein/inhibitor interactions.**

**Fig. S17. The $^{19}$F-NMR signals of free and protein-bound inhibitors provide information about dimer dissociation and bound molecular glue dynamics.**

**Fig. S18. Comparison of NMR chemical shifts of inhibitor-bound Tpx W39A and Tpx WT.**

**Fig. S19. Dynamics and inhibitor interactions with aromatic surface residues W39 and W70.**

**F. Supporting Information References**

## A. List of Abbreviations

| | |
|---|---|
| CD | Circular dichroism |
| CID | Chemically induced dimerization |
| CPT | 2-(chloromethyl)-5-phenylthieno[2,3-*d*]pyrimidin-4(3*H*)-one |
| CtFT | 2-(chloromethyl)-5-(4-(trifluoromethyl)phenyl)thieno[2,3-*d*]pyrimidin-4(3*H*)-one |
| DFT | Density functional theory |
| DMSO | Dimethylsulfoxide |
| HEK | Human embryonic kidney cells |
| ITC | Isothermal titration calorimetry |
| IR | Infrared spectroscopy |
| MALS | Multi-angle light scattering |
| MD | Molecular dynamics |
| *meta*-CFT | 2-(chloromethyl)-5-(3-fluorophenyl)thieno[2,3-*d*]pyrimidin-4(3*H*)-one |
| MS | Mass spectrometry |
| NADPH | Nicotinamide adenine dinucleotide phosphate |
| NiNTA | $Ni^{2+}$-nitrilotriacetic acid |
| NMR | Nuclear magnetic resonance spectroscopy |
| *ortho*-CFT | 2-(chloromethyl)-5-(2-fluorophenyl)thieno[2,3-*d*]pyrimidin-4(3*H*)-one |
| *para*-CFT | 2-(chloromethyl)-5-(4-fluorophenyl)thieno[2,3-d]pyrimidin-4(3*H*)-one |
| PCC | Peroxide clearance cascase |
| PCR | Polymerase chain reaction |
| Px | Peroxidases |
| SEC | Size exclusion chromatography |
| *T. brucei* | *Trypanosoma brucei,* African trypanosomes |
| Tpx | Tryparedoxin |
| TR | Trypanothione reductase |
| $TS_2$/TSH | Trypanothione in the oxidized / reduced state |
| WT | Wild type |

## B. Materials and Methods

### Materials

*T. brucei brucei* 449 Lister 427 parasites and HEK293 cells were generous gifts from Prof. Luise Krauth-Siegel, Heidelberg, and Prof. Thorsten Heinzel, Jena. Ni-NTA beads were purchased from Qiagen. Deuterium oxide ($D_2O$) and $^{15}N$-ammonium chloride ($^{15}NH_4Cl$) were purchased from Eurisotop. U-$^{13}C$-D-glucose was purchased from Cambridge Isotope Laboratories. NADPH was purchased from Roche and the crosslinker 1,8-bismaleimidodiethyleneglycol ($BM(PEG)_2$), L-glutamine, trypsin, and penicillin-streptomycin were purchased from Thermo Fisher Scientific. DMEM and FBS for HEK293 cell culture were purchased from Sigma Aldrich. All other materials were obtained from Carl Roth GmbH unless specified otherwise and were of analytical grade or better.

### Molecular Cloning of the Parasitic Oxidoreductase Tryparedoxin (Tpx)

The plasmid encoding Tpx wildtype (WT) is based on a pETtrx_1b vector and was a generous gift from Prof. Luise Krauth-Siegel, Heidelberg. Expression vectors for the point mutants Tpx W39A, Tpx C40S and Tpx C43S were obtained via Quick change PCR with the KAPA HiFi PCR Kit (Kapa Biosystems). The following primer pairs were used to obtain Tpx W39A ($^{5'}$CTGCCTCCGCGTGCCCCCATGC$^{3'}$ and $^{5'}$GGGGGCACGCGGAGGCAGAAAAG-TAAAG$^{3'}$), Tpx C40S ($^{5'}$CTTTTCTGCCTCCTGGTCCCCCCCATGCC$^{3'}$ and $^{5'}$GTAAAACCC-CGGCATGGGGGGGACCAGGAGGC$^{3'}$); and Tpx C43S ($^{5'}$CTGGTGCCCCCCATCCCGGGG-TTTTACACC$^{3'}$ and $^{5'}$GGTGTAAAACCCCGGGATGGGGGGCACCAG$^{3'}$).

### Expression and Purification of *T. Brucei* Redox Cascade Components

Purified *T. brucei* trypanothione reductase (TR), trypanothione disulfide ($TS_2$), and the plasmid encoding *T. brucei* glutathione peroxidase-type Tryparedoxin peroxidase 3 (Px, pETtrx_1b vector) were generous gifts from Prof. Luise Krauth-Siegel, Heidelberg. TR was stored at a concentration of 1.3 mM at 4°C in 50 mM potassium phosphate buffer pH 7.0 containing 1 mM EDTA, and 1.8 M ammonium sulfate. $TS_2$ was stored at a concentration of 22 mM in water at -20°C. *T. brucei* Tryparedoxin 1 (Tpx) and glutathione peroxidase-type Tryparedoxin peroxidase 3 (Px) were heterologously overexpressed as $His_6$-tagged thioredoxin (Trx) fusion proteins in *E. coli* BL21-Gold(DE3) cells (Agilent) using either 2xYT medium or M9 minimal medium supplemented with $^{15}N$-$NH_4Cl$ (1 g/L) and $^{13}C$-D-glucose (2 g/L) as the sole nitrogen or carbon sources.[1] Cells were lysed and proteins purified as described before:[2] In brief, the Trx-$His_6$-Tpx (or Trx-$His_6$-Px) fusion protein was separated from the lysate via Ni-NTA affinity chromatography and cleaved with His-tagged *tobacco edge virus* (TEV) protease. After dialysis against Tpx buffer (25 mM sodium phosphate buffer pH 7.5, 150 mM NaCl) at 4 °C for 15 h, Tpx (or Px) was separated from the His-tagged Trx cleavage product and the TEV protease via Ni-NTA affinity chromatography and purified by size exclusion chromatography using a HiLoad 16/600 Superdex 75 pg column (Cytiva). Proteins were eluted in Tpx buffer at 4 °C and purity of selected fractions were checked via SDS-PAGE (15% gel) before pooling. Purified proteins were stored at -80 °C.

### Synthesis and Analysis of Tpx Inhibitors

For details on synthesis and analysis, please see Extended Material and Methods. All chemicals were purchased from commercial sources and used without prior purification. Air and humidity sensitive reactions were carried out under argon using the Schlenk technique. Deuterated chloroform was stored over basic aluminum oxide. Solvents for HPLC-MS were purchased from Fisher Scientific in Optima-HPLC-MS® grade. Flash column chromatography was performed using silica gel with an average grain size of 35–70 µm and purchased from Acros Organics. Automated flash column chromatography was carried out using an Isolera Flash Purification System from Biotage with integrated UV-detector. Thin layer chromatography was performed using silica coated aluminum plates from Merck (TLC Silica 60 $F_{254}$).

NMR measurements of small molecules in deuterated solvents purchased from Cambridge Isotope Laboratories were carried out using Avance III HD 300 and Avance III HD 400 spectrometers. Chemical shifts (δ) are reported in parts-per-million (ppm) relative to the residual solvent protons ($^1H$-NMR) or the deuterium coupled $^{13}C$ solvent signal ($^{13}C$-NMR). Coupling constants of protons (J) are reported in Hertz (Hz). Spin multiplicities are reported using the following abbreviations or appropriate combinations of such: s (singlet), d (doublet), t (triplet), m (multiplet). NMR spectra were processed using MestReNova

x64 14.01. (Mestrelab Research S.L.). Electron spray ionization high resolution mass spectrometry (ESI-HRMS) analysis was performed using a 6545 QTof Ultima 3 mass spectrometer with LockSpray-interface at capillary voltages of 2900 V. Electron spray ionization mass spectrometry (ESI) were recorded using a 1200-Series or a 1260-Series Infinity II HPLC-system from Agilent, equipped with a binary pump and integrated diode array detector. The latter was coupled to a LC/MSD-Trap-XTC-mass spectrometer or a LC/MSD Infinitylab LC/MSD (G6125B LC/MSD) from Agilent. An *Ascentis Express* $C_{18}$-column (30 mm x 2.1 mm) with an average particle size of 2.7 µm was used as stationary phase. Fourier-transform infrared (FT-IR) spectra were recorded on a Bruker FT-IR Tensor 27 apparatus and reported in wavenumbers ($\upsilon$). Melting temperatures were measured using a KSP1N from A. Krüss Optronic.

**Preparation of Tpx/Inhibitor Complexes and Crosslinked Tpx Dimers**

100-400 µM purified Tpx in 25 mM sodium phosphate buffer pH 7.5, 150 mM NaCl (Tpx buffer) were incubated with 4 mM tris(2-carboxyethyl)phosphine (TCEP) and a 3-fold excess of inhibitor (4 mM in DMSO) or a 4-fold excess of the crosslinker 1,8-bismaleimidodiethyleneglycol ($BM(PEG)_2$, 10 mM in DMSO) for 30 min at 25 °C. Covalently modified Tpx was purified via size exclusion chromatography with Tpx buffer using a Superdex 75 Increase 10/300 GL column (Cytiva) at 4 °C.

**Intact-Protein Mass Spectrometry (MS)**

20 µM of Tpx WT, C40S and C43S in Tpx buffer were incubated with 400 µM TCEP and 2% DMSO containing no or 80 µM of inhibitors **1**-**5** for 30 min at 25°C. Crosslinked Tpx dimers were generated and purified as described above.

High-resolution intact-protein mass spectrometry was performed on a Dionex Ultimate 3000 HPLC system (Thermo Fisher) coupled to a Bruker maxis HD (after collision cell upgrade) with an electrospray ionization source (end plate offset: 500 V, capillary: 4500 V, nebulizer: 3.0 bar, dry gas: 12.0 L/min, dry temp: 250 °C). Samples were desalted on-line using a Massprep desalting cartridge (Waters) prior to MS-measurement. Internal calibration using sodium formate was performed at the end of each run. Deconvolution of protein spectra of individual peaks was performed using UniDec GUI 6.0.2.[3]

**Trypanosome and HEK293 Cell Survival Assays**

Half maximal effective concentration ($EC_{50}$) values of compounds **1**-**5** against *T. brucei brucei* 449 Lister 427 parasites were determined with an ATPlite Luminescence assay as described previously.[4,5] The $EC_{50}$ was defined as the compound concentration at which a fifty percent decrease in relative luminescence occurred, which indicated that half of the trypanosomes had died. Toxicity of compounds **1-5** in human embryonic kidney (HEK293) was determined with a resazurin assay, which is based on the reduction of resazurin to fluorescent resorufin by living cells.[6] HEK293 cells were maintained in DMEM high glucose supplemented with 10% FBS, 1% penicillin/streptomycin and 1% L-glutamine at 37 °C and 5% $CO_2$. For the toxicity screen, 40000 cells per well were seeded in a 48 well plate (400 µL medium/well) and incubated at 37 °C for 24 hours before treatment with inhibitors **1-5**. Cells were treated with 0.1 µM, 0.3 µM, 0.7 µM, 1 µM, 3 µM, 7 µM, 10 µM, 30 µM, 70 µM and 100 µM of the respective inhibitor, DMSO treatment served as negative control. The overall DMSO concentration was kept at 1 vol.% DMSO per well. After 24 hours of treatment, the medium was exchanged with a medium/resazurin mixture (0.015 mg/mL resazurin per well) and the cells were incubated for 3 h at 37 °C before transferring 100 µl medium from each well into a black opaque-walled 96-well plate. Fluorescence was directly measured using a CLARIOstar Plus reader (BMG Labtech GmbH, Ortenberg, Germany) with a 545-20 nm excitation / 600-40 nm emission filter. Each experiment was done in biological quadruplets and cell viability was analyzed in comparison to the DMSO control by plotting relative fluorescence units (RFU) against inhibitor concentration (Prism, Graphpad). A fifty percent decrease in relative fluorescence indicated the compound concentration where half of the HEK293 cells had died, which was defined as the $EC_{50}$.

***In Vitro* Tpx Activity Assay and Inactivation Kinetics**

To reconstitute the peroxide clearance cascade (PCC), its components (at final concentrations of 360 µM NADPH, 0.1 U/mL TR, 110 µM $TS_2$, 5 µM Tpx, 180 nM Px, 100 µM $H_2O_2$) were mixed in a total volume of 200 µL in a 10 mm quartz cuvette. First, 15 µL NADPH (4.8 mM in TR buffer (40 mM HEPES pH 7.5, 1 mM Na-EDTA), 10 µL TR (2 U/mL in TR buffer), 10 µL $TS_2$ (2.2 mM in sterile filtered, deionized

water), 20 µL Tpx (50 µM in Tpx buffer), 10 µL Px (3.6 µM in Tpx buffer), and 2 µL DMSO were added to 128 µL of Px buffer (100 mM Tris pH 7.5, 150 mM NaCl). The reaction mixture was incubated at 25 °C and absorption at 340 nm was observed with a UVmc2 double-beam UV-Visible spectrophotometer (SAFAS) until the baseline stabilized before starting the reaction with 5 µL $H_2O_2$ (from a 4 mM stock in sterile filtered, deionized water). The absorption at 340 nm was measured for 30 s and fitted linearly (SAFAS SP2000). Of note, this observed PCC activity includes both the enzymatic and non-enzymatic oxidation of NADPH by atmospheric oxygen.

To determine the contribution of the non-enzymatic NADPH oxidation, the reaction mixture was also prepared without Px and measured as described above. Subtracting this value from the observed PCC activity yielded the actual PCC activity, which was set to 100% activity (0% inhibition) for further analyses.

Measuring PCC inhibition (I) at defined time points (t) after the addition of DMSO-dissolved inhibitors **1**-**5** yielded time-resolved inhibition curves. Substoichiometric (2.5 µM, stoichiometry relative to Tpx), equimolar (5 µM) and excess amounts of inhibitor (15 µM, 20 µM, 25 µM, 30 µM for *ortho*-CFT and CtFT; 15 µM, 25 µM, 35 µM, 45 µM for *para*-CFT, *meta*-CFT and CPT) were added to the PCC mixture prior to starting the reaction with $H_2O_2$. For equimolar inhibitor amounts, measurements were carried out as technical triplicates of biological triplicates, in all other cases as technical triplicates. Fitting inhibition (I) at defined time points (t) with **Eq. 1:**

$$I(t) = 100\% * (1 - e^{-kt}) \quad (\mathbf{1})$$

for a given inhibitor concentration yielded $k_{obs}$, which was plotted linearly against inhibitor concentration (shown as X-fold excess relative to Tpx on plots) to calculate second-order rate constants $k_{2nd}$ for compounds **1-5** (OriginPro 2022).[7]

### *In Vitro* Inhibitor Selectivity Assays

To determine the selectivity of the inhibitors in the *in vitro* PCC reaction mix for each enzyme, we modified the PCC assay to leave out individual components. To determine TR inhibition by compounds **1**-**5**, the PCC assay was carried out using only 150 µM NADPH, 10 mU/mL TR, and 100 µM $TS_2$. For the basal TR activity, we added 6.3 µL NADPH (4.8 mM in TR buffer), 10 µL TR (0.2 U/mL in TR buffer), and 2 µL DMSO (or 12 µL to emulate the CtFT conditions, see below) to 172.6 µL TR buffer (or 162.6 µL for the CtFT condition) before starting the reaction with 9.1 µL $TS_2$ (2.2 mM in sterile filtered, deionized water). The absorption at 340 nm was measured for 30 s at 25 °C and fitted with the Safas SP2000 software.

To determine TR inhibition, a final concentration of 4.5 µM inhibitor (3 µM for *ortho*-CFT and CtFT) in 2 µL DMSO (12 µL for CtFT) was added to the reaction mixture and incubated for 5 min at 25 °C before starting the reaction with $TS_2$. TR inhibition was determined in a technical triplicate as described above.

To check Tpx activity, we incubated the PCC in the absence of Px (360 µM NADPH, 0.1 U/mL TR, 110 µM $TS_2$, 5 µM Tpx) with 5 µM of each inhibitor **1**-**5** or DMSO for 25 min (45 min for *meta*-CFT and CPT) at 25 °C before adding 190 nM Px and immediately starting the reaction with 100 µM $H_2O_2$.[5] PCC activity was determined from the absorption decrease at 340 nm as described above in a technical triplicate. The ratio of PCC activity in the presence of inhibitor and the corresponding DMSO control yielded the PCC inhibition due to Tpx binding of inhibitors **1-5**.

To check Px activity, we reduced 25 µM Px with 4 mM TCEP in 100 µL Tpx buffer for 30 min at 25 °C before adding 35 µM inhibitor (500 µM in 7 µL DMSO) or pure DMSO and incubating for another 25 min (45 min for *meta*-CFT and CPT) at 25 °C. The reaction mixture was washed three times with 4 mL Tpx buffer using centrifugal filters with a cut-off of 10 kDa (Merck) to remove TCEP, DMSO, and unbound inhibitor. Px was diluted to 3.6 µM and used in the PCC assay as described above in a technical triplicate.

### Analytical Size Exclusion Chromatography (SEC)

100 µM (or 400 µM) purified Tpx with 4 mM TCEP in 200 µL Tpx buffer were incubated with 2.5 µL, 5 µL, and 15 µL (or 60 µL when 400 µM Tpx were used) inhibitor from a 4 mM stock in DMSO or 15 µL

(60 µL when 400 µM Tpx were used) pure DMSO for 30 min at 25 °C. The reaction mixtures were applied to a Superdex 75 Increase 10/300 GL column (Cytiva) and eluted with Tpx buffer at 4 °C, while recording the absorption at 280 nm. Elution volumes, defined as the peak maxima, were determined with the Chromlab Software (version 6.1, Biorad) in a biological triplicate. 50 µM crosslinked Tpx dimer were eluted as a control.

SEC analyses of 100 µM Tpx WT and Tpx/inhibitor complexes with *para*-CFT (**1**) and CtFT (**4**), as well as 50 µM crosslinked $Tpx_2$, were repeated in the presence of Tpx-buffer with 10% and 30% isopropanol to investigate secondary interactions of Tpx constructs with the SEC column.[8]

**Circular Dichroism (CD) Spectroscopy**

To assess the structural integrity of Tpx with isopropanol, CD spectroscopy was used. Purified Tpx (10 µM) in 2.5 mM $NaP_i$ pH 7.5, 15 mM NaCl was incubated for 30 min at RT in the presence or absence of 30% isopropanol before recording CD spectra (195-260 nm) in a 1 mm quartz cuvette (Hellma) with a J-1500 Circular Dichroism Spectrophotometer (Jasco). As blanks, buffers with no or 30% isopropanol were used, respectively. The ellipticity $\theta$ (in degrees) was measured in a technical triplicate by subtracting the buffer spectra (average of three measurements) from the protein spectra. The mean residue ellipticity ([$\theta$]) was calculated with **Eq. 2**:

$$[\theta]_\lambda = \frac{\mathrm{MRW} \cdot \theta_\lambda}{c \cdot d} \quad (\mathbf{2})$$

where $\theta_\lambda$ is the measured ellipticity (in degrees) at a given wavelength λ (in nm), *c* the protein concentration (in g/mL), and *d* the pathlength (in cm). The MRW is the mean residue weight and was calculated with **Eq. 3:**

$$\mathrm{MRW} = \frac{M_\mathrm{w}}{\mathrm{N} - 1} \quad (\mathbf{3})$$

where $M_w$ is the protein molecular weight (in g/mol) and *N* the total number of protein residues.

**Size Exclusion Chromatography Coupled To Multi-Angle Light Scattering (SEC-MALS)**

To determine the oligomeric state of Tpx/inhibitor complexes by SEC-MALS, 100 µM of purified Tpx/inhibitor complexes were applied to a SEC column (Superdex75 10/300, GE Healthcare) in Tpx buffer (25 mM $NaP_i$ pH 7.5, 150 mM NaCl) at a flow rate of 0.5 ml/min at room temperature. The molecular weights of the eluting proteins were measured using a Dawn Heleos 8+ light scattering detector and an Optilab T-rEX refractive index detector (Wyatt Technologies). Data were analyzed with the ASTRA software (version 6.1.5.22, Wyatt Technologies), assuming a Zimm model.[9]

**Isothermal Titration Calorimetry (ITC)**

Dissociation coefficients ($K_D$) of dimeric Tpx/inhibitor complexes were determined via dilution ITC following our previously established protocol.[4] Purified complexes in Tpx buffer were titrated to Tpx buffer, and the heat signals of each injection was recorded. To observe dimer dissociation, measurements were conducted with 78 µM (Tpx/*para*-CFT), 122 µM (Tpx/*meta*-CFT), 277 µM (Tpx/*ortho*-CFT), 821 µM (Tpx/CtFT), and 176 µM (Tpx/CPT). A MicroCal PEAQ-ITC Automated (Malvern Panalytical) was used to determine the $K_D$ of chemically induced Tpx dimers with compounds **1**, **3** and **5**. After the initial wait time of 2 min, 19 serial injections of 2 µL with a spacing of 3 min where performed. The reference power was set to 11 µcal $sec^{-1}$ and stirring speed to 750 rpm. A MicroCal PEAQ-ITC (Malvern Panalytical) with the same experimental setup was used to determine the $K_D$ value of covalent Tpx WT complexes with *meta*-CFT (**2**, 10 injections), and CtFT (**4**, 20 injections). Each titration was performed in a technical triplicate, and controls included water to water and unmodified Tpx to buffer titrations. Transferability between instruments was checked by repeating the Tpx/*para*-CFT titration. All thermograms were analyzed with the MicroCal PEAQ-ITC analysis software (Malvern Panalytical).

**$^{19}$F-Nuclear Magnetic Resonance ($^{19}$F-NMR) Spectroscopy**

Tpx/inhibitor complexes with fluorinated compounds **1-4** were prepared and purified as described above. Samples were supplemented with 10% $D_2O$ for field-frequency locking. Spectra of 750 μM, 500 μM, 250 μM, 100 μM, and 50 μM inhibitor-bound Tpx WT or Tpx W39A complexes were recorded on a Bruker AVANCE III HD 600 MHz spectrometer equipped with a QCI cryoprobe (Bruker, Rheinstetten) with 512 scans (4096 scans for Tpx WT/*ortho*-CFT complexes due to line broadening) at 298 K. Spectra were processed using TopSpin 4.1 and referenced to trifluoroacetic acid (TFA, signal set to -76.55 ppm).

For free inhibitors, 100 μM of compounds **1-4** were dissolved in Tpx buffer with 10% $D_2O$ and 8% DMSO (13% for CtFT). $^{19}$F-NMR spectra were measured and processed as described above. The effect of DMSO on the fluorine signal was determined via TFA referencing and corrected (-0.16 ppm for 8% DMSO, -0.27 ppm for 13% DMSO).

### $^{1}$H, $^{15}$N-NMR Chemical Shift Perturbation Measurements

$^{15}$N-labelled Tpx WT and Tpx W39A, as well as Tpx/inhibitor complexes with compounds **1**-**5** were prepared as described above and supplemented with 10% $D_2O$ for field-frequency locking, and 0.1 mM 2,2-dimethyl-2-silapentane-5-sulfonic acid (DSS) for hydrogen referencing (DSS signal set to 0 ppm). HSQC spectra were recorded for Tpx WT (150 μM + 4 mM TCEP), Tpx WT/inhibitor complexes (120-180 μM), Tpx W39A (100 μM + 4 mM TCEP) and Tpx W39A/inhibitor complexes (100 μM) at 298 K with a Bruker Avance Neo 800 MHz spectrometer equipped with a TCI cryoprobe (Bruker, Rheinstetten) and processed using TopSpin 4.1. Data analysis was carried out in CcpNMR analysis 3.1.[10] and the inhibitor induced $^{1}$H, $^{15}$N-chemical shift differences Δδ were calculated using **Eq. 4**[11]:

$$\Delta\delta = \sqrt{\Delta\delta_H{}^2 + \left(\frac{\Delta\delta_N}{6.51}\right)^2} \quad (\mathbf{4})$$

where $\Delta\delta_H$ and $\Delta\delta_N$ are the $^{1}$H- and $^{15}$N-chemical shift difference, respectively.

A set of triple resonance assignment experiments (HNCA, HN(CO)CA) implemented in the standard Bruker TopSpin pulse sequences library were recorded for $^{15}$N,$^{13}$C-labelled Tpx WT (300 μM + 4 mM TCEP), Tpx WT/*para*-CFT (180 μM), Tpx WT/CtFT (180 μM), Tpx WT/*ortho*-CFT (350 μM), Tpx W39A (600 μM + 4 mM TCEP) and Tpx W39A/*para*-CFT (200 μM) at 298 K, processed and analyzed as described above to obtain the backbone NMR assignments of the complexes.

### Density Functional Theory (DFT) Calculations

The molecular structures of compounds **1-5** were constructed using ChemBioDraw 22 and initially optimized with the MM2 (Merck Molecular Force Field) method in ChemBioDraw 3D. Further optimization was performed using density functional theory (DFT) at the B3LYP/6-31G(d) level[12], incorporating Grimme's DFT-D3 dispersion correction.[13] To explore conformational flexibility, potential energy surface (PES) scans were conducted by systematically varying the dihedral angle between the thienopyrimidinone core and the phenyl ring of the Tpx inhibitor in 10-degree increments. These calculations were carried out using the M06-2X/def2-TZVP level of theory within the SMD solvation model for water[14], also accounting for Grimme's DFT-D3 dispersion correction. All calculations were performed using the Gaussian 16 program package.[15]

### Molecular Dynamics (MD) Simulations

MD simulations of Tpx/inhibitor complexes (Tpx WT dimer, Tpx WT monomer, and Tpx W39A monomer) were set up using our Tpx/*para*-CFT dimer crystal structure (PDB: 6GXG[4], chain A). For *meta*- and *ortho*-CFT (**2**-**3**), two fluorine orientations, either pointing towards the protein binding pocket or the solvent ("F-in" and "F-out", respectively) were considered. The PyMOL Molecular Graphics System (Version 3.0, Schrödinger, Inc.) was used to prepare and edit structures, and acpype[16] was used to parameterize the cysteine bound inhibitors as dipeptides, adding the parametrization for the respective inhibitor to the amberff14SB[17] gromacs port as residue "CYP". The systems were prepared for MD-simulations by placing the Tpx/inhibitor complexes in a box with 1 nm padding, solvated with TIP3P water[18] and neutralized with sodium ions.

MD simulations were carried out using GROMACS 2021.2.[19] Energy minimization was performed for a maximum of 1000 steps until the maximum force was below 1000 kJ/mol/nm with positional restraints

on the protein, then for a maximum of 2500 steps without restraints. Subsequently, *nVT*- and *npT*-equilibrations for 100 ps followed, setting a temperature of 300 K with the *V-rescale* modified Berendsen thermostat[20] using a time constant of 0.1 ps and a pressure of 1 bar with the Parrinello-Rahman barostat[21] using a time constant of 2 ps. Hydrogen bonds were constrained using the LINCS algorithm.[22] Simulations ran for 100 ns (5x $10^7$ steps of 2 fs). All MD simulations were evaluated using GROMACS (modules *gmx angle*, *gmx pairdist*, and *gmx rms*), Python 3[23] scripts, and *jupyter* notebooks of the Anaconda distribution[24] (modules *matplotlib 3.8.0*[25] and *numpy 1.20.3*[26]). In our analysis of MD simulations, we included all replicates (n = 5 for each set of conditions) and, in the case of dimer simulations, both protein chains. For Tpx complexes with asymmetrically substituted compounds **2** and **3**, we distinguished between "F-in" and "F-out" conformations. Hence, for the analysis of corresponding Tpx dimers with compounds **2** and **3**, we evaluated three different MD simulations with either both inhibitors in the same orientation (both chains "F-in" or both "F-out"), or with mixed conformations (one chain in "F-in", and one chain in "F-out" conformation), and assumed statistical distribution (25% "F-in/F-in", 25% "F-out/F-out", and 50% "F-in/F-out") (see "Analysis of MD simulations" for details).

## C. Extended Material and Methods

### Synthesis and Analysis of Tpx inhibitors

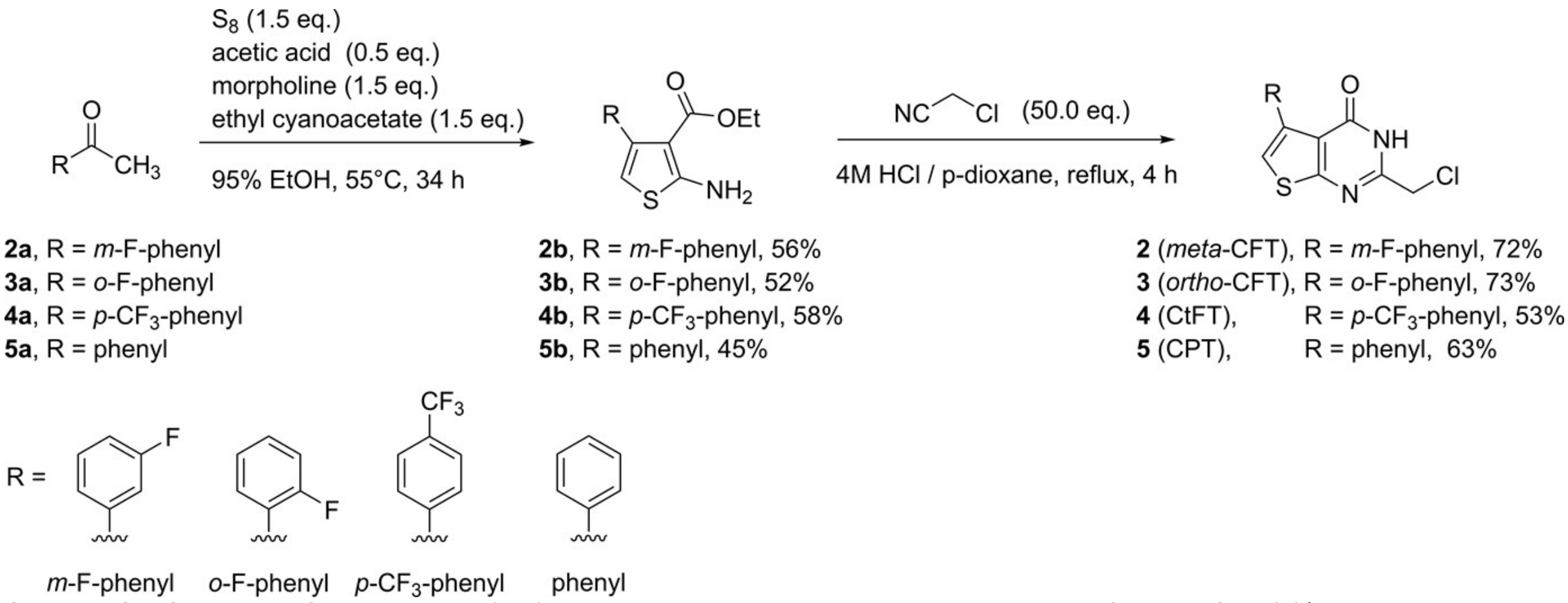


**Scheme S1.** Synthesis of Tpx inhibitors (2-5). Following our previously established protocol for *para*-CFT (1)[4], acetylated aromatic compounds (2a-5a) were converted into thiophens (2b-5b) using a GEWALD-reaction and turned into the final products (2-5) via a condensation with chloroacetonitrile.

### General procedure for the GEWALD-reaction

The reaction procedure was adapted with slight modification from Tormyshev et al.[27] In a screw-cap reaction tube equipped with stirring bar, the respective ketone (3.63 mmol, 1.0 eq.) and ethyl cyanoacetate (5.43 mmol, 1.5 eq.) were dissolved in 0.5 mL 95% ethanol. To the resulting mixture, morpholine (5.43 mmol, 1.5 eq.) and glacial acetic acid (1.81 mmol, 0.5 eq.) were successively added. The reaction mixture was stirred for 4 h at 55 °C before adding three times sulfur (each addition 1.81 mmol, 0.5 eq) over an interval of 10 h. After cooling to room temperature, the reaction mixture was diluted with 50 mL ethyl acetate and extracted three times with 50 mL brine. The organic phase was dried over sodium sulfate, filtered, and concentrated under reduced pressure.

### Ethyl 2-amino-4-(3-fluorophenyl)thiophene-3-carboxylate (2b)

The reaction was performed using 500 mg (3.62 mmol) of 1-(3-fluorophenyl)ethan-1-one **2a**. The crude product was purified using flash column chromatography using 5:1 cyclohexane / ethyl acetate. The product was isolated after recrystallization from 1 mL cyclohexane as yellow crystals (540 mg, 2.04 mmol, 56% Lit.[28]: 53%).

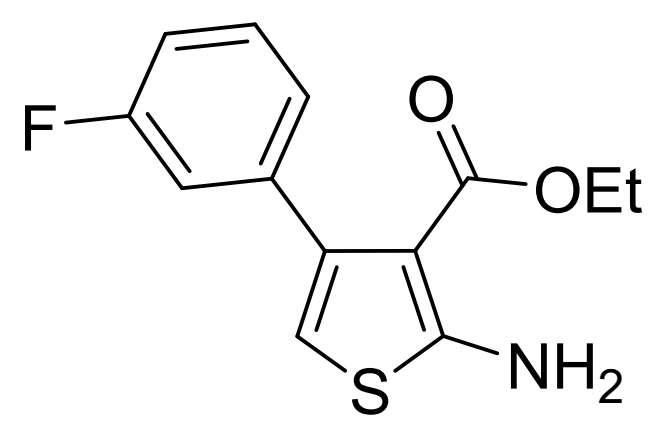


**Melting temperature:** 63.5–67.6 °C (cyclohexane).

**$R_f$:** 0.3 (5:1 cyclohexane / ethyl acetate).

**IR** (ATR): υ ($cm^{-1}$) = 3424, 3311, 1611, 1485, 1318, 1277, 1235, 1128, 1023, 781, 691.

**$^1$H-NMR, COSY (300 MHz, DMSO-*d*$_6$)**: δ/ppm = 7.48 – 7.23 (br, m, 3H, -N*H*$_2$, *H*-5'), 7.18 – 7.01 (m, 3H, *H*-2', *H*-4', *H*-6'), 6.25 (s, 1H, *H*-5), 3.96 (q, *J* = 7.1 Hz, 2H, C*H*$_2$), 0.92 (t, *J* = 7.1 Hz, 3H, C*H*$_3$).

**$^{13}$C-NMR, HSQC, HMBC (75 MHz, DMSO-*d*$_6$)**: δ/ppm = 165.3 (*C*$_q$-2), 164.5 (-*C*$_q$OO-), 161.4 (d, $^1J$ (C-F) = 242.1 Hz, *C*$_q$-3'), 140.6 (d, $^3J$ (C-F) = 8.5 Hz, *C*$_q$-1'), 139.0 (d, $^4J$ (C-F) = 2.2 Hz, *C*$_q$-4), 129.0 (d, $^3J$ (C-F) = 8.6 Hz, *C*-5'), 124.8 (d, $^4J$ (C-F) = 2.6 Hz, *C*-6'), 115.5 (d, $^2J$ (C-F) = 21.6 Hz, *C*-4'), 113.2 (d, $^2J$ (C-F) = 20.8 Hz, *C*-2'), 105.9 (*C*-5), 102.6 (*C*$_q$-3), 58.7 (*C*H$_2$), 13.7 (*C*H$_3$).

**$^{19}$F-NMR (282 MHz, DMSO-*d*$_6$)**: δ/ppm = -115.7 (ddd, *J* = 10.8, 9.0, 6.0 Hz).

**ESI-HRMS:** calculated for $[C_{13}H_{12}FNO_2S+H]^+$ m/z = 266.0646, found: m/z = 266.0653.

The analytical data is in accordance with literature.[28]

**Ethyl 2-amino-4-(2-fluorophenyl)thiophene-3-carboxylate (3b)**

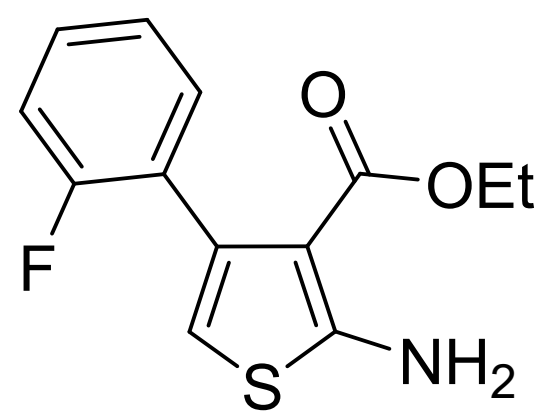

The reaction was performed using 500 mg (3.62 mmol) of 1-(2-fluorophenyl)ethan-1-one **3a**. The crude product was purified using flash column chromatography using 5:1 cyclohexane / ethyl acetate and the pure product was isolated as yellow crystals (500 mg 1.88 mmol, 52%, Lit.[28]: 35%).

**Melting temperature:** 99.7–100.2 °C.

**$R_f$:** 0.3 (5:1 cyclohexane / ethyl acetate).

**IR** (ATR): υ (cm$^{-1}$): 3426, 3314, 1580, 1486, 1470, 1321, 1280, 1225, 1136, 1097, 783, 760.
**$^1$H-NMR, COSY (300 MHz, DMSO-*d*$_6$)**: δ/ppm = 7.43 – 7.22 (m, 3H, *H*-Phenyl, -N*H*$_2$), 7.20 – 7.06 (m, 2H, *H*-Phenyl, *H*-3'), 6.27 (s, 1H, *H*-5), 3.91 (q, *J* = 7.1 Hz, 2H, C*H*$_2$), 0.85 (t, *J* = 7.1 Hz, 3H, C*H*$_3$).*

**$^{13}$C-NMR, HSQC, HMBC (75 MHz, DMSO-*d*$_6$)**: δ/ppm = 164.6 (-$C_q$OO-), 164.5 ($C_q$-2), 159.6 (d, $^1J$ (C-F) = 244.2 Hz, $C_q$-2'), 133.6 ($C_q$-4), 130.6 (d, *J* (C-F) = 3.8 Hz, *C*-Phenyl), 128.9 (d, *J* = 8.2 Hz, *C*-Phenyl), 126.5 (d, $^2J$ (C-F)= 16.0 Hz, $C_q$-1'), 123.7 (d, *J* (C-F) = 3.3 Hz), 114.6 (d, $^2J$ (C-F) = 22.2 Hz, *C*-3'), 106.3 (*C*-5), 103.5 ($C_q$-3), 58.6 (*C*H$_2$), 13.6 (*C*H$_3$).*

**$^{19}$F-NMR (282 MHz, DMSO-*d*$_6$)**: δ/ppm = -114.8 (ddd, *J* = 10.6, 7.5, 5.1 Hz).

**ESI-HRMS:** calculated for $[C_{13}H_{12}FNO_2S+H]^+$ m/z = 266.0646, found: m/z = 266.0649.

*A full assignment of all $^1$H-NMR and $^{13}$C-NMR signals was not possible. The respective signals were indicated as *H*-Phenyl and *C*-Phenyl.

The analytical data is in accordance with literature.[28]

**Ethyl 2-amino-4-(4-(trifluoromethyl)phenyl)thiophene-3-carboxylate (4b)**

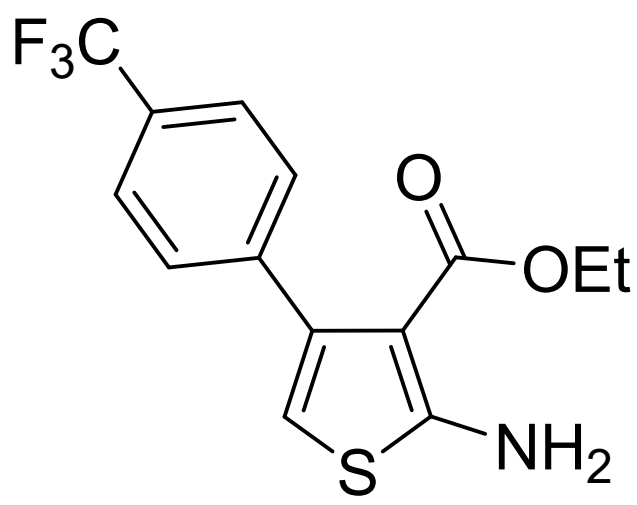

The reaction was performed using 2000 mg (10.63 mmol) of 1-(4-(trifluoromethyl)phenyl)ethan-1-one **4a**. The crude product was purified using flash column chromatography using 5:1 cyclohexane / ethyl acetate. The product was isolated after recrystallization from 4 mL cyclohexane as yellow crystals (1942 mg, 6.16 mmol, 58%, Lit.[28]: 60%).

**Melting temperature:** 102.6–102.9 °C.

**$R_f$:** 0.3 (5:1 cyclohexane / ethyl acetate).

**IR** (ATR): υ (cm$^{-1}$) = 1639, 1594, 1321, 1271, 1104, 1066, 1019, 784.

**$^1$H-NMR, COSY (300 MHz, Chloroform-*d*)**: δ/ppm = 7.63 – 7.53 (m, 2H, *H*-3', *H*-5'), 7.47 – 7.34 (m, 2H, *H*-2', *H*-6'), 6.20 – 6.04 (m, 3H, -N*H*$_2$, *H*-5), 4.04 (q, *J* = 7.1 Hz, 2H, C*H*$_2$), 0.92 (t, *J* = 7.2 Hz, 3H, C*H*$_3$).

**$^{13}$C-NMR, HSQC, HMBC (75 MHz, Chloroform-*d*)**: δ/ppm = 165.5 (-$C_q$OO-), 164.2 ($C_q$-2), 142.3 ($C_q$-1'), 140.3 ($C_q$-3), 129.4 (*C*-2', *C*-6'), 129.1 (q, $^2J$(C-F) = 32.3 Hz, $C_q$-4'), 124.5 ppm (q, $^1J$(C-F)=271.9 Hz, $C_q$F$_3$), 124.3 (q, $^3J$(C-F) = 3.8 Hz, *C*-3', *C*-5'), 106.4 (*C*-5), 105.9 ($C_q$-3), 59.8 (*C*H$_2$), 13.8 (*C*H$_3$).

**$^{19}$F-NMR (282 MHz, Chloroform-*d*)**: δ/ppm = -62.4 (s).

**ESI-HRMS:** calculated for $[C_{14}H_{12}F_3NO_2S+H]^+$ m/z = 316.0614, found: m/z = 316.0616.

The analytical data is in accordance with literature.[28,29]

### Ethyl 2-amino-4-phenylthiophene-3-carboxylate (5b)

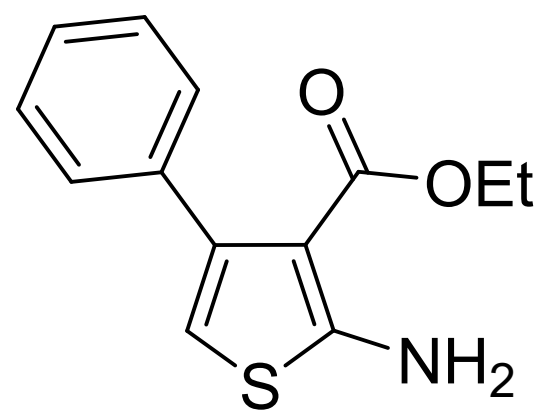


The reaction was performed using 400 mg (3.33 mmol) of acetophenone **5a**. The crude product was purified using flash column chromatography using 5:1 cyclohexane / ethyl acetate. The product was isolated as orange crystals (373 mg, 1.15 mmol, 45%, Lit.[27]: 64%).

**Melting temperature:** 94.0–95.1 °C, (Lit.)[30]: 97–99 °C.

**$R_f$:** 0.3 (5:1 cyclohexane / ethyl acetate).

**FT-IR** (ATR): υ ($cm^{-1}$) = 3424, 3311, 1650, 1636, 1580, 1488, 1318, 1279, 1134, 1024, 731, 696.

**$^1$H-NMR, COSY (300 MHz, DMSO-$d_6$)**: δ/ppm = 7.47 – 7.11 (m, 7H, -N$H_2$, *H*-2', *H*-3', *H*-4', *H*-5', *H*-6'), 6.16 (s, 1H, *H*-5), 3.94 (q, *J* = 7.1 Hz, 2H, C$H_2$), 0.88 (t, *J* = 7.1 Hz, 3H, C$H_3$).

**$^{13}$C-NMR, HSQC, HMBC (75 MHz, DMSO-$d_6$)**: δ/ppm = 165.1 ($C_q$-2),164.7 (-$C_q$OO-), 140.5 ($C_q$-4), 138.3 ($C_q$-1'), 128.6 (*C*-3', *C*-5'), 127.2 (*C*-2', *C*-6'), 126.5 (*C*-4'), 105.1 (*C*-5), 102.9 ($C_q$-3), 58.6 ($CH_2$), 13.7 ($CH_3$).

**ESI-MS:** m/z (%) = 248.1 (100) $[M+H]^+$.

The analytical data is in accordance with literature.[27,30]

### General procedure for the pyrimidinone condensation starting from 2-amino-thiophene-3-carboxylates

The reaction procedure was adapted with slight modification from Klein et al.[4]

In a 50 mL round bottom flask equipped with stirring bar and reflux condenser, a 4 M HCl / p-dioxane mixture was prepared by slow addition of 4.3 mL of methanol (0.11 mol) to an ice-cooled mixture of acetyl chloride (0.11 mol) and 25 mL p-dioxane. The respective 2-amino-thiophene-3-carboxylate (1.00 mmol, 1 eq.) and chloroacetonitrile (25 mmol, 25 eq.) were added to the acidic solution and heated to reflux for 4 h. After cooling to room temperature, the mixture was diluted with 50 mL of water and extracted three times with 50 mL ethyl acetate. The organic phase was washed once with 50 mL saturated sodium hydrogen carbonate (aq.) solution and 50 mL brine, dried over sodium sulfate and concentrated under reduced pressure.

### 2-(Chloromethyl)-5-(3-fluorophenyl)thieno[2,3-*d*]pyrimidin-4(3*H*)-one (*meta*-CFT, 2)

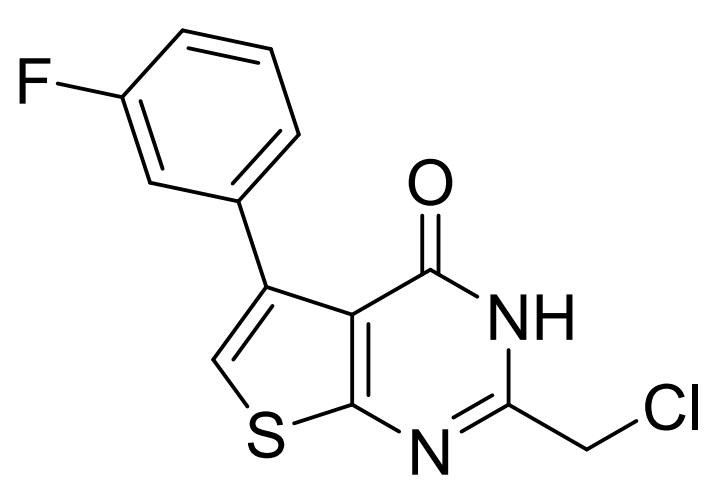


The reaction was performed using 400 mg (1.51 mmol) of compound **2b**. The crude product was purified using flash column chromatography using a 9:1 to 4:1 gradient of cyclohexane / ethyl acetate. The product was isolated as colorless crystals (320 mg, 1.09 mmol, 72%).

**Melting temperature:** Decomposition > 237.3 °C.

**$R_f$:** 0.3 (2:1 cyclohexane / ethyl acetate).

**IR** (ATR): υ ($cm^{-1}$) = 1651, 1589, 1220, 1171, 1050, 753, 719, 691, 666, 644, 610.

**$^{1}$H-NMR, COSY (300 MHz, DMSO-*d*$_6$)**: δ/ppm = 12.82 (s, 1H, N*H*), 7.66 (s, 1H, *H*-6), 7.51 – 7.32 (m, 3H, *H*-4', *H*-5', *H*-6'), 7.29 – 7.09 (m, 1H, *H*-2'), 4.60 (s, 2H, C$H_2$).

**$^{13}$C-NMR, HSQC, HMBC (75 MHz, DMSO-*d*$_6$)**: δ/ppm = 165.3 ($C_q$-7a), 161.5 (d, $^{1}J$ (C-F) = 242.2 Hz, $C_q$-3'), 157.8 ($C_q$-4), 153.2 ($C_q$-2), 137.2 (d, $^{3}J$ (C-F) = 8.6 Hz,

$C_q$-1'), 137.0 (d, $^{4}J$ (C-F) = 2.3 Hz, $C_q$-5), 129.5 (d, $^{3}J$ (C-F) = 8.5 Hz, *C*-5'), 125.4 (d, $^{4}J$ (C-F) = 2.7 Hz, *C*-6'), 122.6 (*C*-6), 119.8 ($C_q$-4a), 116.4 (d, $^{2}J$ (C-F) = 22.4 Hz, *C*-4'), 114.2 (d, $^{2}J$ (C-F) = 20.9 Hz, *C*-2'), 42.4 ($CH_2$).

**$^{19}$F-NMR (282 MHz, DMSO-*d*$_6$)**: δ/ppm = -114.9 (ddd, *J* = 11.6, 9.2, 5.7 Hz).

**ESI-HRMS:** calculated for $[C_{13}H_8ClFN_2OS+H]^+$ m/z = 295.0103, found: m/z = 295.0114.

### 2-(Chloromethyl)-5-(2-fluorophenyl)thieno[2,3-*d*]pyrimidin-4(3*H*)-one (*ortho*-CFT, 3)

The reaction was performed using 350 mg (1.32 mmol) of compound **3b**. The crude product was purified using flash column chromatography using a 9:1 to 4:1 gradient of cyclohexane / ethyl acetate. The product was isolated as colorless crystals (285 mg, 0.97 mmol, 73%).

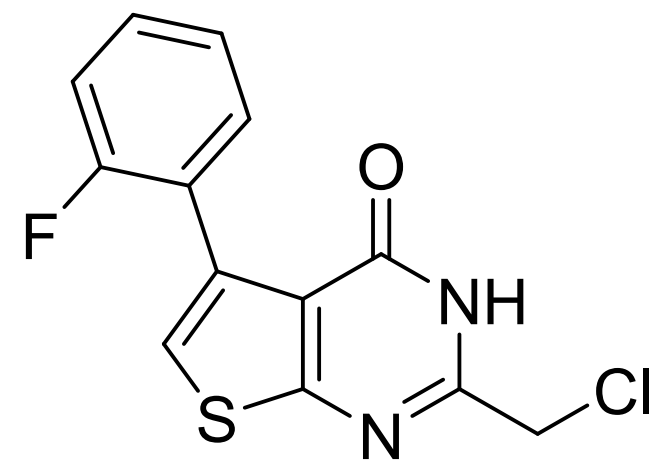


**Melting temperature:** Decomposition > 238.5 °C.

**R$_f$:** 0.3 (2:1 cyclohexane / ethyl acetate).

**IR** (ATR): υ ($cm^{-1}$) = 1651, 1589, 1219, 1171, 1050, 753, 719, 691, 666, 644, 610.

**$^{1}$H-NMR, COSY (300 MHz, DMSO-*d*$_6$)**: δ/ppm = 12.77 (s, 1H, N*H*), 7.60 (s, 1H, *H*-6), 7.53 – 7.37 (m, 2H, *H*-Phenyl), 7.36 – 7.16 (m, 2H, *H*-Phenyl), 4.59 (s, 2H, C$H_2$).*

(*A full assignment of all $^{1}$H-NMR and $^{13}$C-NMR signals was not possible. The respective signals were indicated as *H*-Phenyl and *C*-Phenyl.)

**$^{13}$C-NMR, HSQC, HMBC (75 MHz, DMSO-*d*$_6$)**: δ/ppm = 164.3 ($C_q$-7a), 159.7 (d, $^{1}J$ (C-F) = 245.8 Hz, $C_q$-2'), 157.5 ($C_q$-4), 153.3 ($C_q$-2), 131.6 (d, *J* (C-F) = 3.0 Hz, *C*-Phenyl), 131.2 ($C_q$-5), 129.9 (d, *J* = 8.3 Hz, *C*-Phenyl), 123.9 (d, *J* = 3.6 Hz, *C*-Phenyl), 123.4 (d, *J* = 15.5 Hz, $C_q$-1'), 123.1 (*C*-6), 121.1 ($C_q$-4a), 115.1 (d, *J* = 21.9 Hz, *C*-3'), 42.5 ($CH_2$).

**$^{19}$F-NMR (282 MHz, DMSO-*d*$_6$)**: δ/ppm = -114.8 – -114.9 (m).

**ESI-HRMS:** calculated for $[C_{13}H_8ClFN_2OS+H]^+$ m/z = 295.0103, found: m/z = 295.0106.

### 2-(Chloromethyl)-5-(4-(trifluoromethyl)phenyl)thieno[2,3-*d*]pyrimidin-4(3*H*)-one (CtFT, 4)

The reaction was performed with 220 mg (0.63 mmol) of compound **6**. The crude product was purified using flash column chromatography using 2:1 cyclohexane / ethyl acetate. The pure product was isolated as brown crystals after recrystallization from 5 mL ethanol (114 mg, 0.33 mmol, 53%).

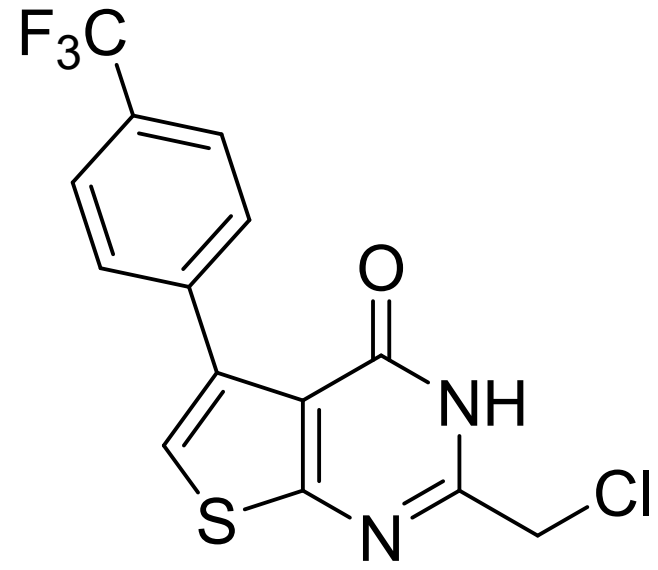


**Melting temperature:** Decomposition > 244 °C.

**R$_f$:** 0.5 (2:1 cyclohexane / ethyl acetate).

**IR** (ATR): υ ($cm^{-1}$) = 1653, 1597, 1318, 1176, 1128, 1064, 853, 772, 597, 431.

**$^{1}$H-NMR, COSY (400 MHz, DMSO-$d_6$)**: δ/ppm = 12.88 (s, 1H, N*H*), 8.07 – 7.41 (m, 5H, *H*-2', *H*-3', *H*-5', *H*-6', *H*-6), 4.61 (s, 2H, C*H*$_2$).

**$^{13}$C-NMR, HSQC, HMBC (101 MHz, DMSO-$d_6$)**: δ/ppm = 165.4 ($C_q$-7a), 157.9 ($C_q$-4), 153.4 ($C_q$-2), 139.1 ($C_q$-1'), 136.8 ($C_q$-5), 130.1 (*C*-2', *C*-6'), 127.8 (q, $^2J$ (C-F) = 31.8 Hz, $C_q$-4'), 124.5 (q, $^3J$ (C-F) = 3.7 Hz, *C*-3', *C*-5'), 124.4 (q, $^1J$ (C-F) = 272.1 Hz, $C_q$F$_3$), 123.3 (*C*-6), 119.8 ($C_q$-4a), 42.4 ( *C*H$_2$).

**$^{19}$F-NMR (282 MHz, DMSO-$d_6$)**: δ/ppm = -61.4 (s).

**ESI-HRMS:** calculated for $[C_{14}H_8ClF_3N_2OS+H]^+$ m/z = 345.0071, found: m/z = 345.0076.

### 2-(Chloromethyl)-5-phenylthieno[2,3-*d*]pyrimidin-4(3*H*)-one (CPT, 5)

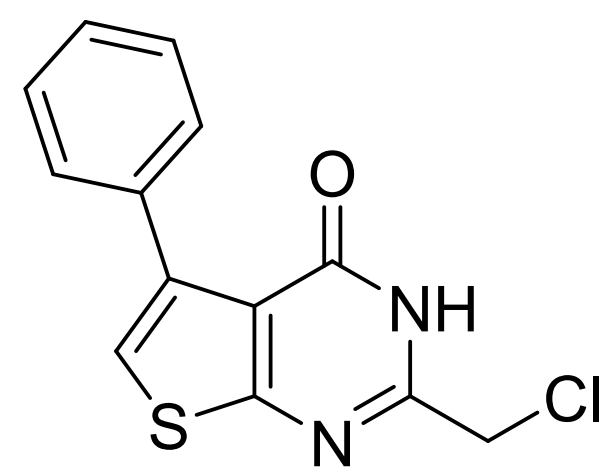

The reaction was performed using 280 mg (1.13 mmol) of compound **5**. The crude product was purified using flash column chromatography using a 6:1 to 2:1 gradient of cyclohexane / ethyl acetate. The pure product was isolated as colorless crystals after recrystallization from 21 mL ethanol (190 mg, 0.71 mmol, 63%).

**Melting temperature:** Decomposition > 227 °C.

**R$_f$:** 0.3 (2:1 cyclohexane / ethyl acetate).

**IR** (ATR): υ (cm$^{-1}$) = 2839, 1644, 1592, 752, 722, 693, 665, 643, 607.

**$^{1}$H-NMR, COSY (300 MHz, DMSO-$d_6$)**: δ/ppm = 12.78 (s, 1H, N*H*), 7.63 – 7.50 (m, 3H, *H*-3', *H*-5', *H*-6), 7.46 – 7.33 (m, 3H, *H*-2', *H*-4', *H*-6'), 4.61 (s, 2H, C*H*$_2$).

**$^{13}$C-NMR, HSQC, HMBC (75 MHz, DMSO-$d_6$)**: δ/ppm = 165.3 ($C_q$-7a), 157.8 ($C_q$-4), 153.1 ($C_q$-2), 138.5 ($C_q$-1'), 135.1 ($C_q$-5), 129.4 (*C*-3', *C*-5'), 127.6 (*C*-2', *C*-6'), 127.4 (*C*-4'), 121.7 (*C*-6), 119.9 ($C_q$-4a), 42.5 (*C*H$_2$).

**ESI-HRMS:** calculated for $[C_{13}H_9ClN_2OS+H]^+$ m/z = 277.1097, found: m/z = 277.0198.

### Analysis of MD simulations

#### Dimer interface contacts (i-iii, for Fig. 2 and iv for Fig. S11)

To analyze Tpx WT/inhibitor dimer dissociation and intramolecular protein/inhibitor contacts, we quantified MD simulation frames where critical distances (i-iv) between inter- and intramolecular interaction sites exceeded defined thresholds: (i) inhibitor stacking was monitored via the distance between one inhibitor's thiophene sulfur to the other inhibitor's pyrimidinone centroid ($d_{S\text{-}PM'}$, threshold: 4 Å based on crystal structures of the Tpx/*para*-CFT dimer and isolated compounds **1-5**). The distance was evaluated using the GROMACS module *gmx pairdist* with the sulfur atom as *reference* and the pyrimidone ring atoms as *selection* using the *-seltype* keyword *part_res_cog*; (ii) the inter-chain salt bridge between K102 and E107' was monitored via the distance between the C$_α$-atoms of both residues to prevent contributions from side chain flexibility ($d_{Cα\text{-}Cα'}$, threshold: 11 Å based on the Tpx/*para*-CFT dimer structure) and evaluated with *gmx pairdist* using the C$_α$-atoms as *reference* and *selection,* respectively; (iii) inhibitor displacement was monitored via the distance between the NH-proton of I109 and the inhibitor's oxygen ($d_{NH\text{-}O}$, threshold: 3 Å, based on the typical length of a strong hydrogen bond) and evaluated with *gmx pairdist* with the atoms as *selection* and *reference*; and (iv) W39 displacement was monitored via the distance between the W39 indole NH-proton and the carbonyl oxygen of W70 ($d_W$, threshold 9 Å) and evaluated with *gmx pairdist*, accordingly (equal to tryptophan state IV in monomeric Tpx/inhibitor complexes, see below for details).

**Inhibitor interactions with Tpx W39A (for Fig. S15)**

To analyze the inhibitor orientation in Tpx W39A/inhibitor complexes, the mean distances between protein and small molecule at four interaction sites (backbones of residues E107, S108, I109, as well as the W70 side chain) were determined for each MD simulation and averaged (n= 5). For the interaction with E107, the distance between the residue's $C_\alpha$ proton and the inhibitor's fluorine atom(s) was determined ($d_{CH\text{-}F}$) using *gmx pairdist* with the proton as *reference* and the fluorine(s) as selection. For CtFT, the distance to the closest fluorine was evaluated. To check the positioning of the inhibitor's phenyl moiety, the distances between the phenyl ring centroid (obtained from the command *-seltype part_res_cog* on the ring carbon atoms) and the respective backbone NH proton of Tpx residues S108 and I109 ($d_{NH\text{-}Ph}$) were determined with *gmx pairdist*. An additional hydrogen bond between the I109 backbone NH-proton and the inhibitor's oxygen ($d_{NH\text{-}O}$) was monitored via the distance between these atoms and evaluated with *gmx pairdist*. To investigate inhibitor interactions with the W70 side chain, we also evaluated the distance between the inhibitor's thiophene ring centroid (obtained from the command -*seltype part_res_cog* on the ring carbon atoms) and the W70 side chain NH-proton ($d_{NH\text{-}Th}$) with *gmx pairdist*.

**Inhibitor RMSDs of monomeric Tpx/inhibitor complexes (for Fig. S18)**

To analyze the inhibitor's conformational ensemble in monomeric complexes with Tpx WT and Tpx W39A, we aligned the MD frames of each simulation to the heavy atoms of residue C40 and the W70 backbone and calculated the RMSD (all heavy atoms of the inhibitor) to the same inhibitor in the pose obtained from our Tpx/*para*-CFT dimer structure (6GXG, chain A) using *gmx rms* with the respective index groups. RMSDs from five independent MD simulations were averaged for each inhibitor (and, if need be, in “F-in” and “F-out” conformation, respectively).

**Definition and evaluation of tryptophan states (I-IV, for Fig. 5 and Fig. S18)**

To analyze the interplay between Tpx-bound inhibitors **1-5** and residue W39, we defined four W-states (I-IV) based on observations from our MD-simulations and structural data from the literature.[4,31–35] As a proxy for the W39 side chain orientation, we chose to monitor the distance between the W39 indole NH-proton and the backbone oxygen of Tpx residue W70 ($d_w$) with *gmx pairdist*. We defined W-state I by an intact hydrogen bond between the W39 side chain and the W70 backbone which was assumed for a $d_w$ < 4 Å. This state is present in the Tpx/*para*-CFT crystal structure (6GXG, chain C[4]), the Alpha Fold2 prediction for *T. brucei* Tpx WT in the apo state (AF-O77404-F1-v4[31,32]), and crystal structures of orthologous proteins (*L. major* Tpx[33], *C. fasciculata* Tpx[34], see **Fig. S13**). A disruption of this hydrogen bond was termed state II and assumed for a $d_w$ of 4-7 Å, which for instance can be seen in the crystal structure of the Tpx/*para*-CFT dimer interface (6GXG, chains A, B[4]). In our MD simulations, we also observed the rare event of a W39 side chain flip (χ2-axis), which led to the formation of a hydrogen bond with the pyrimidinone moiety of inhibitors **1-5**, and was observed for a $d_w$ of 7-9 Å. A larger distance $d_w$ (< 9 Å) indicated full solvent exposure of the W39 side chain through rotation (χ1-axis), and was termed state IV, which can be seen in the *T. brucei* Tpx WT apo crystal structure (1O73[35]).

## D. Supporting Information Tables

**Table S1.** Covalent modification of Tpx variants by inhibitors 1-5 and crosslinking agent $BM(PEG)_2$. Purified Tpx WT, Tpx C43S, Tpx C40S, Tpx W39A were incubated with a 3-fold excess of compounds 1-5 under reducing conditions and evaluated via intact protein-MS. Tpx WT was additionally crosslinked with $BM(PEG)_2$ and analyzed in the same way. The masses of the components, together with the calculated masses for the covalently modified Tpx constructs, and the detected masses via ESI-MS are shown in Dalton (Da). Values for unmodified proteins are shown in italics.

| Tpx (+ Compound) | $M_{components}$ / Da | $M_{calculated}$ / Da | $M_{detected}$ / Da |
|---|---|---|---|
| **Tpx $WT_{red}$** | **16076.2** | 16076.2 | *16076.0* |
| Tpx $WT_{red}$ **+ 1** | 16076.2 **+ 294.7** | 16334.4[a] | 16334.0 |
| Tpx $WT_{red}$ **+ 2** | 16076.2 **+ 294.7** | 16334.4[a] | 16333.0 |
| Tpx $WT_{red}$ **+ 3** | 16076.2 **+ 294.7** | 16334.4[a] | 16334.0 |
| Tpx $WT_{red}$ **+ 4** | 16076.2 **+ 344.7** | 16384.4[a] | 16384.0 |
| Tpx $WT_{red}$ **+ 5** | 16076.2 **+ 276.7** | 16316.4[a] | 16316.2 |
| Tpx $WT_{red}$ **+ $BM(PEG)_2$** | **2x** 16076.2 **+ 308.3** | 32460.7[b] | 32460.0 |
| **Tpx C43S** | **16060.1** | 16060.1 | *16059.7* |
| Tpx C43S **+ 1** | 16060.1 **+ 294.7** | 16318.3[a] | 16318.0 |
| Tpx C43S **+ 2** | 16060.1 **+ 294.7** | 16318.3[a] | 16318.0 |
| Tpx C43S **+ 3** | 16060.1 **+ 294.7** | 16318.3[a] | 16318.0 |
| Tpx C43S **+ 4** | 16060.1 **+ 344.7** | 16368.3[a] | 16368.0 |
| Tpx C43S **+ 5** | 16060.1 **+ 276.7** | 16300.3[a] | 16299.9 |
| **Tpx C40S** | **16060.1** | 16060.1 | *16059.7* |
| Tpx C40S **+ 1** | 16060.1 **+ 294.7** | 16318.3[a] | *16059.7* |
| Tpx C40S **+ 2** | 16060.1 **+ 294.7** | 16318.3[a] | *16059.8* |
| Tpx C40S **+ 3** | 16060.1 **+ 294.7** | 16318.3[a] | *16059.7* |
| Tpx C40S **+ 4** | 16060.1 **+ 344.7** | 16368.3[a] | *16059.7* |
| Tpx C40S **+ 5** | 16060.1 **+ 276.7** | 16300.3[a] | *16059.7* |
| **Tpx $W39A_{red}$** | **15961.1** | 15961.1 | *15961*.0 |
| Tpx $W39A_{red}$ **+ 1** | 15961.1 **+ 294.7** | 16219.3[a] | 16219.4 |
| Tpx $W39A_{red}$ **+ 2** | 15961.1 **+ 294.7** | 16219.3[a] | 16219.2 |
| Tpx $W39A_{red}$ **+ 3** | 15961.1 **+ 294.7** | 16219.3[a] | 16219.3 |
| Tpx $W39A_{red}$ **+ 4** | 15961.1 **+ 344.7** | 16269.3[a] | 16269.3 |
| Tpx $W39A_{red}$ **+ 5** | 15961.1 **+ 276.0** | 16200.6[a] | 16201.1 |

[a] Covalent **Tpx** complex with compounds **1-5** after HCl elimination (-36.5 Da)
[b] Covalent adduct of two **Tpx** molecules, crosslinked via $BMPEG_2$

**Table S2**. Cytotoxicity of compounds 1-5 against parasitic and human cells. $EC_{50}$ values of compounds 1-5 for parasitic *T. brucei brucei* 449 cells (Lister 427 strain, bloodstream form) were determined with the ATP-lite assay[5] after 24 and 48 h incubation (two biological replicates of four technical replicates, errors arise from standard deviation). Toxicity against human embryonic kidney (HEK293) cells was determined via resazurin assay[6] after 24 h incubation time (four technical replicates, errors arise from standard deviation and fitting). The selectivity index (SI) was calculated based on the values obtained from *in vivo* cell-based toxicity assays (24 h) by dividing the value obtained for human cells by that for *T. brucei* (error calculated following gaussian law of error propagation).

| Compound | Cells | $EC_{50}$ (24 h) / µM | $EC_{50}$ (48 h) / µM | $SI_{in\ vivo}$ (24 h) |
|---|---|---|---|---|
| *para*-CFT (**1**) | *T. brucei brucei* 449 | 1.3 ± 0.3 | 1.5 ± 0.3 | 3.0 ± 0.8 |
| | HEK293 | 3.9 ± 0.4 | / | |
| *meta*-CFT (**2**) | *T. brucei brucei* 449 | 1.3 ± 0.4 | 1.1 ± 0.5 | 5.0 ± 1.6 |
| | HEK293 | 6.5 ± 0.5 | / | |
| *ortho*-CFT (**3**) | *T. brucei brucei* 449 | 1.0 ± 0.3 | 0.8 ± 0.3 | 5.0 ± 1.6 |
| | HEK293 | 5.0 ± 0.6 | / | |
| CtFT (**4**) | *T. brucei brucei* 449 | 2.2 ± 0.6 | 2.5 ± 0.6 | 1.8 ± 0.5 |
| | HEK293 | 3.9 ± 0.3 | / | |
| CPT (**5**) | *T. brucei brucei* 449 | 0.6 ± 0.3 | 1.4 ± 0.4 | 8.2 ± 4.2 |
| | HEK293 | 4.9 ± 0.6 | / | |

**Table S3.** Crystal data and structure refinement for compounds 2-5. The crystal structure of isolated *para*-CFT (1) has been published in Wagner et al.[4]

| **Crystal data and structure refinement for *meta*-CFT (2)** | |
|---|---|
| Empirical formula | $C_{13}H_8ClFN_2OS$ |
| Formula weight | 294.7 |
| Temperature/ K | 120 |
| Crystal system | monoclinic |
| Space group | P $2_1$/n |
| a/ Å | 9.7655(7) |
| b/ Å | 9.6269(4) |
| c/ Å | 13.3940(9) |
| α/ ° | 90.0 |
| ß/ ° | 105.817(5) |
| γ/ ° | 90.0 |
| Volume/ $Å^3$ | 1211.51(13) |
| Z | 4 |
| $\rho_{calc}$/ g $cm^{-3}$ | 1.616 |
| μ/ $mm^{-1}$ | 0.491 |
| F(000) | 600 |
| Crystal size/ $mm^3$ | 0.11 x 0.22 x 0.30 |
| Radiation | Mo-Kα |
| 2θ range for data collection/ ° | 4 to 56 |
| Index ranges | -12 ≤ h ≤ 12 -12 ≤ k ≤ 12 -16 ≤ l ≤ 17 |
| Reflections collected | 8043 |
| Independent reflections | 2904 ($R_{int}$ = 0.0129) |
| Data/restraints/parameters | 2904/0/208 |
| Goodness-of-fit on $F^2$ | 1.068 |
| Final R indexes [I>2σ(I)] | R1= 0.0292, wR2= 0.0753 |
| Final R indexes [all data] | R1= 0.0329, wR2= 0.0782 |
| Largest diff. peak/hole / e$Å^{-3}$ | 0.44, -0.15 |
| **Crystal data and structure refinement for *ortho*-CFT (3)** | |
| Empirical formula | $C_{13}H_8ClFN_2OS$ |
| Formula weight | 294.7 |
| Temperature/ K | 120 |
| Crystal system | monoclinic |
| Space group | P $2_1$/n |
| a/ Å | 9.6949(5) |
| b/ Å | 9.5481(5) |
| c/ Å | 13.7042(7) |
| α/ ° | 90.0 |
| ß/ ° | 108.088(4) |
| γ/ ° | 90.0 |
| Volume/ $Å^3$ | 1205.87(11) |
| Z | 4 |
| $\rho_{calc}$/ g $cm^{-3}$ | 1.623 |
| μ/ $mm^{-1}$ | 0.493 |
| F(000) | 600 |
| Crystal size /$mm^3$ | 0.04 x 0.18 x 0.41 |
| Radiation Mo-Kα | Mo-Kα |
| 2θ range for data collection/ ° | 4 to 56 |
| Index ranges | -12 ≤ h ≤ 11 -12 ≤ k ≤ 12 -17 ≤ l ≤ 18 |
| Reflections collected | 6502 |
| Independent reflections | 2874 ($R_{int}$ = 0.0248) |
| Data/restraints/parameters | 2874/1/203 |
| Goodness-of-fit on $F^2$ | 1.023 |
| Final R indexes [I>2σ(I)] | R1= 0.0371, wR2= 0.0844 |
| Final R indexes [all data] | R1= 0.0559, wR2= 0.0923 |
| Largest diff. peak/hole / e$Å^{-3}$ | 0.37, -0.31 |

**Table S3, continued**.

| Crystal data and structure refinement for CtFT (4) | |
|---|---|
| Empirical formula | $C_{14}H_8ClF_3N_2OS$ |
| Formula weight | 344.7 |
| Temperature/ K | 120 |
| Crystal system | monoclinic |
| Space group | P $2_1$ |
| a/ Å | 10.7196(7) |
| b/ Å | 7.9153(3) |
| c/ Å | 16.2165(11) |
| α/ ° | 90.0 |
| ß/ ° | 92.519(5) |
| γ/ ° | 90.0 |
| Volume/ $Å^3$ | 1374.63(14) |
| Z | 4 |
| $\rho_{calc}$/ g $cm^{-3}$ | 1.666 |
| μ/ $mm^{-1}$ | 0.466 |
| F(000) | 696 |
| Crystal size/ $mm^3$ | 0.1 x 0.14 x 1.3 |
| Radiation | Mo-Kα |
| 2θ range for data collection/ ° | 4 to 56 |
| Index ranges | -14 ≤ h ≤ 14 -10 ≤ k ≤ 9 -21 ≤ l ≤ 21 |
| Reflections collected | 15442 |
| Independent reflections | 6426 ($R_{int}$ = 0.0343) |
| Data/restraints/parameters | 6426/1/459 |
| Goodness-of-fit on $F^2$ | 1.086 |
| Final R indexes [I>2σ(I)] | R1= 0.0465, wR2= 0.1137 |
| Final R indexes [all data] | R1= 0.0612, wR2= 0.1201 |
| Largest diff. peak/hole/ e$Å^{-3}$ | 0.42, -0.38 |
| **Crystal data and structure refinement for CPT (5)** | |
| Empirical formula | $C_{13}H_9ClN_2OS$ |
| Formula weight | 276.7 |
| Temperature/ K | 120 |
| Crystal system | monoclinic |
| Space group | C 2/c |
| a/ Å | 21.2954(15) |
| b/ Å | 13.2035(13) |
| c/ Å | 8.6076(6) |
| α/ ° | 90.0 |
| ß/ ° | 97.275(6) |
| γ/ ° | 90.0 |
| Volume/ $Å^3$ | 2400.7(3) |
| Z | 8 |
| ρcalc/g$cm^{-3}$ | 1.531 |
| μ/ $mm^{-1}$ | 0.479 |
| F(000) | 1136 |
| Crystal size /$mm^3$ | 0.11 x 0.48 x 0.70 |
| Radiation | Mo-Kα |
| 2θ range for data collection /° | 4 to 56 |
| Index ranges | -27 ≤ h ≤ 27 -16 ≤ k ≤ 17 -8 ≤ l ≤ 11 |
| Reflections collected | 5925 |
| Independent reflections | 2856 ($R_{int}$= 0.0166) |
| Data/restraints/parameters | 2856/0/198 |
| Goodness-of-fit on $F^2$ | 1.093 |
| Final R indexes [I>2σ(I)] | R1= 0.0329, wR2= 0.0787 |
| Final R indexes [all data] | R1= 0.040, wR2= 0.0841 |
| Largest diff. peak/hole / e$Å^{-3}$ | 0.43, -0.18 |

**Table S4.** $^{19}F$-NMR signal chemical shifts and linewidths of free and Tpx-bound inhibitors 1-4. Chemical shifts were referenced to TFA. For Tpx-bound molecules, samples were pre-incubated and purified via SEC as described in the material and methods section. To obtain spectra for free compounds 1-4 in Tpx buffer, 8 or 13 vol% DMSO were added to dissolve the molecules, requiring the introduction of a correction summand for the resulting $^{19}F$-chemical shifts (corrections are shown in the bottom right and are already included in the chemical shifts shown in this table). 1D $^{19}F$ NMR spectra are shown in Fig. S19.

| Tpx/inhibitor complexes | conc. / µM | Tpx WT + inhibitor chemical shift / ppm | Tpx WT + inhibitor linewidth / Hz | Tpx W39A + inhibitor chemical shift / ppm | Tpx W39A + inhibitor linewidth / Hz |
|---|---|---|---|---|---|
| *para*-CFT (**1**) | 750 | -115.58 | 118 | -115.07 | 84 |
| | 500 | -115.58 | 111 | -115.02 | 80 |
| | 250 | -115.58 | 118 | -114.95 | 70 |
| | 100 | -115.52 | 144[a] | -114.88 | 54 |
| | 50 | -115.54 | /[a] | -114.85 | 41 |
| *meta*-CFT (**2**) | 750 | -114.43 | 102 | -114.73 | 56 |
| | 500 | -114.43 | 100 | -114.74 | 54 |
| | 250 | -114.43 | 103 | -114.75 | 50 |
| | 100 | -114.44 | 96 | -114.77 | 46 |
| | 50 | -114.42 | 93 | -114.78 | 45 |
| *ortho*-CFT (**3**) | 750 | -114.41 | 307 | -115.28 | 63 |
| | 500 | -114.41 | 320 | -115.31 | 53 |
| | 250 | -114.32 | 324 | -115.35 | 56 |
| | 100 | -114.41 | /[a] | -115.38 | 52 |
| | 50 | /[a] | /[a] | -115.39 | 51 |
| CtFT (**4**) | 750 | -62.80 | 96 | -62.96 | 33 |
| | 500 | -62.82 | 98 | -62.97 | 32 |
| | 250 | -62.85 | 80 | -62.98 | 32 |
| | 100 | -62.89 | 74 | -62.98 | 31 |
| | 50 | -62.92 | 58 | -62.98 | 30 |

| Free inhibitors | conc. / µM | chemical shift[b] / ppm | linewidth / Hz | DMSO / vol.% | correction / ppm |
|---|---|---|---|---|---|
| *para*-CFT (**1**) | 100 | -115.98 | 31 | 8 | -0.15 |
| *meta*-CFT (**2**) | 100 | -115.50 | 31 | 8 | -0.15 |
| *ortho*-CFT (**3**) | 100 | -116.47 | 31 | 8 | -0.15 |
| CtFT (**4**) | 100 | -63.11 | 26 | 13 | -0.26 |

[a]poor signal to noise ratio

[b]chemical shifts for free inhibitors already include DMSO correction term

## E. Supporting Information Figures

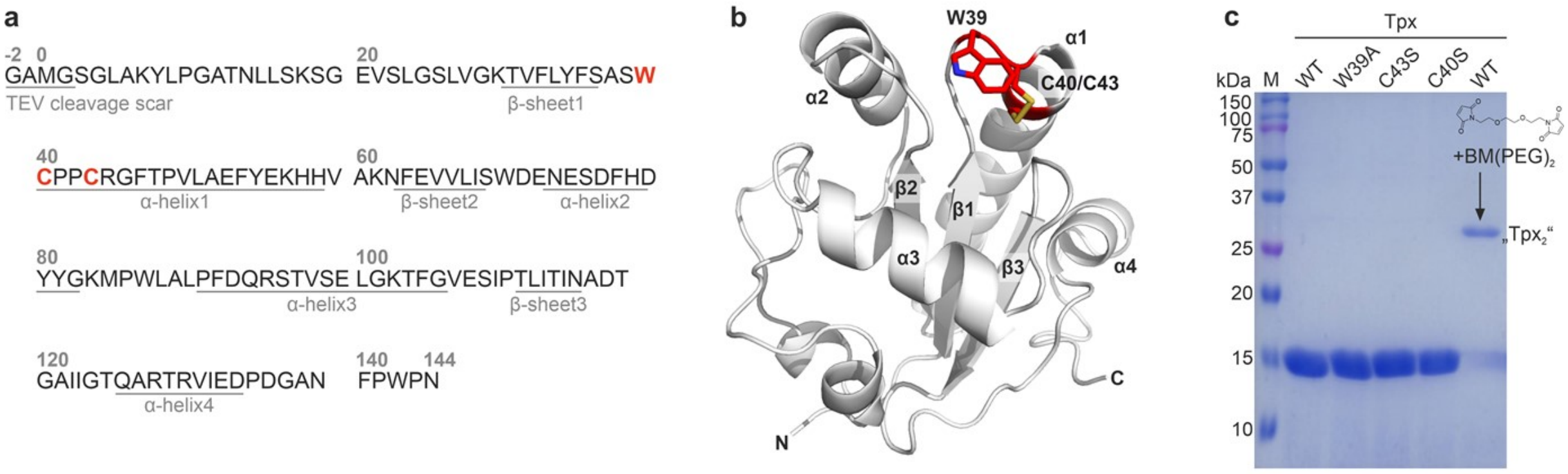


**Fig. S1.** Tpx constructs used in this study. a, Sequence of the *T. brucei* Tpx wildtype (WT) protein (Uniprot accession: O77404). Tpx was purified with an N-terminal TEV protease recognition site, resulting in replacement of methionine residue 1 with a "GAMG" motif as a scar from TEV-cleavage in the final construct. Major secondary structure motifs (consisting of at least five amino acids) are underlined and numbered. Residues mutated in this study (W39, C40, C43) are highlighted in red. b, Tertiary structure of *T. brucei* Tpx WT. For clarity, we show the AlphaFold prediction (AF-O77404-F1-model_v4[31,32]) where the W39 indole conformation agrees with other published Tpx structures (e.g. *Crithidia fasciculata* Tpx1, PDB: 1EWX[34], and *Leishmania major* Tpx1, PDB: 3S9F[33]) instead of the crystal structure of *T. brucei* Tpx (PDB:1O73)[35], where this residue adopts a different conformation (see also Fig. S13). The RMSD between the *T. brucei* Tpx WT crystal structure (PDB: 1O73[35]) and the AlphaFold prediction (AF-O77404-F1-model_v4[31,32]) is 0.55 Å. c, Purified Tpx WT, Tpx W39A, Tpx C43S, Tpx C40S (all ~16 kDa), and $BM(PEG)_2$-crosslinked Tpx WT (~32 kDa, "$Tpx_2$") on a 15% SDS-PAGE gel (Coomassie-stained, marker: Biorad Precision Plus Protein Dual Color Standard). The structural formula of the Tpx crosslinker 1,8-bismaleimido-diethyleneglycol ($BM(PEG)_2$) is shown in the last lane, above the protein band of crosslinked Tpx ("$Tpx_2$").

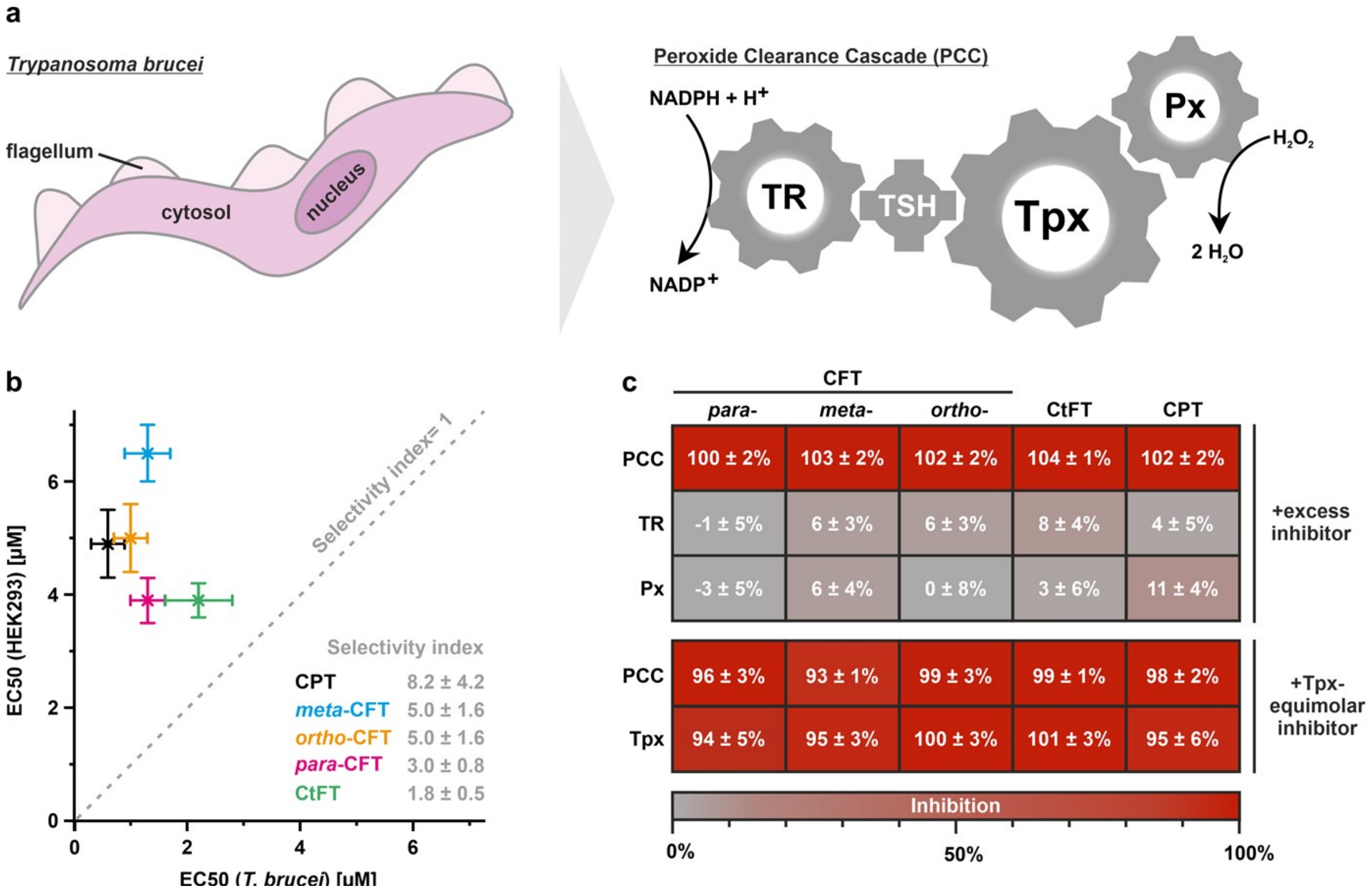


**Fig. S2**. Compounds 1-5 are highly cytotoxic, but selective Tpx inhibitors *in vitro*. a, Schematic representation of a *Trypanosoma brucei* (*T. brucei*) parasite (left) and the cytosolic peroxide clearance cascade (PCC) consisting of trypanothione reductase (TR), trypanothione (TSH), tryparedoxin (Tpx), and a peroxidase (Px). In each enzymatic step, the transfer of reducing equivalents is accomplished through a thiol exchange reaction, which highlights the large number of reactive cysteine species as potential inhibitor off-targets in the parasitic redox cascade. b, Half-maximal effective concentration (EC50) values of 1-5 in cell viability assays with *T. brucei brucei* 449 (strain: Lister 427, bloodstream form) and HEK293 cells (n= 8 and n=4, respectively, see also Table S1). Data points represent the mean value ± standard deviation. The inset table shows selectivity indices, calculated as the fraction of the corresponding EC50 values for Trypanosoma and HEK293 cells (Table S1). c, Top rows: Relative enzyme inhibition after treatment of the entire PCC, or TR and Px separately, with excess amounts of inhibitors 1-5 (for details, see material and methods section). Bottom rows: Treatment of the entire PCC or Tpx alone with Tpx-equimolar amounts of compounds 1-5.

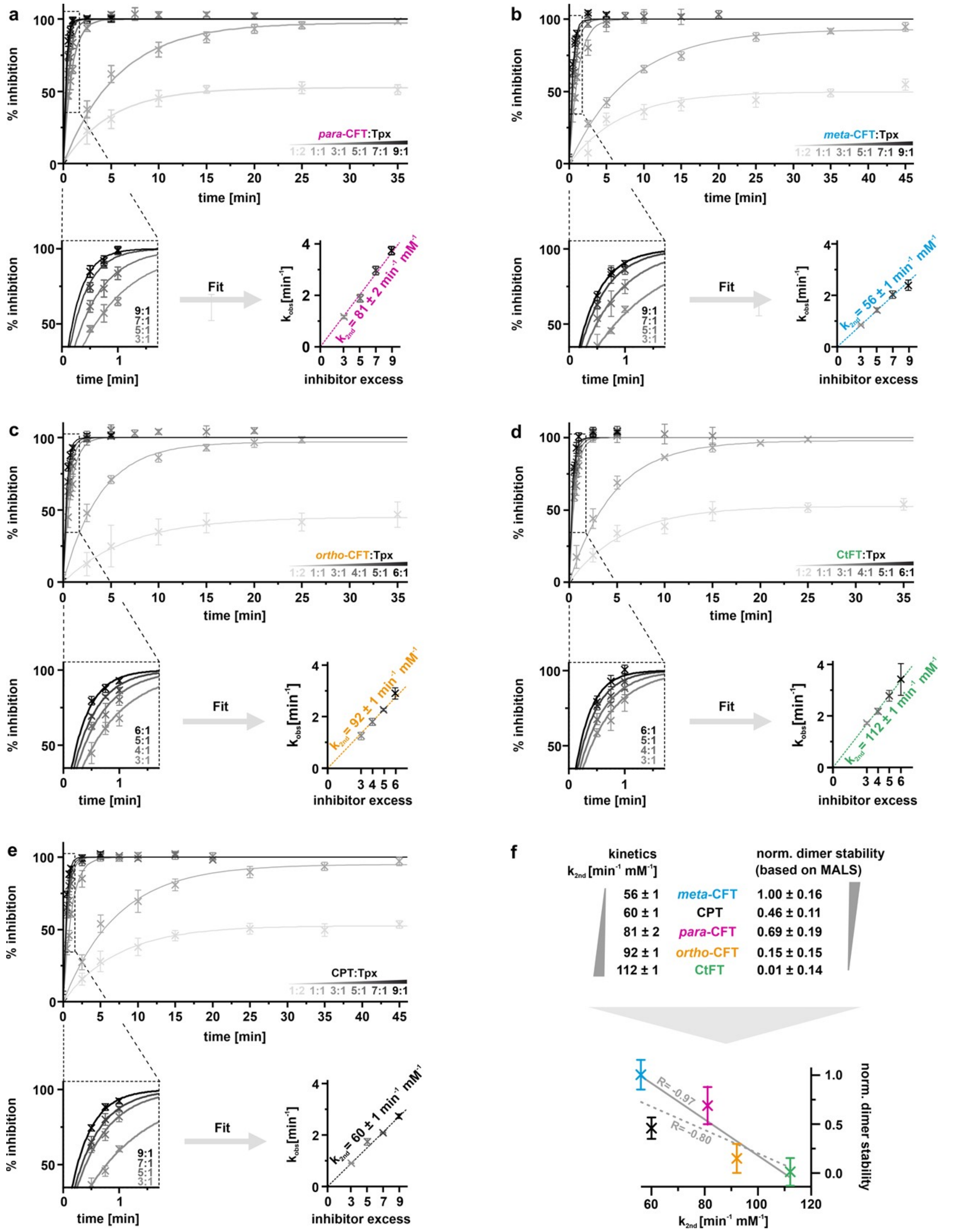


**Fig. S3**. Kinetics of covalent Tpx inhibition are fluorination dependent and inversely correlated with induced dimer affinity. a-e, Using the reconstituted peroxide clearance cascade, time-resolved inhibition curves of 5 µM Tpx with substoichiometric, equimolar and excess amounts of inhibitors 1-5 (3-, 5-, 7-, and 9-fold excess for slow inactivators (1-2, 5); 3-, 4-, 5-, and 6-fold excess for fast inactivators (3-4)) were recorded at 25 °C. Curves were fitted as a one phase exponential association with $f(x)= I_{max}*(1-exp(-k_{obs}*x))$, with $I_{max}$ as the inhibition limit and $k_{obs}$ as the observed experimental inhibition constant (a-e top). A close-up (bottom left in each panel) shows inhibition shortly after addition of excess amount of inhibitor. Observed reaction rate constants ($k_{obs}$) were plotted against inhibitor concentration, shown here as x-fold inhibitor excess. The slope of a linear regression fit (dashed line) yielded second order rate constants $k_{2nd}$ (bottom right in each panel). Data are shown as mean value ± standard deviation of three technical triplicates. f: Correlation of inhibition kinetics ($k_{2nd}$) with dimer affinities (based on SEC-MALS with 100 µM Tpx in complex with inhibitor). Data were normalized to the most stable complex (Tpx/*meta*-CFT) as assessed by ITC, SEC and SEC-MALS (see Fig. 1 and main text). Correlation yielded negative Pearson R values (fluorinated inhibitors (1-4): solid line; all inhibitors (1-5): dashed line), showing that inhibition kinetics are slow for potent Tpx dimerizers and vice versa.

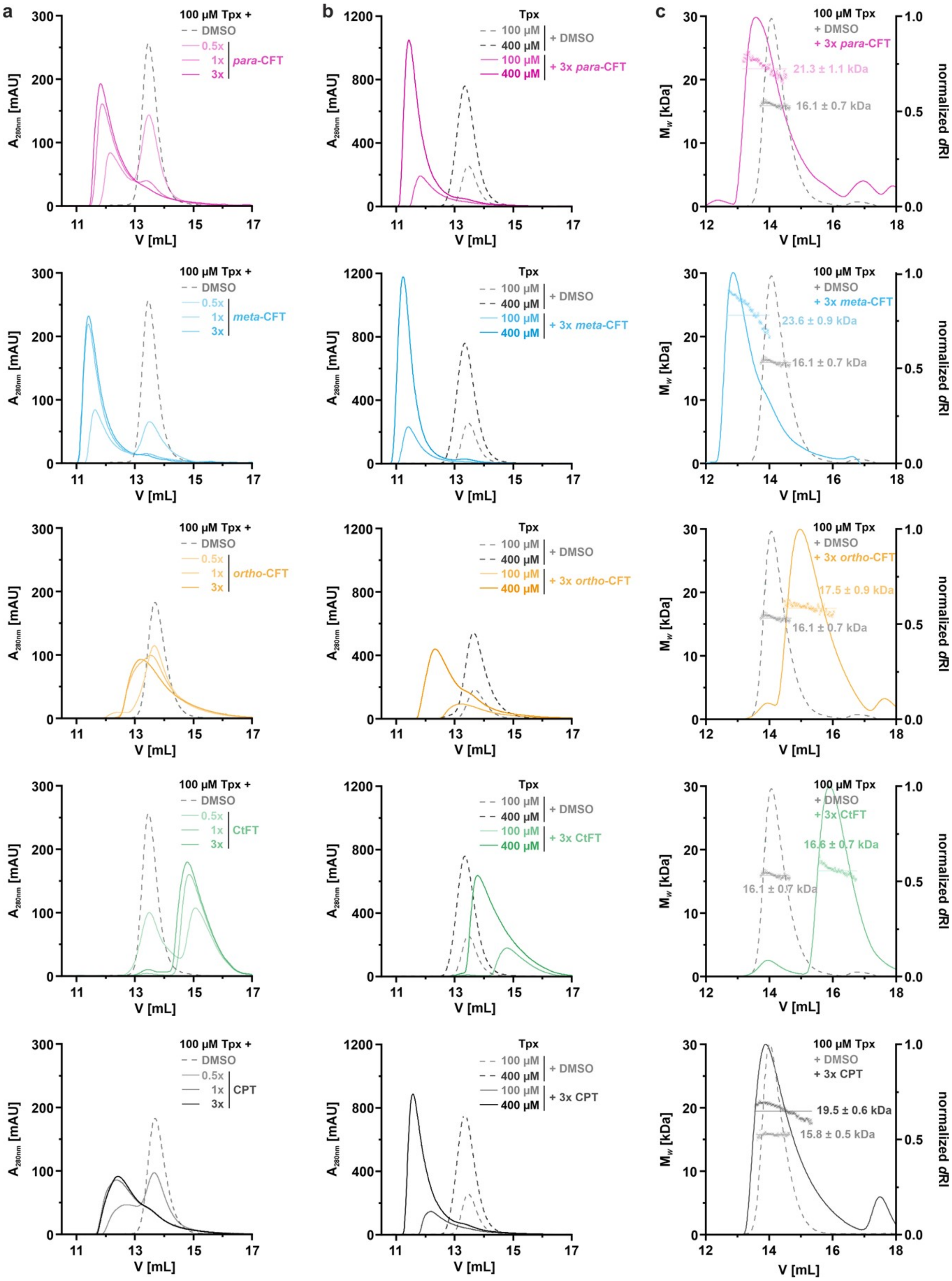

**Fig. S4.** Analytical SEC and SEC-MALS of Tpx and Tpx/inhibitor complexes. a, Elution profiles of reduced Tpx WT (100µM) with 7.5% DMSO (grey dashed line), and with inhibitors 1-5 at substoichiometric (1:0.5), equimolar (1:1) and excess (1:3) molar amounts (solid lines). Inhibitor binding caused smaller Tpx elution volumes except for CtFT (4), which does not lead to notable dimerization at 100 µM protein concentration and enhances column interactions (see Fig. S5). b, Concentration dependent elution profiles of 100 µM or 400 µM unmodified Tpx in the reduced state (with 7.5% and 30% DMSO, respectively) compared to Tpx/inhibitor complexes generated by threefold molar excess of inhibitor. In all cases, a higher protein concentration shifted the Tpx elution volume towards the permanent, BM(PEG)$_2$-linked dimer (V= 11.3 mL). c, SEC-MALS of Tpx and Tpx/inhibitor complexes (100 µM each) with changes in refractive index (*d*RI, right y-axis) and average molar mass ($M_w$, shown as crosses (left y-axis)). Except for the CtFT complex, higher $M_w$ were observed for modified than unmodified Tpx.

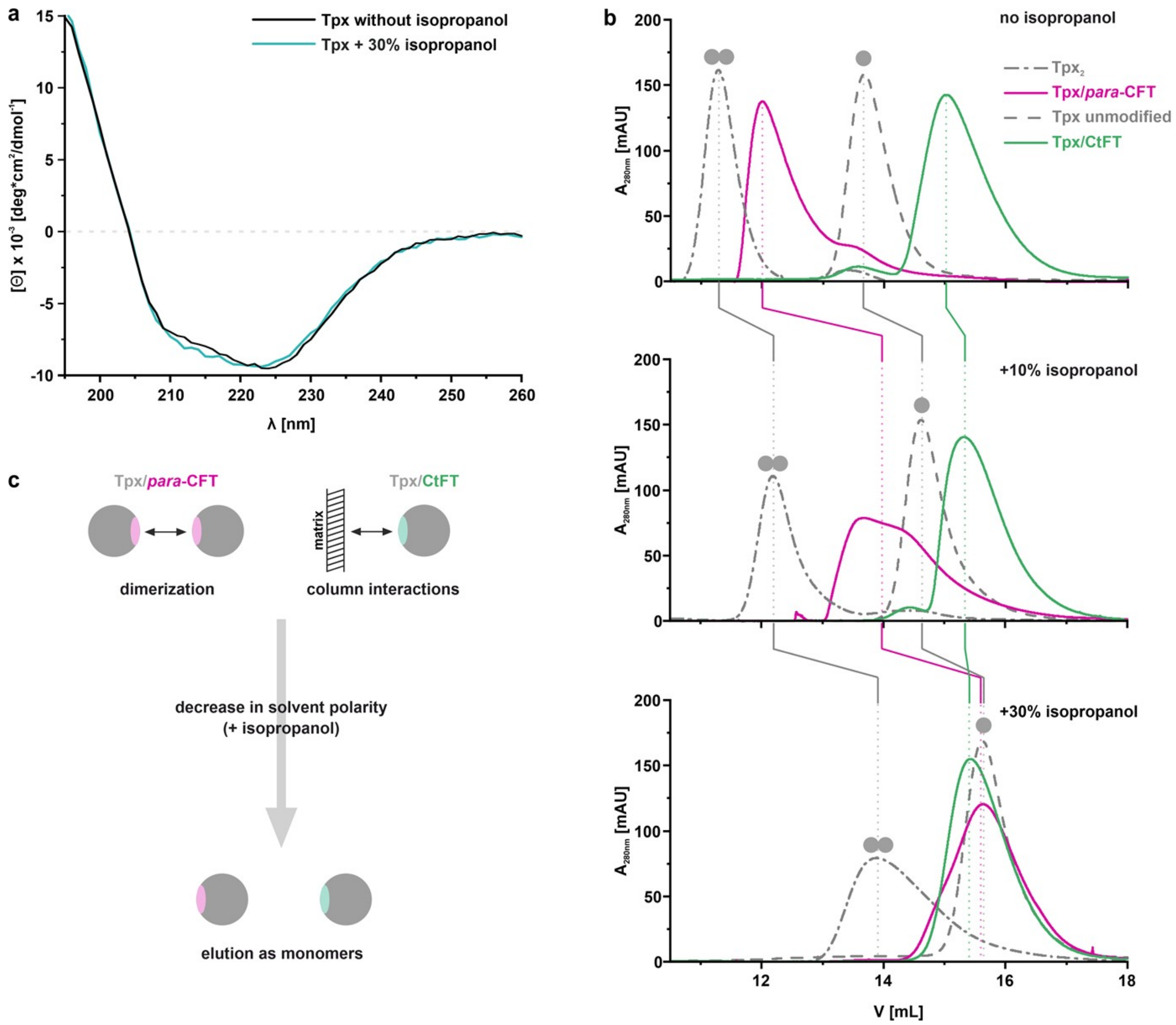


**Fig. S5.** Delayed SEC elution of the Tpx WT/CtFT complex is due to secondary column interactions. a, To mimic the length of a SEC run, Tpx was incubated for 30 min at room temperature in SEC buffer without (black) or complemented with 30% v/v isopropanol (petrol). Circular dichroism (CD) spectra show that the structural integrity of Tpx was not affected by isopropanol. b, SEC elution profiles of Tpx monomers (Tpx, dashed grey line), BM(PEG)$_2$-crosslinked Tpx dimers (Tpx$_2$, dot-dashed grey line), Tpx/*para*-CFT (pink line) and Tpx/CtFT complexes (green line) in SEC buffer without isopropanol (top), with 10% v/v isopropanol (middle), and with 30% v/v isopropanol (bottom). Isopropanol increases the hydrophobicity of the mobile phase and thus weakens hydrophobic interactions of analytes with themselves (e.g. dimerization) as well as with the column matrix. For the monomeric and dimeric Tpx standard, the oligomeric state is indicated by grey spheres above the respective elution peaks. In buffer without isopropanol, Tpx/p*ara*-CFT dimers eluted earlier and Tpx/CtFT later than unmodified Tpx. In buffer with 30% v/v isopropanol, unmodified and inhibitor-bound Tpx eluted similarly, suggesting that all proteins are monomeric under these conditions. In contrast, the permanent, crosslinked Tpx dimer, albeit also affected by the solvent polarity, still eluted at smaller elution volumes showing that the column still separated by particle size under these conditions. c, Model for secondary analyte interactions that affect the SEC elution volume. At high solvent polarity (no isopropanol), dimer interactions reduce the Tpx/*para*-CFT elution volume, while for Tpx/CtFT, column interactions lead to the observed larger elution volume. With 30% v/v isopropanol, both protein/inhibitor complexes elute as monomers because attractive dimer and column interactions are weakened. Increased SEC-elution volumes due to hydrophobic interactions with the column have been reported previously.[8,36]

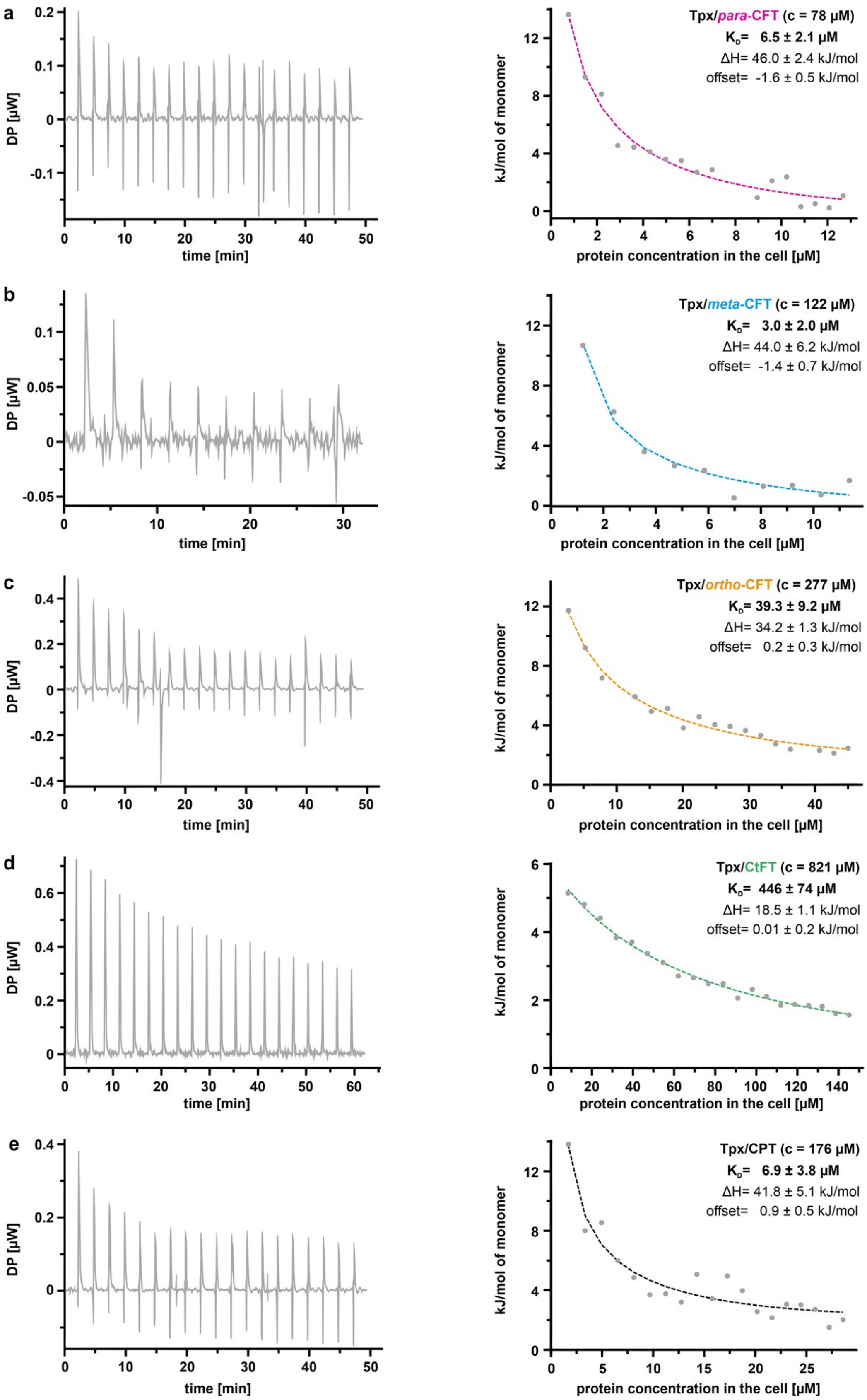


**Fig. S6.** Dissociation constants ($K_D$) for $[Tpx/inhibitor]_2$ dimers. a-e, Dilution isothermal titration calorimetry (ITC) of covalent Tpx/inhibitor complexes. Thermograms after serial injections of the analyte into Tpx buffer are shown in the left panels. Integrals of thermal signals were fitted to monomer equivalents in the cell (right panels) to yield dissociation constants $K_D$, dissociation enthalpies $\Delta$H, and offsets as fitting parameters. Protein concentration in the syringe is given in brackets. Each measurement was performed in a technical triplicate (for simplicity, only one replicate is shown here for each analyte). Dimer dissociation was endothermic, and integrals decreased with increasing analyte concentration in the measurement cell.

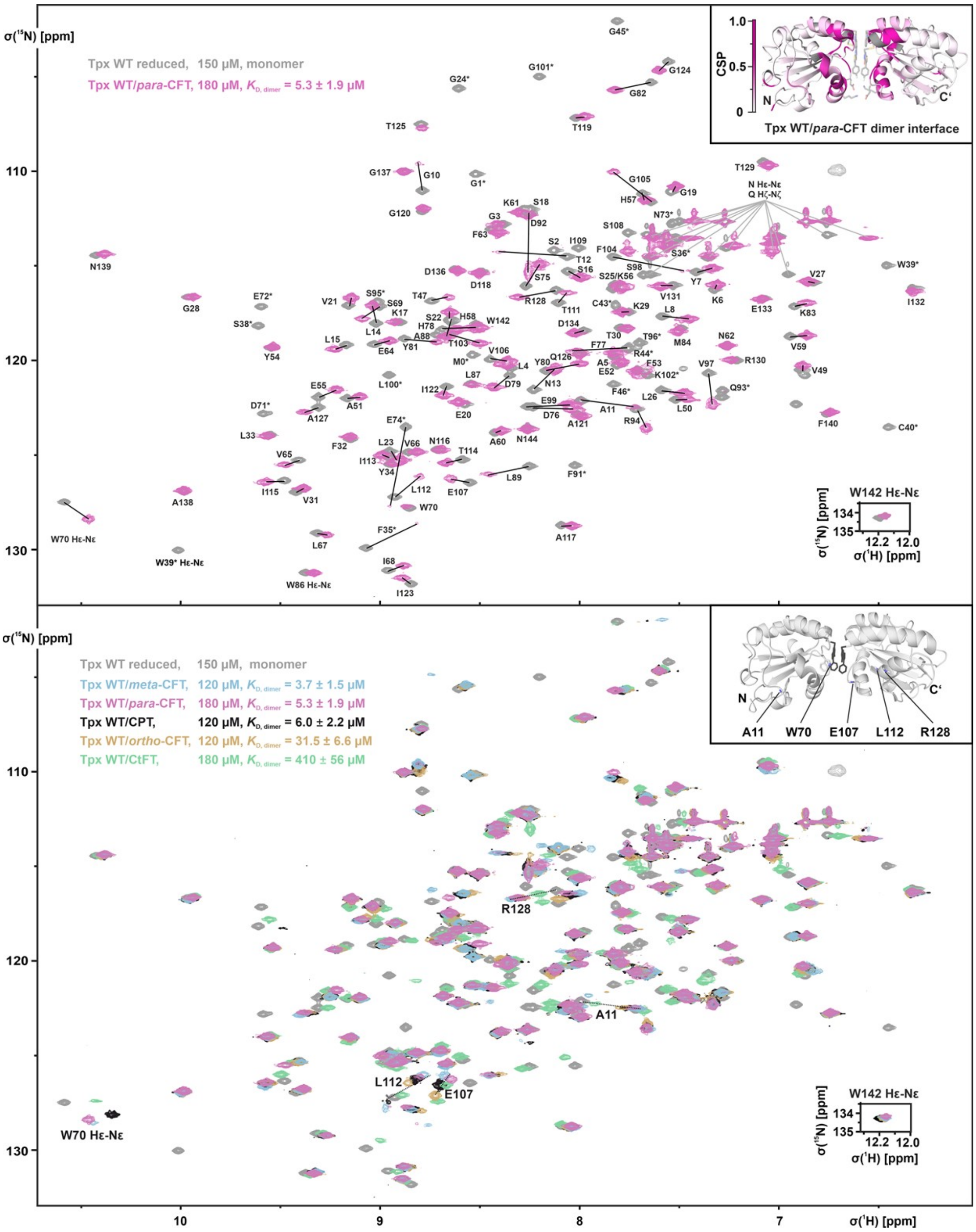


**Fig. S7.** Tpx WT interactions with inhibitors 1-5 monitored by solution NMR spectroscopy. Top: 2D $^{1}H$, $^{15}N$-HSQC spectra of unmodified, monomeric $^{15}N$-labeled Tpx WT in the reduced state (150 µM, black) and the dimeric Tpx WT/*para*-CFT complex (180 µM, pink). The backbone assignments for the *para*-CFT bound state were transferred from our previous assignment for the reduced Tpx WT[2], and verified through additional 3D experiments. Signals displaying line broadening upon inhibitor binding (not visible in the spectrum) are marked with an asterisk. The dissociation constant ($K_D$) of *para*-CFT (1)-induced Tpx dimerization (top left) was obtained from dilution ITC. Bottom: $^{1}H$, $^{15}N$-HSQC spectra of free and inhibitor bound Tpx WT (concentrations and dimer $K_D$s shown in the top left). Backbone assignments were transferred from our previous assignment of reduced Tpx WT[2] and, where necessary, verified through additional 3D experiments for the inhibitor-bound states. Inhibitor binding led to signal broadening of residues constituting the dimer interface, and additional chemical shift perturbations of backbone and side chain resonances of residues throughout the protein. Inhibitor fluorination pattern-dependent chemical shift trajectories for selected amide resonances (A11, W70 side chain, E107, L112 and R128) are indicated by dotted lines. In most cases, the extent of the chemical shift followed the dissociation constants $K_D$ of the inhibitor-induced dimers, suggesting that Tpx dimerization is the primary cause. Trajectories not following this trend included residues in direct contact with inhibitors 1-5 (e.g. E107 amide backbone and W70 side chain, see Fig. 2b as well as main text for details). The dissociation constants ($K_D$) for inhibitor-induced Tpx dimerization were obtained from dilution ITC.

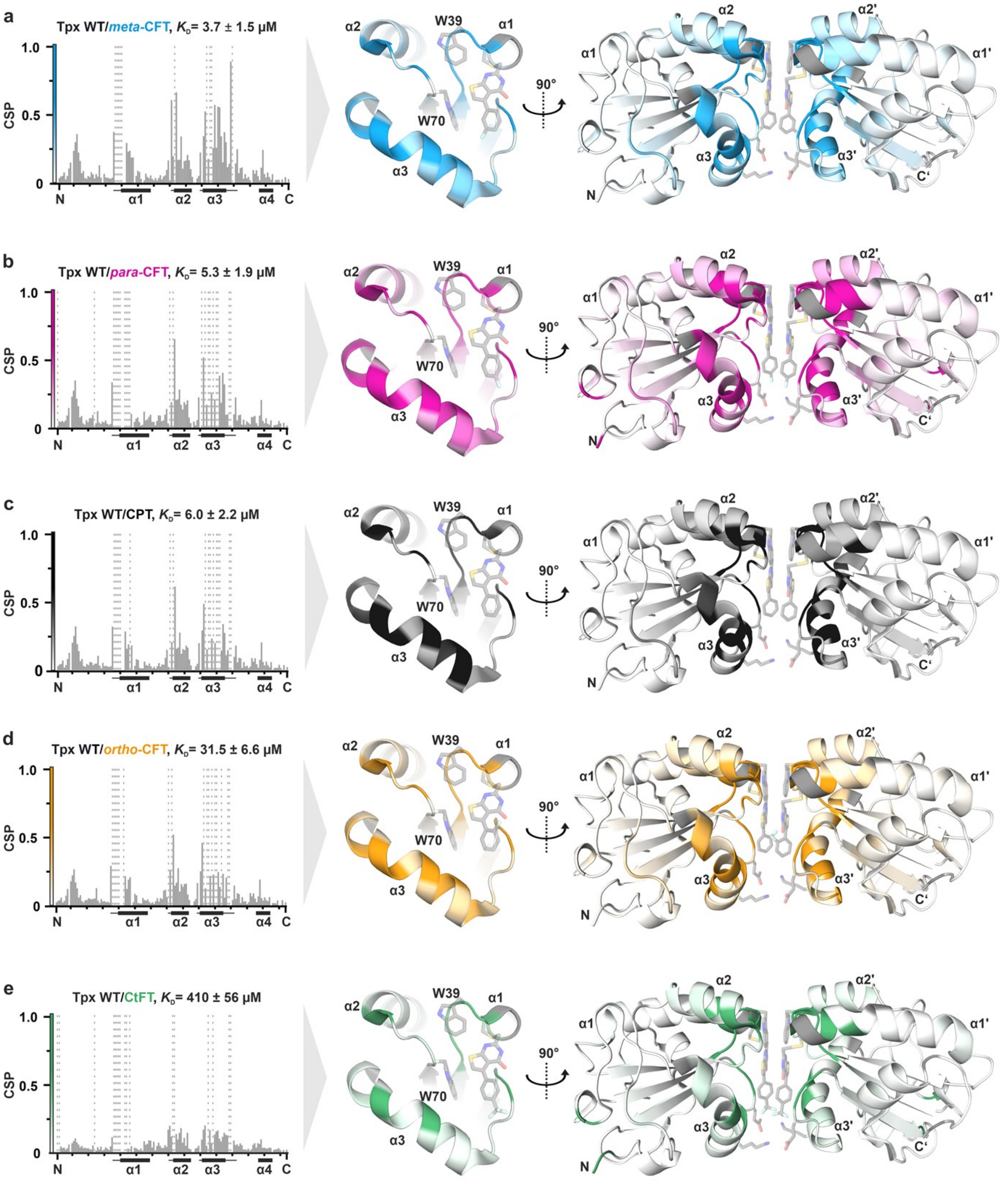


**Fig. S8.** Inhibitor interactions and chemically induced dimerization of Tpx WT monitored by $^{1}$H, $^{15}$N-NMR spectroscopy. a-e, Inhibitor induced chemical shift perturbations (CSP) in $^{1}$H, $^{15}$N-HSQC spectra of Tpx WT (see Fig. S7). The CSP plots (left) start with residue G1 and end with N144 (indicated with "N" and "C", respectively), ticks indicate increments of ten, starting with G10. The Tpx α-helices are indicated by black cylinders on the x-axis and adjacent loops with high CSP are indicated by lines. Signals displaying line broadening are shown as dashed bars. For each inhibitor, CSP upon inhibitor binding were also mapped onto the structure of Tpx/*para*-CFT dimers (PDB: 6GXG[4], Chains A and B), and are shown in color on an open book representation of the Tpx dimer interface (center), and in the context of the induced Tpx dimer (right, copied from Fig. 2a for clarity). All Tpx dimer interfaces consist of the respective inhibitor, Tpx α-helices 1-3, and loops containing W39 and W70 (for simplicity, W70 is not shown in the Tpx dimer).

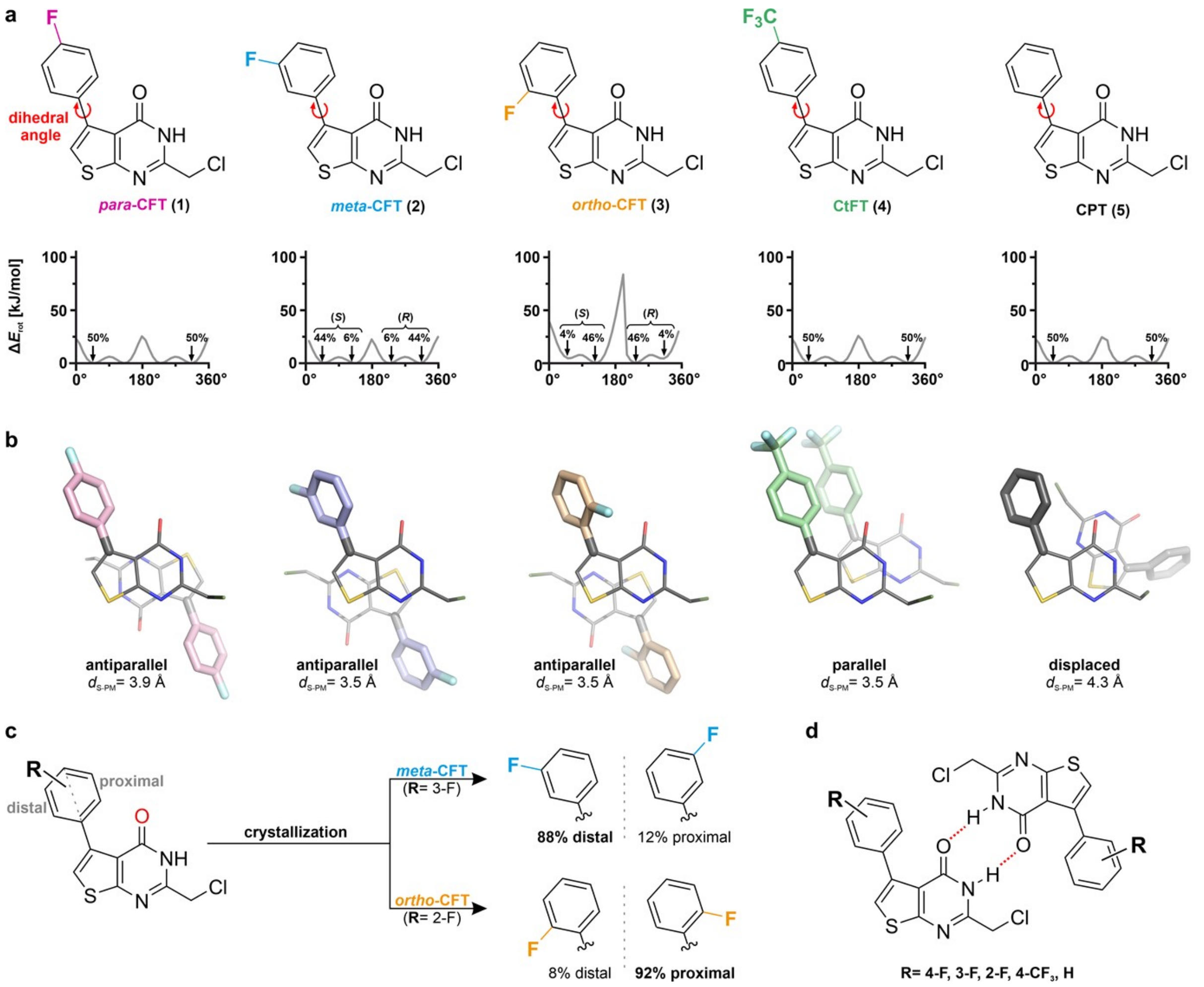


**Fig. S9.** Density functional theory (DFT) calculations and crystal structures of isolated compounds 1-5. a, Structural formulas and energetic profiles of rotamers of compounds 1-5. Beginning with the rotamer depicted (= 0°), the dihedral angle between phenyl and thiophene was increased in steps of 10° and the respective rotamer's energy was calculated using DFT. The molecule *ortho*-CFT (3) shows a rotational barrier of ~80 kJ/mol at 180° due to repulsive forces between the molecule's fluorine and oxygen. Rotamers seen by X-ray crystallography for the isolated compounds (b, c) are indicated with arrows, the respective populations are noted in %. Axial pro-chirality is indicated above. The dihedral angle observed for isolated *para*-CFT (47°) agreed with the dihedral angles observed for Tpx-bound *para*-CFT (47° and 52° for chains A and B, respectively) and was therefore used to set up MD simulations of monomeric and dimeric Tpx/inhibitor complexes. b, X-ray crystal structures of isolated compounds 1-5. The thienopyrimidinone core is shown in grey, and phenyl moieties of *para*-CFT in pink, *meta*-CFT in cyan, *ortho*-CFT in tan, CtFT in green and CPT in grey. Sulfur is shown in yellow, and fluorine in light blue. In the crystal unit cell, inhibitors were found to stack as antiparallel (1-3), parallel (4) or "displaced" (5) dimers. In the antiparallel orientation, the thienopyrimidinone rings are turned by 180° respective to each other. This allows the thiophene rings to stack and, typical for a face-to-face π-stacking interaction[37–39], adopt a distance of 3.5 to 3.9 Å between the sulfur and the pyrimidinone ring centroid ($d_{S\text{-}PM}$). Similarly, in the Tpx/*para*-CFT dimer structure, the $d_{S\text{-}PM}$ between two parallelly oriented *para*-CFT molecules is 3.6 Å.[4] Interestingly, we observed a similar $d_{S\text{-}PM}$ for isolated CtFT (4), even though this compound crystallized in a parallel orientation. This suggests that π-stacking between thiophen-sulfur and pyrimidinone is a reccurring, energetically favored interaction of these thienopyrimidinone-scaffold molecules. CtFT (4) is the only compound with interacting phenyl moieties due to the parallel stacking mode in the crystal structure. The electron-deprived phenyl rings display an ~85° angle with a phenyl/phenyl centroid distance of 4.9 Å, suggestive of stabilization via T-shaped π-interactions.[37–39] CPT (5) molecules show the least aromatic overlap resulting in the "displaced" orientation. c, Population of the respective fluorophenyl ring rotamers in *meta*- and *ortho*-CFT crystal structures relative to the inhibitor's oxygen atom. The fluorine in *ortho*-CFT (3) is mainly oriented towards the inhibitor's carbonyl group (proximal, 92%), which could be facilitated by a fluorine lone pair interaction with the carbonyl carbon (nb/π*-interaction).[40] The opposite orientation (distal) is more populated in *meta*-CFT crystals (88%) which might result from repulsive interactions between the inhibitor's fluorine and oxygen atoms. d, Amide groups in all compounds interacted with a symmetry mate via hydrogen bonds (red dashed lines). The CCDC deposition numbers of the structures of isolated compounds 1-5 are 1862408, 2421180, 2421181, 2421182, and 2423222.

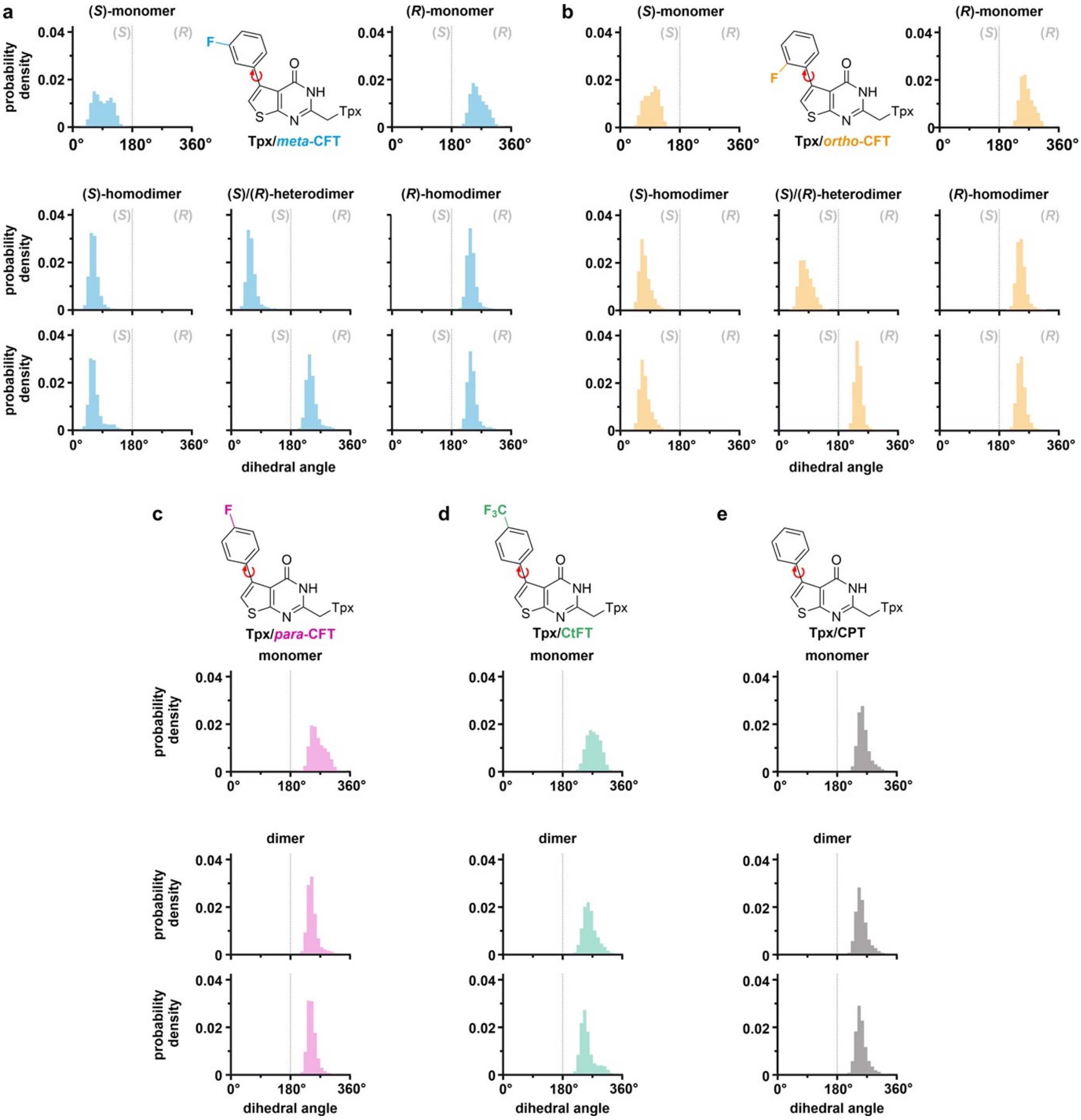


**Fig. S10.** Phenyl/thiophen dihedral angle distributions of monomeric and dimeric Tpx WT/inhibitor complexes *in silico*. Five independent 100 ns molecular dynamics simulations were carried out per atropisomer for a, Tpx/*meta*-CFT, b, Tpx/*ortho*-CFT, c, Tpx/*para*-CFT, d, Tpx/CtFT, e, Tpx/CPT. In all cases, the orientation of protein chain A (monomers) or chain A and chain B (dimers) and the inhibitor molecule of the crystal structure of the Tpx/*para*-CFT complex (PDB: 6GXG[4]) were used as starting models (see main text for details). The number of frames displaying a specific rotamer over the course of the simulations is shown for each inhibitor. For Tpx/inhibitor complexes with *meta*- and *ortho*-CFT (2, 3), the (*S*)- and (*R*)-atropisomers with fluorine pointing towards the binding pocket ("F-in") or towards the solvent ("F-out") were simulated. For Tpx dimers, this also included "homodimeric" complexes with both inhibitors in the same, and "heterodimeric" complexes with inhibitors in opposite directions. In all simulations, both in the monomer and dimer, rotation of the phenyl substituent was found to be restrained by the protein, and the accessible dihedral angles were limited to the respective ensemble between the rotational barriers at 0° (or 360°) and 180° (see also Fig. S9). This suggests that once Tpx/inhibitor complexes with *meta*- and *ortho*-CFT (**2**, **3**) are formed, they are chiral. However, the simulations do not allow us to gauge which atropisomer is energetically more stable and, even though we did not observe racemization throughout the 100 ns MD simulations, we cannot rule out that (*R*)- and (*S*)-atropisomers are interconverted over a longer time. Of note, most dihedral angle distributions are narrower in the dimer compared to the respective monomeric Tpx/inhibitor complex, indicating that dimerization further restrains rotation of the inhibitor's phenyl substituent.

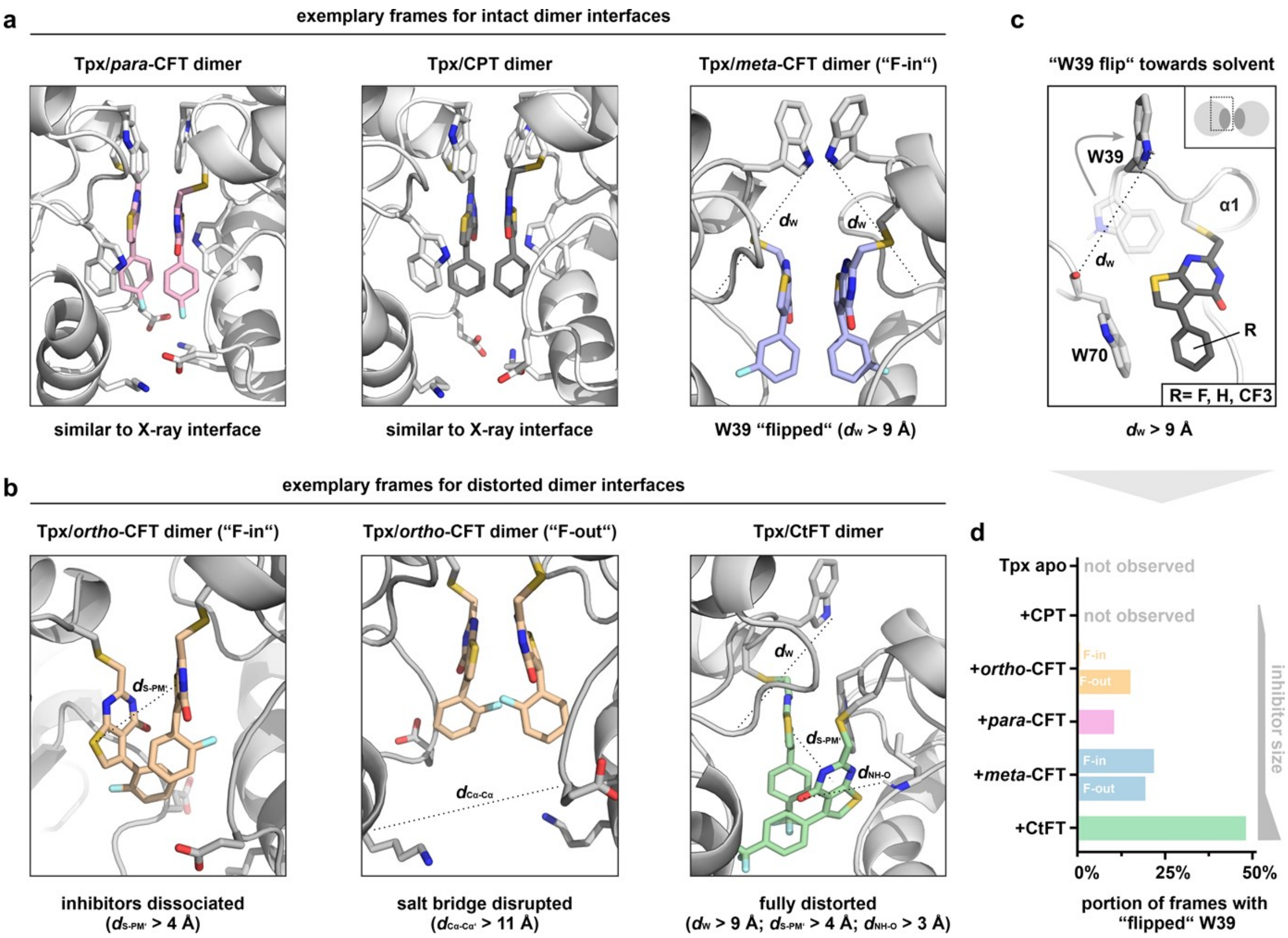


**Fig. S11.** Exemplary MD-frames of intact (a) and distorted (b) Tpx dimer interfaces with inhibitors 1-5. Exceeding critical distance thresholds (indicated below each panel) resulted in dimer interface distortion and partial complex dissociation. Interactions deemed relevant for stable dimer formation are highlighted by dashed lines and comprise inhibitor/inhibitor interactions ($d_{S\text{-}PM'}$ between thiophene sulfur and pyrimidinone centroid), salt bridges between charged residues ($d_{C\alpha\text{-}C\alpha'}$ between K102/E107') and inhibitor interactions with the protein binding pocket ($d_{NH\text{-}O,}$ between the thienopyrimidinone core and the I109 backbone NH) (see also Fig. 2c, main text, movies 1-6, and Extended Material and Methods for details). For the strong Tpx dimerizers *para*-CFT, *meta*-CFT and CPT (1-2, 5, see a), we did not observe dissociation. The dimer interfaces mostly resembled our Tpx/*para*-CFT dimer crystal structure (6GXG) and were stable throughout 100 ns MD simulations. For the weak Tpx dimerizers *ortho*-CFT and CtFT (3, 4, see b), distortions of the dimer interface were frequently observed (e.g. inhibitors did not stack and/or the salt bridge could not form, quantified in Fig. 2d). We also monitored the distance between the tryptophan residues W39 and W70 within the same protomer (indicated by $d_W$ representing the distance between the W39 indole NH and the backbone oxygen of W70). Here, in a fraction of frames, the W39 side chain adopted a solvent exposed stance (termed “W39-flip”, exemplarily shown for Tpx/*meta*-CFT dimers in the “F-in”-orientation), that still allowed a dimer-productive interaction. c, Illustration of the “W39 flip”, quantified by the distance $d_W$ (criterion $d_W$ > 9 Å), which occurred in multiple Tpx dimer MD simulations but did not disrupt Tpx dimers. The “W39 flip” was also observed for corresponding monomers (Fig. S18). d, The number of MD simulation frames where W39 adopted a “flipped”, solvent exposed orientation is shown in a bar graph for each inhibitor. Occurrence of a “W39-flip” correlated with inhibitor size (non-fluorinated < monofluorinated < trifluoromethylated inhibitor) but not Tpx dimer affinity (high portion of frames for both the least efficient (CtFT, 4, green bar, dimer $K_D$= 410 ± 56 µM) and the best dimerizer (*meta*-CFT, 2, blue bar, dimer $K_D$= 3.7 ± 1.5 µM)). Interestingly, the capability of *ortho*-CFT (3) to displace the W39 side chain greatly varied depending on whether the fluorine atom pointed towards the binding pocket (“F-in”) or the solvent (“F-out”), and indicated that a fluorine in ortho-position, pointing towards the protein, weakens inhibitor attachment to the binding pocket. This was also observed in MD simulations of monomeric Tpx/*ortho*-CFT complexes (see Fig. 4b, first panel, and Fig. 5b).

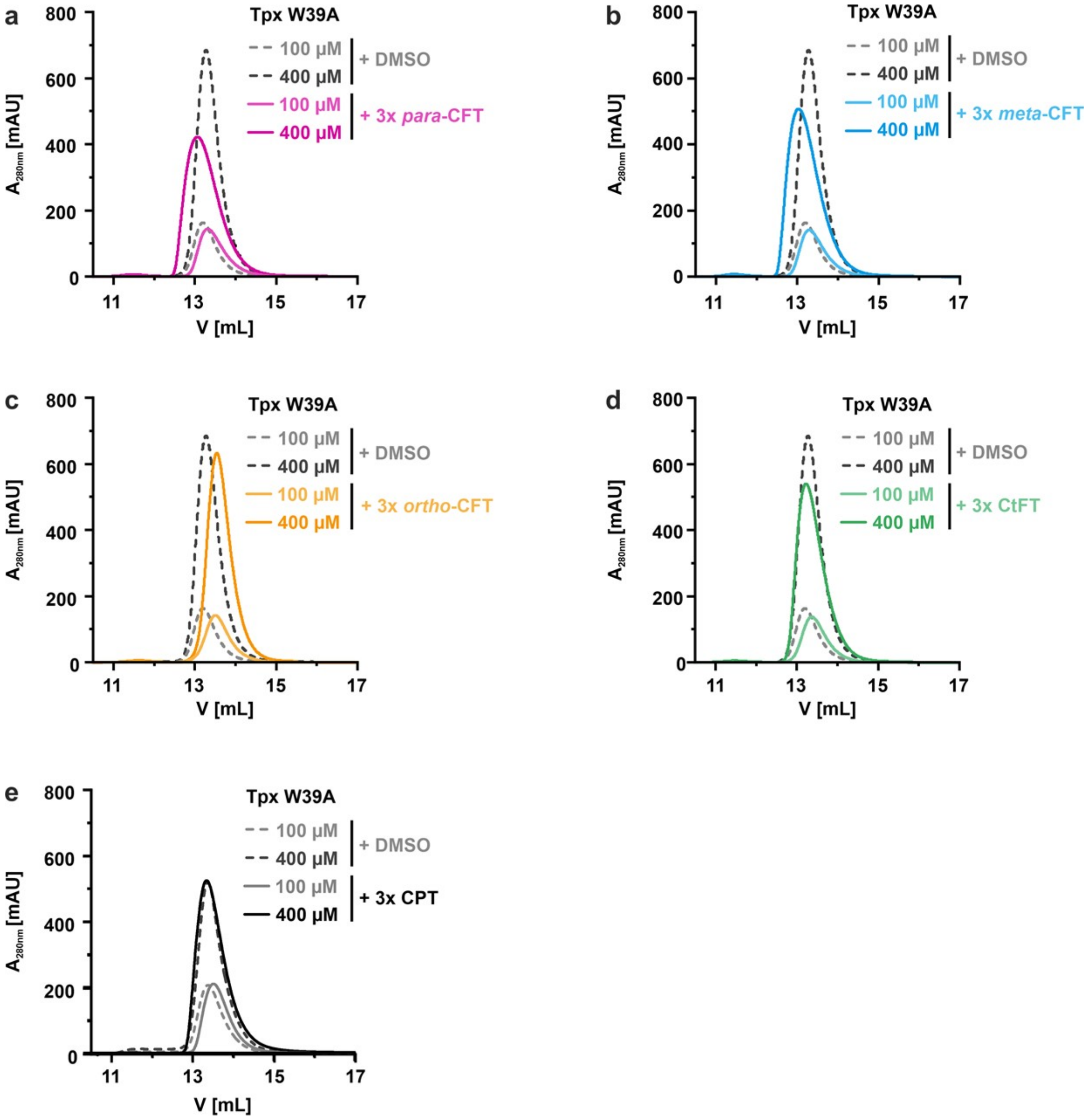


**Fig. S12.** Tpx W39A remains monomeric upon binding of compounds 1-5. a-e, Size exclusion chromatography (SEC) of Tpx W39A with three-fold molar excess of compounds 1-5 at 100 µM and 400 µM protein concentration. As a control, the elution profiles of reduced Tpx W39A with 7.5% DMSO (100 µM protein) or 30% DMSO (400 µM protein) in the absence of inhibitor were also recorded (n= 1). In all cases, the elution volumes were highly similar, indicating that the Tpx W39A mutant does not appreciably dimerize under the tested conditions.

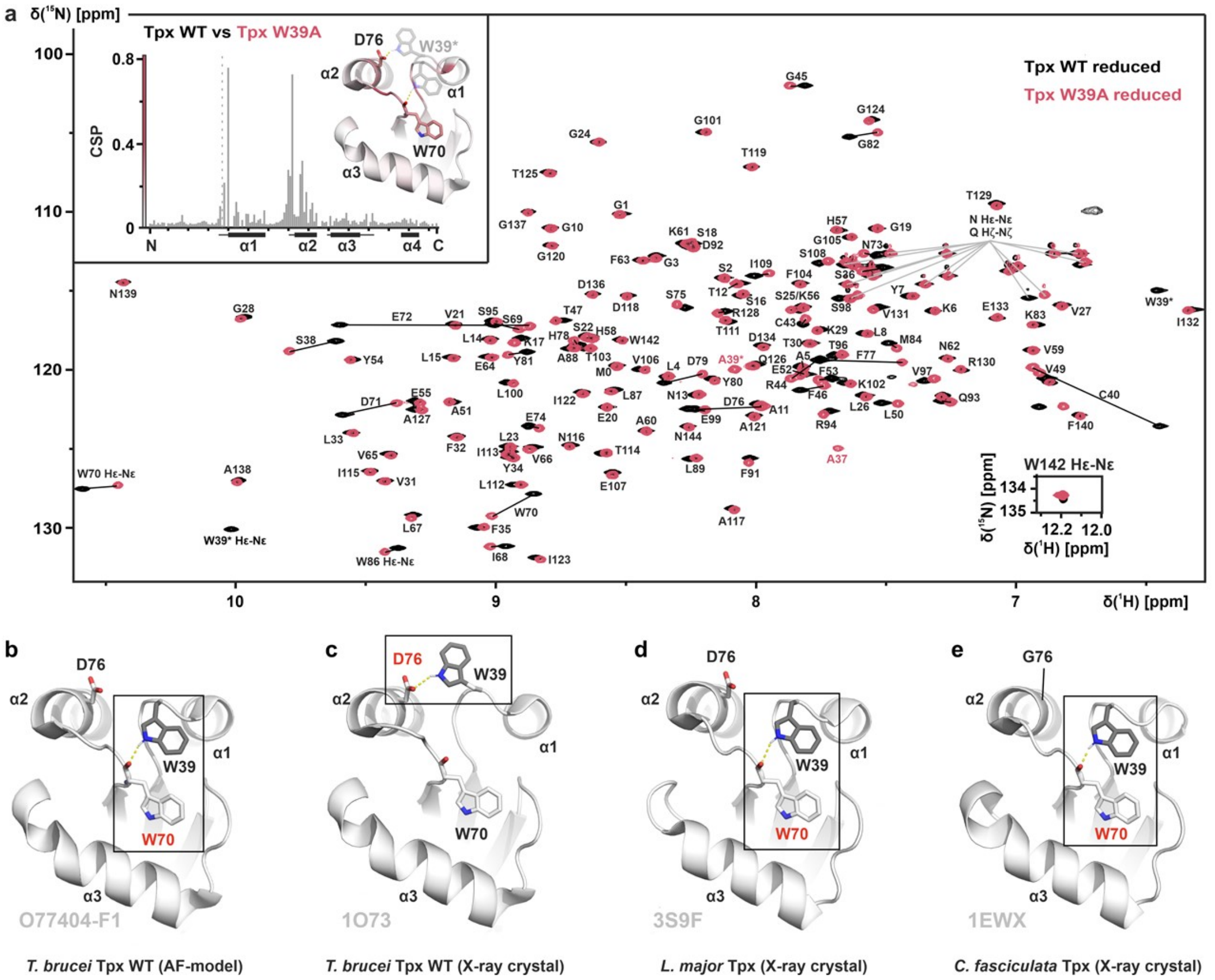


**Fig. S13.** Backbone NMR assignment of Tpx W39A and W39 side chain flexibility. a, $^{1}$H, $^{15}$N-HSQC spectra of Tpx WT (black) and Tpx W39A (red) in the reduced state, and mutation-induced chemical shift perturbation (CSP) analysis (inset). The backbone assignment for Tpx W39A could be partially obtained by transferring our previous assignment for the Tpx WT[2], and was verified and completed through additional 3D experiments (BMRB entry 53259). Resonances from the mutated residue 39 are marked with an asterisk. The inset shows the per residue CSP introduced by the W39A mutation which was mapped onto the protein sequence (left) and a structural model of Tpx (right) which is based on both the Alpha Fold model (AF-O77404-F1-model_v4[31,32]) and the *T. brucei* Tpx crystal structure (1O73[35]) to consider the structural ambiguity of residues 38-40 which is particularly pronounced for the W39 side chain orientation (b and c, respectively). The CSP plot (left) starts with residue G1 and ends with N144 (indicated with "N" and "C", respectively), ticks indicate increments of ten, starting with G10. The newly appearing signal for residue A37 (which is invisible in the WT spectrum) is indicated with a dashed line. The Tpx α-helices are indicated by black cylinders on the x-axis and adjacent loops with high CSP are indicated by lines. The tertiary structure model (right) shows W39 in two different conformations where it either forms a hydrogen bond to the W70 backbone (yellow dashed line, see b) or the D76 side chain oxygen (see c), which is in good agreement with residues that undergo the highest CSP. Heteroatom color scheme: N: blue; O: red. b-e, AlphaFold model of *T. brucei* Tpx WT (AF-O77404-F1-model_v4[31,32]), and crystal structures of Tpx WT from *T. brucei* (1O73[35]), *L. major* (3S9F[33]) and *C. fasciculata* (1EWX[34]). Side chains of W39, W70, and respective residues 76 are depicted as sticks (W39 in grey, W70 and residues 76 in white). In the *T. brucei* Tpx WT crystal structure (c), W39 adopts a conformation where its side chain is in a hydrogen bond with D76 ($d$= 2.6 Å, yellow dashed line) and fully solvent exposed, i.e. energetically unfavorable for a hydrophobic amino acid in aqueous solution. This clearly differs from the AlphaFold prediction (b) where W39 forms a hydrogen bond with the W70 backbone oxygen ($d$= 2.1 Å). As shown above in Fig. S11 c, d, the W39 side chain also did not adopt a solvent exposed stance throughout a 100 ns MD simulation of the apo state Tpx. Similarly, in crystal structures of orthologous Tpx from *L. major* and *C. fasciculata*, W39 is attached to the protein surface and forms a hydrogen bond with the carbonyl backbone of W70 ($d_w$= 2.4 Å and 2.0 Å, respectively, yellow dashed lines) regardless of the presence of an aspartate residue at position 76. Taken together, we thus hypothesize that in solution, the W39 side chain of unmodified *T. brucei* Tpx WT is predominantly hydrogen-bonded to the residue W70 backbone and that the solvent-exposed W39 side chain conformation in the available apo state X-ray structure (1O73[35]) reflects only a very small fraction of the conformational ensemble in solution.

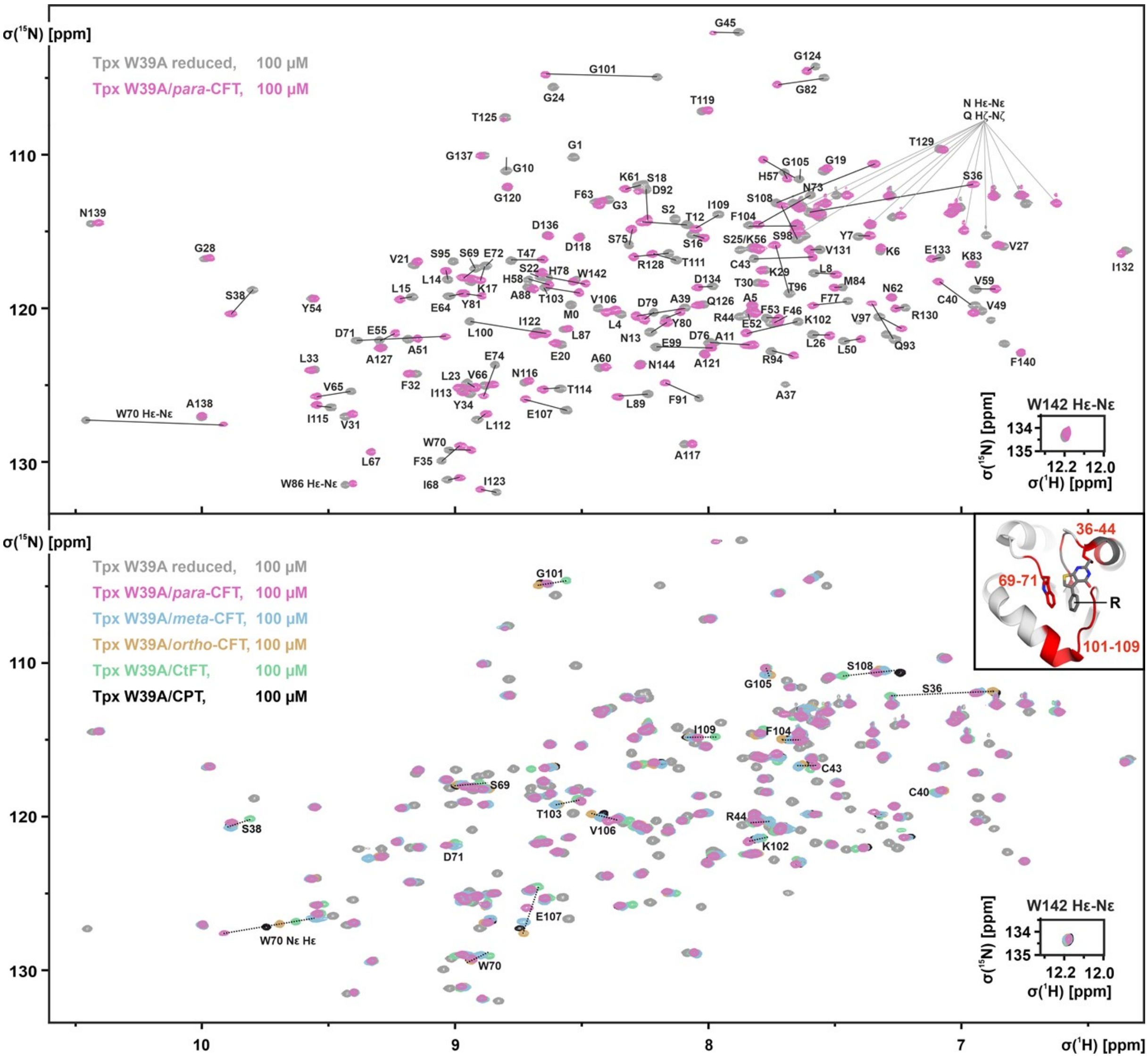


**Fig. S14.** Chemical shift perturbation of monomeric Tpx W39A upon binding of compounds 1-5. a, $^{1}H$, $^{15}N$-HSQC spectra of Tpx W39A in the reduced (black) and *para*-CFT-bound state (pink). The backbone assignments for reduced and *para*-CFT-bound Tpx W39A were transferred from our previous assignment for the reduced Tpx WT[2] and, where necessary, verified through additional 3D experiments. b, $^{1}H$, $^{15}N$-HSQC spectra of $^{15}N$-labeled Tpx W39A in the reduced (grey) and inhibitor-bound states (black, colored). The backbone assignments for Tpx W39A with bound inhibitors (2-5) were transferred from our assignments of *para*-CFT-bound Tpx W39A (see a) and, where necessary, verified through additional 3D experiments. Major CSPs are indicated by dotted lines and mostly belong to residues within the Tpx binding site (inset based on the Tpx/*para*-CFT crystal structure 6GXG[4], residues affected by inhibitor binding are shown in red). For CSP mapping onto the protein sequence and structure, see Fig. 3.

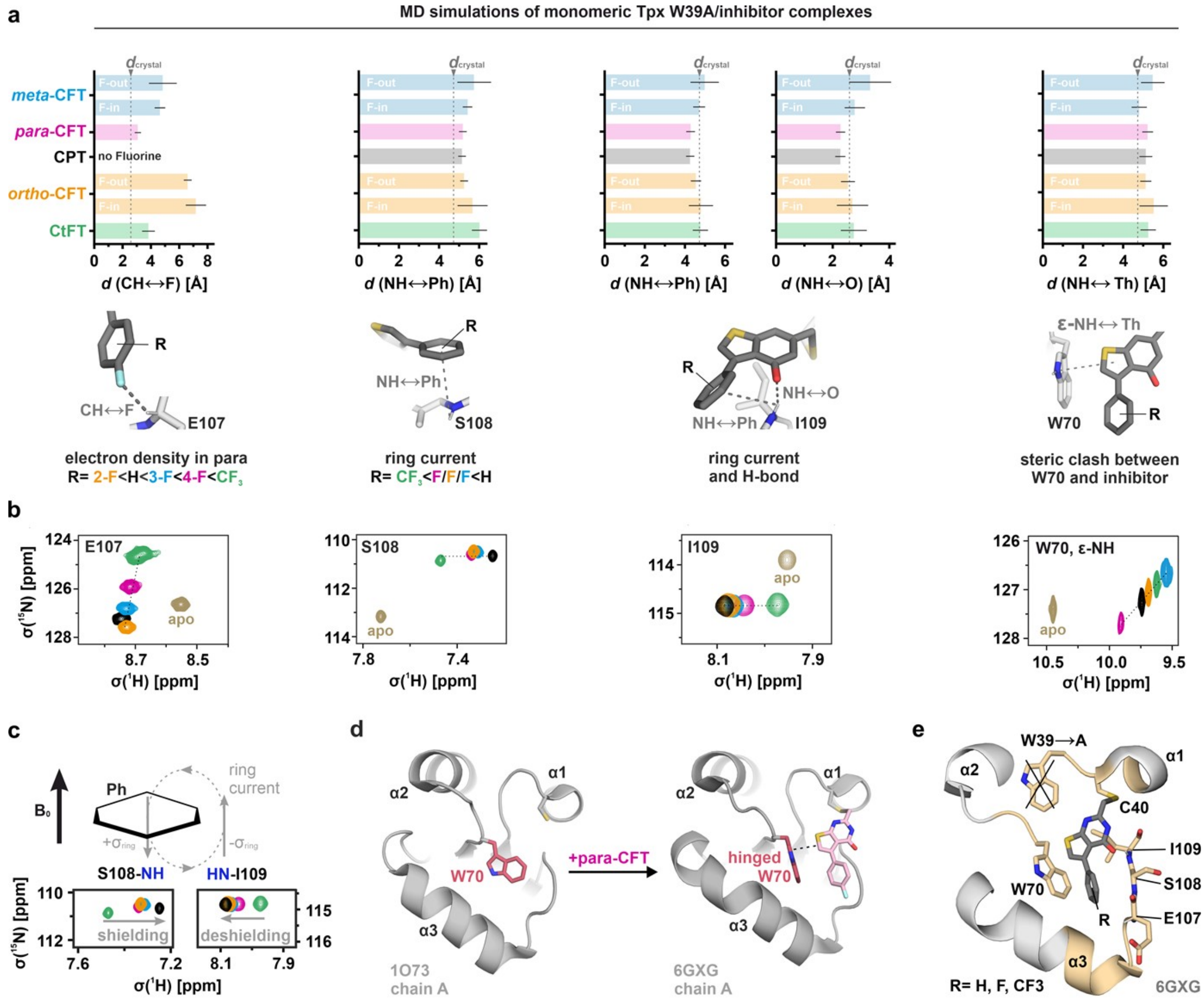


**Fig. S15.** Structural basis of inhibitor interactions with monomeric Tpx W39A. a, Analysis of MD simulations (n=5, 100 ns) of monomeric Tpx W39A/inhibitor complexes. Simulations were set up using chain A of the Tpx/*para*-CFT crystal structure (PDB: 6GXG[4]) and replacing W39 with alanine. The mean distances *d* between Tpx W39A/inhibitor contacts, including their standard error (n=5), are shown as bar graphs. For comparison, the corresponding distances observed in the Tpx WT/*para*-CFT crystal structure ($d_{crystal}$) are indicated by a dashed line. For clarity, contacts are visualized below. Hydrogen bonds and aromatic interactions between Tpx residues and inhibitor moieties (fluorine, phenyl, oxygen, and thiophen) are indicated by dashed lines. Throughout the simulations, all inhibitors (1-5) remained in a pose similar to the Tpx/*para*-CFT crystal structure ($d_{crystal}$), with slight deviations for *meta*-CFT and CtFT (2, 4), showing that the crystal structure can serve as a structural template to model Tpx W39A. As expected, the distance between the respective inhibitor fluorine atom(s) to the $C_\alpha$-proton of E107 (i) varied the most due to the different fluorination patterns ($d_{para}$<$d_{meta}$<$d_{ortho}$). Heteroatom color scheme: N: blue; O: red; S: yellow; F: light blue. b, Amide resonances of selected amino acids reflect interactions with the inhibitors' fluorine, phenyl, oxygen, and thiophene moieties (shown above). The E107 backbone amide chemical shift trajectory depended on the electron density at the inhibitor's *para*-position. This is highest for CtFT (4, green) due to its electron-dense $CF_3$-group, and lowest for *ortho*-CFT (3, orange), where the Fluorine-substituent deprives the inhibitor phenyl ring of electron density in the *para*-position, a loss that except for *meta*-CFT (2), cannot be compensated via the mesomeric (+M) effect. Similarly, S108 backbone amide shielding correlated with the electron density of the inhibitor's phenyl ring, an effect most pronounced for the non-fluorinated, electron-rich phenyl moiety in CPT (5, black), and least pronounced for CtFT (4, green) with its electron-poor phenyl π-system. Not surprisingly, the S108 backbone NH chemical shift was nearly identical for all monofluorinated inhibitors 1-3. The I109 backbone amide trajectory probably resulted from a combination of phenyl ring current effects and hydrogen bonding with the inhibitor, and intriguingly showed the opposite trajectory compared to the S108 residue. We propose that selective shielding and deshielding of the two neighboring residues S108 and I109 by a magnetically induced inhibitor phenyl ring current could explain this phenomenon (visualized in c). W70 side chain NH shifts did not correlate with the inhibitor's phenyl ring electron density, likely due to side chain rotations induced by the inhibitor as seen in our crystal structure of the Tpx/*para*-CFT complex, that can strongly affect interactions with the inhibitor (visualized in d). Overall, the fluorination pattern directly affects how an inhibitor interacts with the binding site residues. Importantly, these effects can be graded as seen here for selected examples based on the electron density of the phenyl ring or the position of the fluorine group. c, The backbone amide resonances of S108 and I109 displayed opposing chemical shift trajectories with inhibitors 1-5. We propose that an applied magnetic field $B_0$ induces an orthogonal phenyl ring current[41] that shields the backbone NH of S108, which is located underneath the phenyl ring, and deshields the I109 backbone NH next to it. As expected, these effects are least pronounced for the electron deprived phenyl ring carrying a trifluoromethyl group (CtFT 4, green), most pronounced for the non-fluorinated inhibitor CPT (5, black), and nearly identical for the mono-fluorinated inhibitors *para*-, *meta*- and *ortho*-CFT (1-3, pink, blue, orange). d, Crystallographic data on the Tpx W70 side chain orientation in the apo (1O73)[35] and *para*-CFT (1)-bound states (6GXG[4], chain A).[4] Once the inhibitor attaches to the protein surface, it induces a ~90° rotation of the W70 side chain, pushing it

towards the α3-helix. W70 can thus be considered a “wing gate”[42], explaining the consequences of inhibitor binding for the chemical environment and the resulting NMR chemical shift of the W70 side chain NH (see b, right panel). In all MD simulations of Tpx/inhibitor complexes, which were set up with the “hinged” W70 pose from the Tpx/*para*-CFT dimer structure (6GXG[4], chains A, B, “wing gate open”), the overall W70 orientation did not transition to the apo orientation (seen in crystal structures 1O73[35] “wing gate closed”) (see W70 orientations in Fig. S18, movies 1-14). e, Structural model of the monomeric TpxW39A/inhibitor complex showing the consensus inhibitor binding pocket (sand) for covalently attached compounds 1-5, derived from the chemical shift perturbation pattern in $^{1}H,^{15}N$-NMR experiments and MD simulations. The binding pocket comprises the N-terminal loops of α-helix1 (active site) and α-helix2 (containing W70), as well as the C-terminal end of α-helix3 and the adjacent loop containing residues E107, S108, and I109.

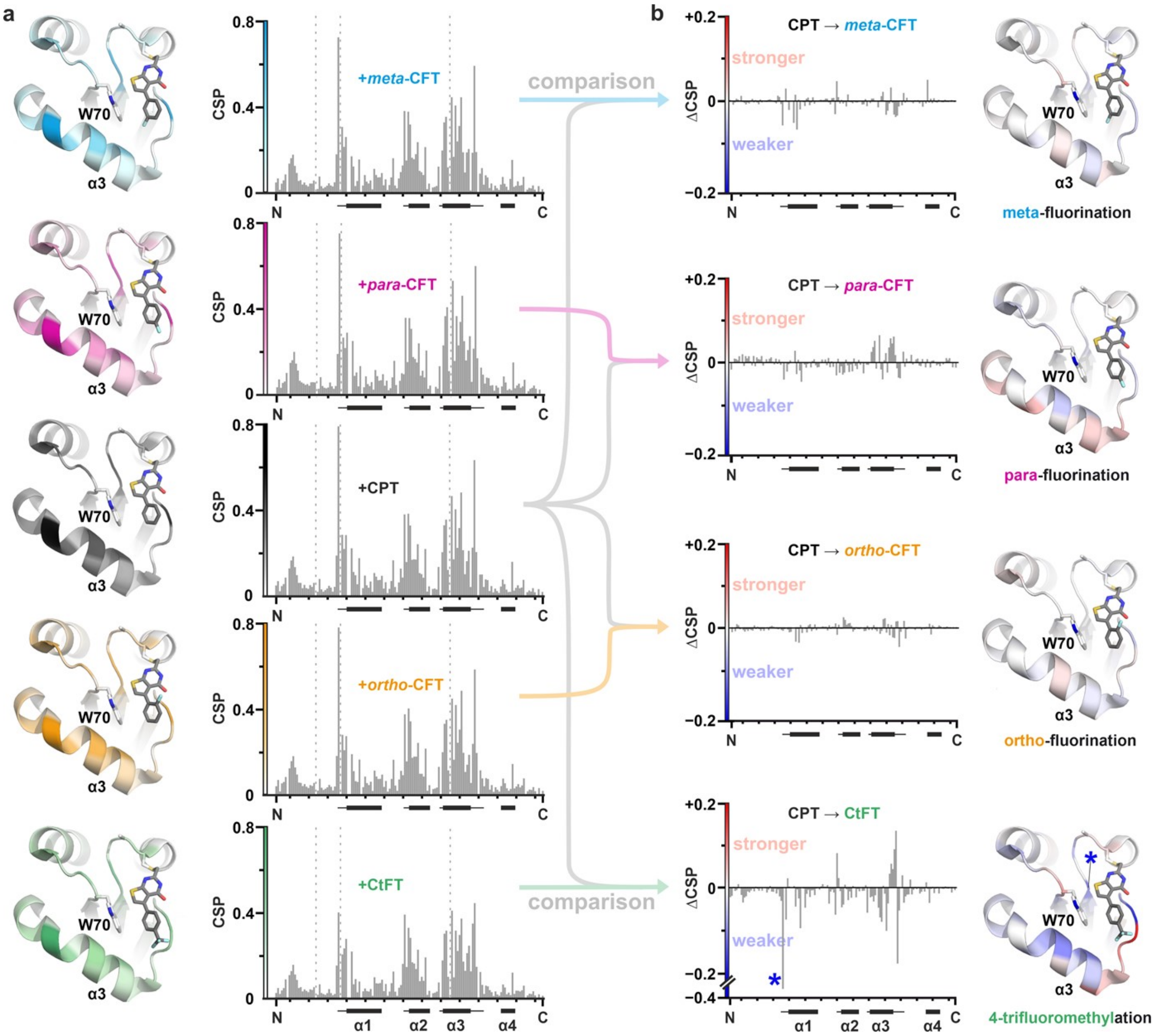


**Fig. S16:** A comparison of inhibitor-induced backbone chemical shift perturbations (CSPs) of Tpx W39A reveals fluorination-mediated protein/inhibitor interactions. (a) CSPs of monomeric Tpx W39A upon inhibitor binding. In the bar graphs, line broadening is indicated by dashed bars. A-helices are indicated as rectangles underneath each plot. (b) Pairwise chemical shift perturbation differences (ΔCSP) between non-fluorinated CPT (**5**) and fluorinated analogs (**1**-**4**). Values of 0 indicate no CSP difference between respective fluorinated and the non-fluorinated inhibitor CPT (shown in white on the structure). Values greater or lower than 0 indicate an enhanced or reduced effect on the respective residue due to inhibitor fluorination (shown in red and blue, respectively).

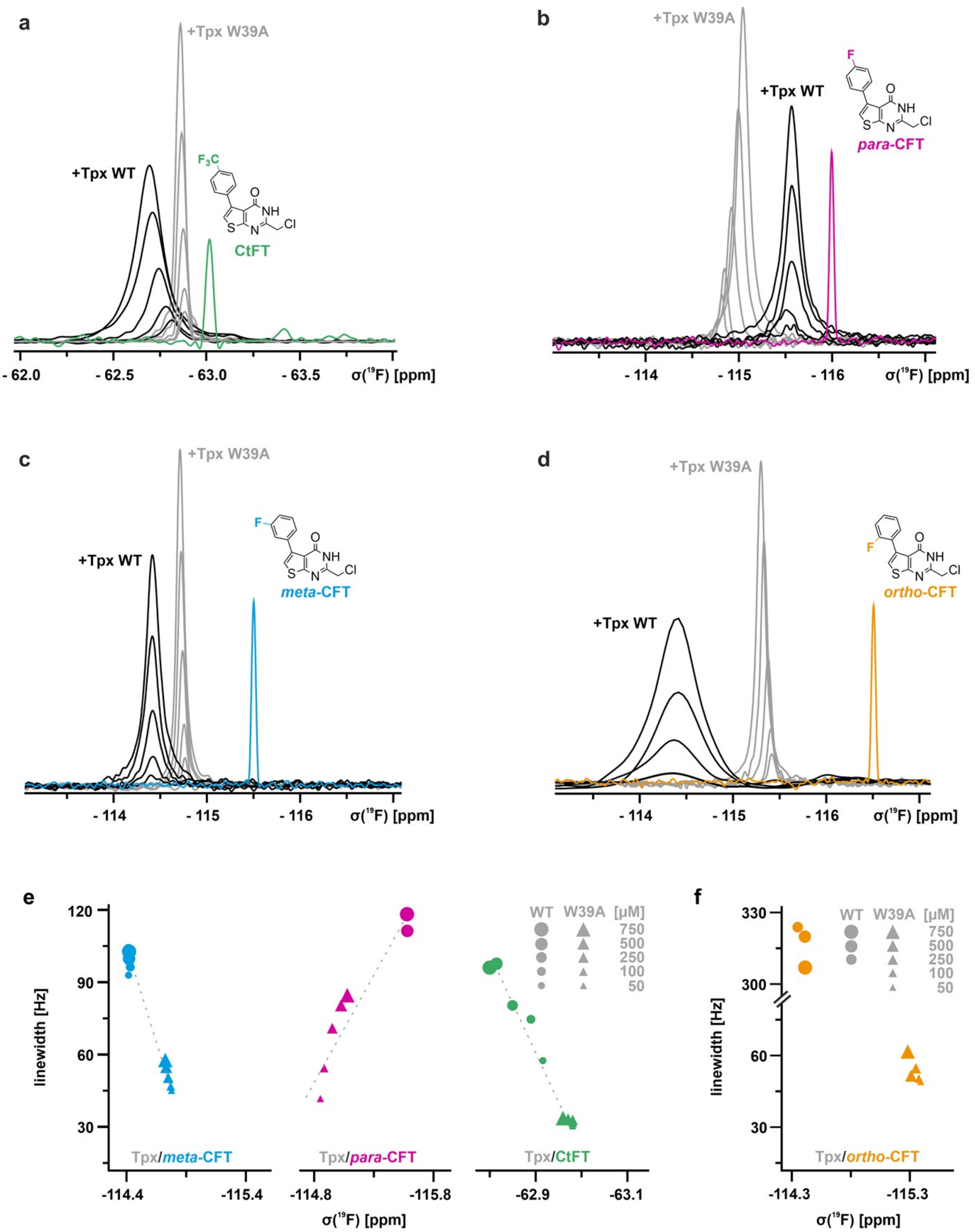


**Fig. S17.** The 19F-NMR signals of free and protein-bound inhibitors provide information about dimer dissociation and bound molecular glue dynamics. a-d, 1D-19F-NMR spectra of free (100 µM, shown in the respective color) and Tpx-bound (750, 500, 250, 100 and 50 µM each; WT: black, W39A: grey) inhibitors 1-4. 19F-NMR spectra were referenced to TFA and, where DMSO was added, the chemical shift was corrected accordingly (see also Table S4). The signal of the Tpx WT/*ortho*-CFT complex at 50 µM is not shown due to poor signal to noise ratio. e,f Chemical shifts and linewidths of 19F-NMR signals of Tpx-bound compounds 1–4 at different protein concentrations (see also Table S4). Data points of Tpx WT with bound *para*- and *ortho*-CFT (1, 3) at low concentrations (50 and 100 µM, respectively) are not shown due to poor signal-to-noise ratios.

Note that 19F-NMR signals of Tpx WT complexes with strong dimerizers (**1-3, 5**) remained nearly unaffected by dilution as expected for dimers with low-micromolar $K_D$s. In contrast, the 19F-NMR signal linewidths and chemical shifts of Tpx/inhibitor complexes with weak dimer affinities, (Tpx WT/CtFT and Tpx W39A/*para*-CFT), consistently changed since the chosen concentration range was lower than the dimer $K_D$s.

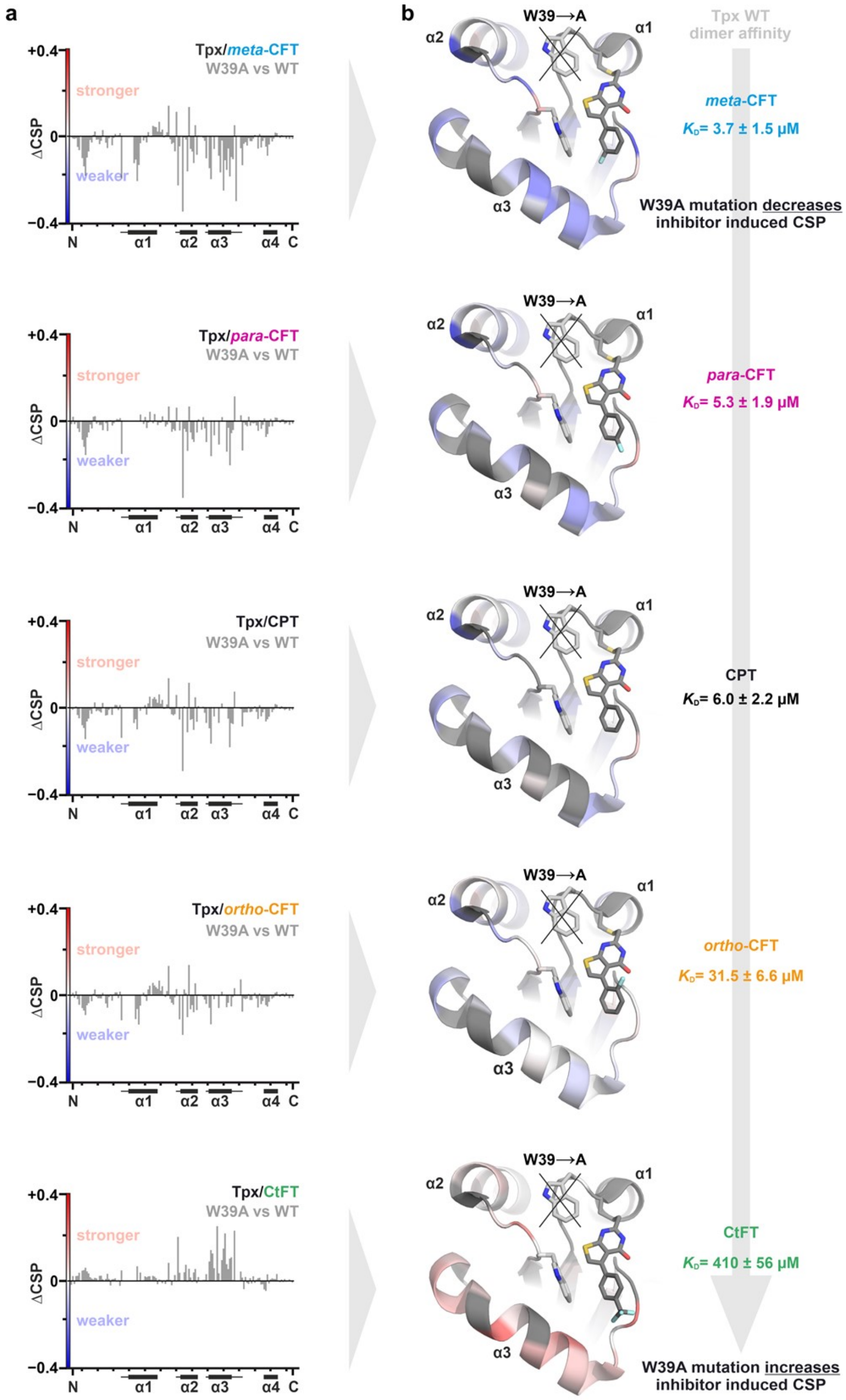


**Fig. S18.** Comparison of NMR chemical shifts of inhibitor-bound Tpx W39A and Tpx WT. a, Inhibitor-induced CSP of Tpx WT were subtracted from the inhibitor-induced CSP of Tpx W39A to yield ΔCSP for each inhibitor (1-5). ΔCSP is shown as bar graphs (left), starting with residue G1 and ending with N144 (indicated with "N" and "C"), ticks indicate increments of ten, starting with G10. Residues without evaluable NMR signals (e.g. due to peak broadening) were arbitrarily set to 0. b, ΔCSP mapped onto the inhibitor binding site of Tpx (model based on the Tpx/*para*-CFT crystal structure 6GXG[4], chain A). Residues with no ΔCSP are shown in white, and residues without evaluable NMR signals (e.g. due to peak broadening) are depicted in grey. For the strong dimerizer *meta*-CFT (2), ΔCSP is mainly negative (shown in blue) because dimerization, which strongly contributes to the overall CSP, is suppressed in Tpx W39A. Conversely, ΔCSP for the monomeric Tpx WT complex with CtFT (4) is positive (shown in red), indicative of a stronger inhibitor interaction with the protein surface once the W39 indole ring is removed via mutation. This suggests a gatekeeping character for Tpx W39 (see main text for details). Heteroatom color scheme: N: blue; O: red; S: yellow; F: light blue.

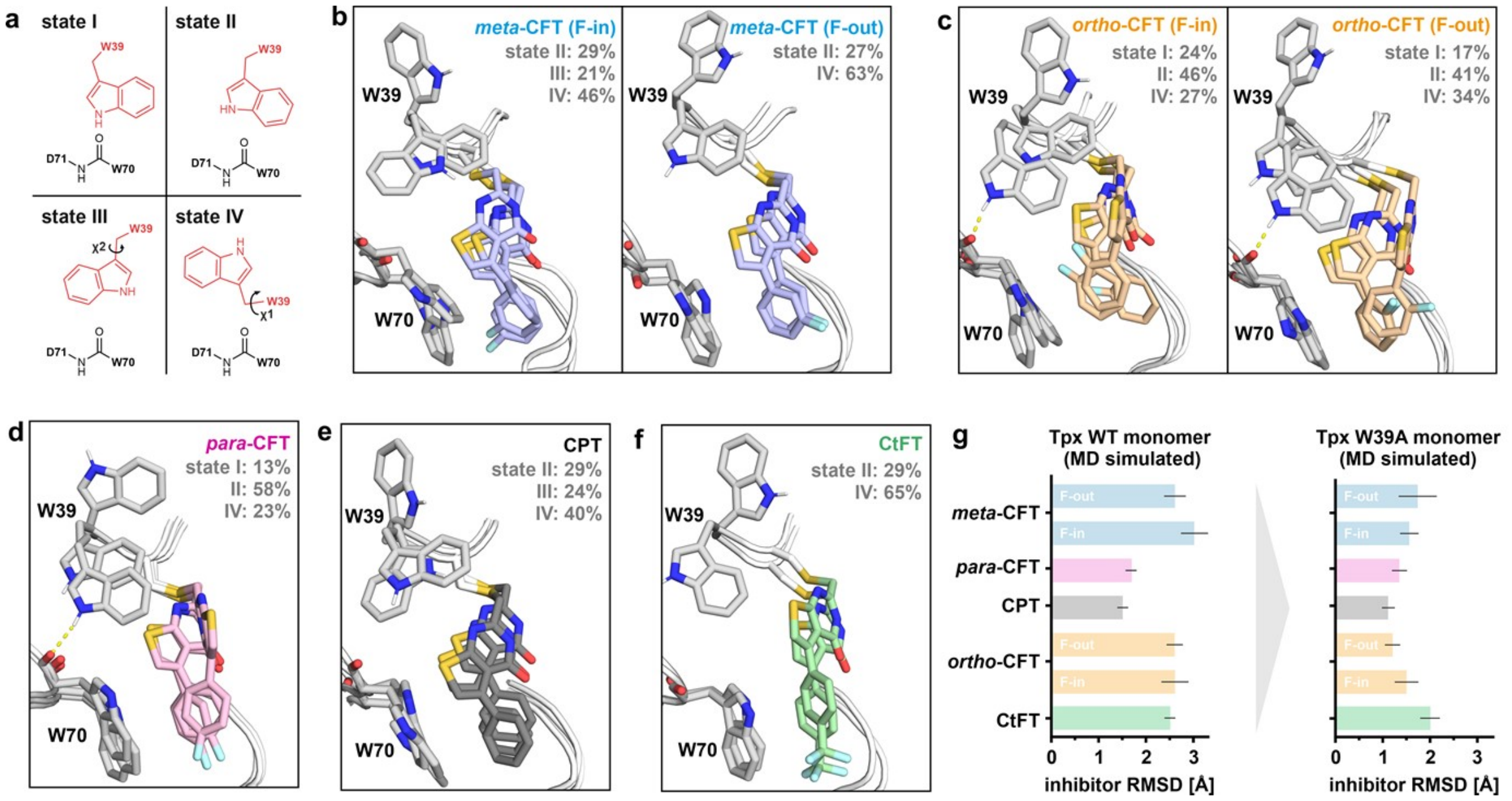


**Fig. S19.** Dynamic inhibitor interactions with aromatic surface residues W39 and W70. a, Schematic depiction of Tpx W39 states (I-IV) observed in MD simulations of monomeric Tpx/inhibitor complexes (100 ns, n=5) which were set up using the crystal structure of Tpx WT with bound *para*-CFT (PDB: 6GXG[4]) as a starting model. State I shows W39 in a hydrogen bond with the W70 backbone, which is missing in state II. In state III, the W39 indole side chain is flipped "sideways", and in state IV "upwards" which leaves the indole ring fully solvent exposed (see also Fig. S13). Notably, once state III was adopted, it persisted throughout the remainder of the MD simulations, suggesting an energetic minimum (movies 10, 13). For quantification of states I-IV, we analyzed the distance between the Tpx W39 indole NH and the backbone oxygen of residue W70 (see Fig. 4 and extended Material and Methods for details). Comparison to available crystal structures for oxidized[35] and inhibitor-bound[4] *T. brucei* Tpx WT, the AlphaFold2 model of the oxidized enzyme[31,32] agreed with the substantial structural plasticity for the W39 side chain (Fig. S13). b-f: Snapshots of the Tpx/inhibitor binding interface illustrating conformational states that occurred in at least 10% of all MD simulation frames of a given Tpx/inhibitor complex. Inhibitor fluorination greatly affected the dynamics of the W39 and W70 side chains and inhibitors in the Tpx binding site, which is also reflected in the relative proportion of states I-IV for each inhibitor (shown in the top right of each panel and Fig. 4b-c). In sum, the side chain of W39 is much more dynamic (see also Fig. S13) than the W70 side chain, which mostly remains in a "hinged" orientation (see Fig. S15) allowing close inhibitor attachment to the consensus Tpx binding pocket. Of note, the weak dimerizers *ortho*-CFT (especially in "F-in" orientation) and CtFT (3, 4) occurred more often in a loosely attached pose (movies 11-13) than the other inhibitors, which adapted tightly protein-attached poses (movies 8-10, 14) similar to the inhibitor stance in the Tpx/*para*-CFT crystal structure (Fig. S15e). Highlighting the greater importance of molecular glue positioning than the overall W39 sidechain orientation, our MD simulations showed that stable dimer interfaces can form regardless of the W39 χ-angles (Fig. S11, movies 1-7) and that both the strongest and weakest dimerizers *meta*-CFT (2) and CtFT (4) favor state IV with a solvent-exposed W39 sidechain (Fig. 4b-c, blue and green bars). However, while *meta*-CFT (2) efficiently inserts into the binding pocket when the W39 side chain has cleared the way, the bulky $CF_3$-group in CtFT (4) still prevented proper ligand positioning and thus a dimerization-promoting inhibitor stance (see panels b, f). Hydrogen bonds are shown as yellow dashed lines, heteroatom color scheme: N: blue; O: red; S: yellow; F: light blue. g, In MD simulations of monomeric Tpx/inhibitor complexes (100 ns, n = 5), inhibitors are more dynamic when bound to Tpx WT than to the W39A mutant, i.e. show a higher RMSD compared to the starting structure (PDB: 6GXG[4], chain A). This highlights the crucial role of the W39 sidechain in bound inhibitor dynamics and attachment to the Tpx binding site.

## F. Supporting Information References


1 J. Marley, M. Lu and C. Bracken, *J Biomol NMR*, 2001, **20**, 71.

2 A. Wagner, E. Diehl, R. L. Krauth-Siegel and U. A. Hellmich, *Biomol NMR Assign*, 2017, **11**, 193.

3 M. T. Marty, A. J. Baldwin, E. G. Marklund, G. K. A. Hochberg, J. L. P. Benesch and C. V. Robinson, *Analytical chemistry*, 2015, **87**, 4370.

4 A. Wagner, T. A. Le, M. Brennich, P. Klein, N. Bader, E. Diehl, D. Paszek, A. K. Weickhmann, N. Dirdjaja, R. L. Krauth-Siegel, B. Engels, T. Opatz, H. Schindelin and U. A. Hellmich, *Angewandte Chemie International Edition*, 2019, **58**, 3640.

5 F. Fueller, B. Jehle, K. Putzker, J. D. Lewis and R. L. Krauth-Siegel, *The Journal of biological chemistry*, 2012, **287**, 8792.

6 J. O'Brien, I. Wilson, T. Orton and F. Pognan, *European Journal of Biochemistry*, 2000, **267**, 5421.

7 J. M. Strelow, *SLAS Discovery*, 2017, **22**, 3.

8 U. Hellberg, J.-P. Ivarsson and B.-L. Johansson, *Process Biochemistry*, 1996, **31**, 163.

9 B. H. Zimm, *J. Chem. Phys.*, 1948, **16**, 1093.

10 S. P. Skinner, R. H. Fogh, W. Boucher, T. J. Ragan, L. G. Mureddu and G. W. Vuister, *J Biomol NMR*, 2016, **66**, 111.

11 F. A. Mulder, D. Schipper, R. Bott and R. Boelens, *Journal of Molecular Biology*, 1999, **292**, 111.

12 A. D. Becke, *J. Chem. Phys.*, 1993, **98**, 5648.

13 S. Grimme, J. Antony, S. Ehrlich and H. Krieg, *J. Chem. Phys.*, 2010, **132**, 154104.

14 a) Y. Zhao and D. G. Truhlar, *Theor Chem Account*, 2008, **120**, 215; b) F. Weigend and R. Ahlrichs, *Physical chemistry chemical physics : PCCP*, 2005, **7**, 3297; c) A. V. Marenich, C. J. Cramer and D. G. Truhlar, *The journal of physical chemistry. B*, 2009, **113**, 6378;

15 M. J. Frisch, G. W. Trucks, H. B. Schlegel, G. E. Scuseria, M. A. Robb, J. R. Cheeseman, G. Scalmani, V. Barone, G. A. Petersson, H. Nakatsuji, X. Li, M. Caricato, A. V. Marenich, J. Bloino, B. G. Janesko, R. Gomperts, B. Mennucci, H. P. Hratchian, J. V. Ortiz, A. F. Izmaylov, J. L. Sonnenberg, D. Williams-Young, F. Ding, F. Lipparini, F. Egidi, J. Goings, B. Peng, A. Petrone, T. Henderson, D. Ranasinghe, V. G. Zakrzewski, J. Gao, N. Rega, G. Zheng, W. Liang, M. Hada, M. Ehara, K. Toyota, R. Fukuda, J. Hasegawa, M. Ishida, T. Nakajima, Y. Honda, O. Kitao, H. Nakai, T. Vreven, K. Throssell, Montgomery, J. A., Jr., J. E. Peralta, F. Ogliaro, M. J. Bearpark, J. J. Heyd, E. N. Brothers, K. N. Kudin, V. N. Staroverov, T. A. Keith, R. Kobayashi, J. Normand, K. Raghavachari, A. P. Rendell, J. C. Burant, S. S. Iyengar, J. Tomasi, M. Cossi, J. M. Millam, M. Klene, C. Adamo, R. Cammi, J. W. Ochterski, R. L. Martin, K. Morokuma, O. Farkas, J. B. Foresman and D. J. Fox, *Gaussian˜16 Revision C.01*, 2016.

16 A. W. Da Sousa Silva and W. F. Vranken, *BMC Res Notes*, 2012, **5**, 367.

17 J. A. Maier, C. Martinez, K. Kasavajhala, L. Wickstrom, K. E. Hauser and C. Simmerling, *Journal of chemical theory and computation*, 2015, **11**, 3696.

18 W. L. Jorgensen, J. Chandrasekhar, J. D. Madura, R. W. Impey and M. L. Klein, *J. Chem. Phys.*, 1983, **79**, 926.

19 a) H. Bekker, H. Berendsen, E. Dijkstra, S. Achterop and M. Renardus, *Gromacs: A parallel computer for molecular dynamics simulations*, 1993, vol. 92; b) H. Berendsen, D. van der Spoel and R. van Drunen, *Computer Physics Communications*, 1995, **91**, 43; c) D. van der Spoel, E. Lindahl, B. Hess, G. Groenhof, A. E. Mark and H. J. C. Berendsen, *Journal of Computational Chemistry*, 2005, **26**, 1701; d) S. Páll, M. J. Abraham, C. Kutzner, B. Hess and E. Lindahl, *Proc. EASC*, **8759**, 3; e) M. J. Abraham, T. Murtola, R. Schulz, S. Páll, J. C. Smith, B. Hess and E. Lindahl, *SoftwareX*, 2015, **1-2**, 19;

20 G. Bussi, D. Donadio and M. Parrinello, *J. Chem. Phys.*, 2007, **126**, 14101.

21 M. Parrinello and A. Rahman, *J. Appl. Phys.*, 1981, **52**, 7182.
22 B. Hess, H. Bekker, H. J. C. Berendsen and J. G. E. M. Fraaije, *J. Comput. Chem.*, 1997, **18**, 1463.
23 G. van Rossum, *The Python language reference*, Python Software Foundation; SoHo Books, Hampton, NH, Redwood City, Calif., 3rd edn., 2010, Pt. 2.
24 *Anaconda Software Distribution*, Anaconda Inc, 2020, *Anaconda Documentation,* https://docs.anaconda.com/.
25 The Matplotlib Development Team, Matplotlib: Visualization with Python, Zenodo, 2023.
26 C. R. Harris, K. J. Millman, S. J. van der Walt, R. Gommers, P. Virtanen, D. Cournapeau, E. Wieser, J. Taylor, S. Berg, N. J. Smith, R. Kern, M. Picus, S. Hoyer, M. H. van Kerkwijk, M. Brett, A. Haldane, J. F. Del Río, M. Wiebe, P. Peterson, P. Gérard-Marchant, K. Sheppard, T. Reddy, W. Weckesser, H. Abbasi, C. Gohlke and T. E. Oliphant, *Nature*, 2020, **585**, 357.
27 V. Tormyshev, D. Trukhin, O. Rogozhnikova, T. Mikhalina, T. Troitskaya and A. Flinn, *Synlett*, 2006, **2006**, 2559.
28 P. M. Titchenell, H. D. Showalter, J.-F. Pons, A. J. Barber, Y. Jin and D. A. Antonetti, *Bioorganic & Medicinal Chemistry Letters*, 2013, **23**, 3034.
29 K. V. Mahasenan, M. Bastian, M. Gao, E. Frost, D. Ding, K. Zorina-Lichtenwalter, J. Jacobs, M. A. Suckow, V. A. Schroeder, W. R. Wolter, M. Chang and S. Mobashery, *ACS medicinal chemistry letters*, 2017, **8**, 654.
30 R. Adepu, R. Sunke, C. L. T. Meda, D. Rambabu, G. R. Krishna, C. M. Reddy, G. S. Deora, K. V. L. Parsa and M. Pal, *Chemical communications (Cambridge, England)*, 2013, **49**, 190.
31 J. Jumper, R. Evans, A. Pritzel, T. Green, M. Figurnov, O. Ronneberger, K. Tunyasuvunakool, R. Bates, A. Žídek, A. Potapenko, A. Bridgland, C. Meyer, S. A. A. Kohl, A. J. Ballard, A. Cowie, B. Romera-Paredes, S. Nikolov, R. Jain, J. Adler, T. Back, S. Petersen, D. Reiman, E. Clancy, M. Zielinski, M. Steinegger, M. Pacholska, T. Berghammer, S. Bodenstein, D. Silver, O. Vinyals, A. W. Senior, K. Kavukcuoglu, P. Kohli and D. Hassabis, *Nature*, 2021, **596**, 583.
32 M. Varadi, D. Bertoni, P. Magana, U. Paramval, I. Pidruchna, M. Radhakrishnan, M. Tsenkov, S. Nair, M. Mirdita, J. Yeo, O. Kovalevskiy, K. Tunyasuvunakool, A. Laydon, A. Žídek, H. Tomlinson, D. Hariharan, J. Abrahamson, T. Green, J. Jumper, E. Birney, M. Steinegger, D. Hassabis and S. Velankar, *Nucleic Acids Res*, 2024, **52**, D368-D375.
33 A. Fiorillo, G. Colotti, A. Boffi, P. Baiocco and A. Ilari, *PLoS neglected tropical diseases*, 2012, **6**, e1781.
34 B. Hofmann, H. Budde, K. Bruns, S. A. Guerrero, H. M. Kalisz, U. Menge, M. Montemartini, E. Nogoceke, P. Steinert, J. B. Wissing, L. Flohé and H. J. Hecht, *Biological Chemistry*, 2001, **382**, 459.
35 M. S. Alphey, M. Gabrielsen, E. Micossi, G. A. Leonard, S. M. McSweeney, R. B. G. Ravelli, E. Tetaud, A. H. Fairlamb, C. S. Bond and W. N. Hunter, *Journal of Biological Chemistry*, 2003, **278**, 25919.
36 A. A. Berger, J.-S. Völler, N. Budisa and B. Koksch, *Accounts of Chemical Research*, 2017, **50**, 2093.
37 E. A. Meyer, R. K. Castellano and F. Diederich, *Angewandte Chemie (International ed. in English)*, 2003, **42**, 1210.
38 Y. Zhao, J. Li, H. Gu, D. Wei, Y. Xu, W. Fu and Z. Yu, *Interdiscip Sci Comput Life Sci*, 2015, **7**, 211.
39 X. Chen, G. Wang, X. Zeng, W. Li and M. Zhou, *J. Am. Chem. Soc.*, 2025.
40 S. A. Harry, M. Kazim, P. M. Nguyen, A. Zhu, M. R. Xiang, J. Catazaro, M. Siegler and T. Lectka, *Angewandte Chemie International Edition*, 2022, **61**, e202207966.
41 M. Baranac-Stojanović, *RSC Adv*, 2014, **4**, 308.
42 A. Gora, J. Brezovsky and J. Damborsky, *Chemical Reviews*, 2013, **113**, 5871.
